# Single-dose administration and the influence of the timing of the booster dose on immunogenicity and efficacy of ChAdOx1 nCoV-19 (AZD1222) vaccine: a pooled analysis of four randomised trials

**DOI:** 10.1016/S0140-6736(21)00432-3

**Published:** 2021-03-06

**Authors:** Merryn Voysey, Sue Ann Costa Clemens, Shabir A Madhi, Lily Y Weckx, Pedro M Folegatti, Parvinder K Aley, Brian Angus, Vicky L Baillie, Shaun L Barnabas, Qasim E Bhorat, Sagida Bibi, Carmen Briner, Paola Cicconi, Elizabeth A Clutterbuck, Andrea M Collins, Clare L Cutland, Thomas C Darton, Keertan Dheda, Christina Dold, Christopher J A Duncan, Katherine R W Emary, Katie J Ewer, Amy Flaxman, Lee Fairlie, Saul N Faust, Shuo Feng, Daniela M Ferreira, Adam Finn, Eva Galiza, Anna L Goodman, Catherine M Green, Christopher A Green, Melanie Greenland, Catherine Hill, Helen C Hill, Ian Hirsch, Alane Izu, Daniel Jenkin, Carina C D Joe, Simon Kerridge, Anthonet Koen, Gaurav Kwatra, Rajeka Lazarus, Vincenzo Libri, Patrick J Lillie, Natalie G Marchevsky, Richard P Marshall, Ana V A Mendes, Eveline P Milan, Angela M Minassian, Alastair McGregor, Yama F Mujadidi, Anusha Nana, Sherman D Padayachee, Daniel J Phillips, Ana Pittella, Emma Plested, Katrina M Pollock, Maheshi N Ramasamy, Adam J Ritchie, Hannah Robinson, Alexandre V Schwarzbold, Andrew Smith, Rinn Song, Matthew D Snape, Eduardo Sprinz, Rebecca K Sutherland, Emma C Thomson, M Estée Török, Mark Toshner, David P J Turner, Johan Vekemans, Tonya L Villafana, Thomas White, Christopher J Williams, Alexander D Douglas, Adrian V S Hill, Teresa Lambe, Sarah C Gilbert, Andrew J Pollard, Oxford COVID Vaccine Trial Group, Marites Aban, Kushala W.M. Abeyskera, Jeremy Aboagye, Matthew Adam, Kirsty Adams, James P. Adamson, Gbadebo Adewatan, Syed Adlou, Khatija Ahmed, Yasmeen Akhalwaya, Saajida Akhalwaya, Andrew Alcock, Aabidah Ali, Elizabeth R. Allen, Lauren Allen, Felipe B. Alvernaz, Fabio Santos Amorim, Claudia Sala Andrade, Foteini Andritsou, Rachel Anslow, Edward H. Arbe-Barnes, Mark P. Ariaans, Beatriz Arns, Laiana Arruda, Luiza Assad, Paula De Almeida Azi, Lorena De Almeida Azi, Gavin Babbage, Catherine Bailey, Kenneth F. Baker, Megan Baker, Natalie Baker, Philip Baker, Ioana Baleanu, Danieli Bandeira, Anna Bara, Marcella A.S. Barbosa, Debbie Barker, Gavin D. Barlow, Eleanor Barnes, Andrew S. Barr, Jordan R. Barrett, Jessica Barrett, Kelly Barrett, Louise Bates, Alexander Batten, Kirsten Beadon, Emily Beales, Rebecca Beckley, Sandra Belij-Rammerstorfer, Jonathan Bell, Duncan Bellamy, Sue Belton, Adam Berg, Laura Bermejo, Eleanor Berrie, Lisa Berry, Daniella Berzenyi, Amy Beveridge, Kevin R. Bewley, Inderjeet Bharaj, Sutika Bhikha, Asad E. Bhorat, Zaheda E. Bhorat, Else Margreet Bijker, Sarah Birch, Gurpreet Birch, Kathryn Birchall, Adam Bird, Olivia Bird, Karen Bisnauthsing, Mustapha Bittaye, Luke Blackwell, Rachel Blacow, Heather Bletchly, Caitlin L. Blundell, Susannah R. Blundell, Pritesh Bodalia, Emma Bolam, Elena Boland, Daan Bormans, Nicola Borthwick, Francesca Bowring, Amy Boyd, Penny Bradley, Tanja Brenner, Alice Bridges-Webb, Phillip Brown, Claire Brown, Charlie Brown-O'Sullivan, Scott Bruce, Emily Brunt, William Budd, Yusuf A. Bulbulia, Melanie Bull, Jamie Burbage, Aileen Burn, Karen R. Buttigieg, Nicholas Byard, Ingrid Cabrera Puig, Anna Calvert, Susana Camara, Michelangelo Cao, Federica Cappuccini, Rita Cardona, João R. Cardoso, Melanie Carr, Miles W. Carroll, Andrew Carson-Stevens, Yasmin de M. Carvalho, Helen R. Casey, Paul Cashen, Thais R.Y. Castro, Lucia Carratala Castro, Katrina Cathie, Ana Cavey, José Cerbino-Neto, Luiz Fernando F. Cezar, Jim Chadwick, Chanice Chanice, David Chapman, Sue Charlton, Katerina S. Cheliotis, Irina Chelysheva, Oliver Chester, Emily Chiplin, Sunder Chita, Jee-Sun Cho, Liliana Cifuentes, Elizabeth Clark, Matthew Clark, Rachel Colin-Jones, Sarah L.K. Collins, Hayley Colton, Christopher P. Conlon, Sean Connarty, Naomi Coombes, Cushla Cooper, Rachel Cooper, Lynne Cornelissen, Tumena Corrah, Catherine A. Cosgrove, Fernanda Barroso Costa, Tony Cox, Wendy E.M. Crocker, Sarah Crosbie, Dan Cullen, Debora R.M.F. Cunha, Christina J. Cunningham, Fiona C. Cuthbertson, Daniel Marinho da Costa, Suzete N. Farias Da Guarda, Larissa P. da Silva, Antonio Carlos da Silva Moraes, Brad E. Damratoski, Zsofia Danos, Maria T.D.C. Dantas, Mehreen S. Datoo, Chandrabali Datta, Malika Davids, Sarah L. Davies, Kelly Davies, Hannah Davies, Sophie Davies, Judith Davies, Elizabeth J. Davis, John Davis, José A.M. de Carvalho, Jeanne De Jager, Sergio de Jesus Jnr, Lis Moreno De Oliveira Kalid, David Dearlove, Tesfaye Demissie, Amisha Desai, Stefania Di Marco, Claudio Di Maso, Tanya Dinesh, Claire Docksey, Tao Dong, Francesca R. Donnellan, Tannyth Gomes Dos Santos, Thainá G. Dos Santos, Erika Pachecho Dos Santos, Naomi Douglas, Charlotte Downing, Jonathan Drake, Rachael Drake-Brockman, Ruth Drury, Joan Du Plessis, Susanna J. Dunachie, Andrew Duncan, Nicholas J.W. Easom, Mandy Edwards, Nick J. Edwards, Frances Edwards, Omar M. El Muhanna, Sean C. Elias, Branwen Ellison-Handley, Michael J. Elmore, Marcus Rex English, Alisgair Esmail, Yakub Moosa Essack, Mutjaba Farooq, Sofiya Fedosyuk, Sally Felle, Susie Ferguson, Carla Ferreira Da Silva, Samantha Field, Richard Fisher, James Fletcher, Hazel Fofie, Henry Fok, Karen J. Ford, Ross Fothergill, Jamie Fowler, Pedro H.A. Fraiman, Emma Francis, Marilia M. Franco, John Frater, Marilúcia S.M. Freire, Samantha H. Fry, Sabrina Fudge, Renato Furlan Filho, Julie Furze, Michelle Fuskova, Pablo Galian-Rubio, Harriet Garlant, Madita Gavrila, Karyna A. Gibbons, Ciaran Gilbride, Hardeep Gill, Kerry Godwin, Karishma Gokani, Maria Luisa Freire Gonçalves, Isabela G.S. Gonzalez, Jack Goodall, Jayne Goodwin, Amina Goondiwala, Katherine Gordon-Quayle, Giacomo Gorini, Alvaro Goyanna, Janet Grab, Lara Gracie, Justin Green, Nicola Greenwood, Johann Greffrath, Marisa M. Groenewald, Anishka Gunawardene, Gaurav Gupta, Mark Hackett, Bassam Hallis, Mainga Hamaluba, Elizabeth Hamilton, Joseph Hamlyn, Daniel Hammersley, Aidan T. Hanrath, Brama Hanumunthadu, Stephanie A. Harris, Clair Harris, Thomas D. Harrison, Daisy Harrison, Tara A. Harris-Wright, Thomas C. Hart, Birgit Hartnell, John Haughney, Sophia Hawkins, Laís Y.M. Hayano, Ian Head, Paul T. Heath, John Aaron Henry, Macarena Hermosin Herrera, David B. Hettle, Cristhiane Higa, Jennifer Hill, Gina Hodges, Susanne Hodgson, Elizea Horne, Mimi M. Hou, Catherine F. Houlihan, Elizabeth Howe, Nicola Howell, Jonathan Humphreys, Holly E. Humphries, Katrina Hurley, Claire Huson, Catherine Hyams, Angela Hyder-Wright, Sabina Ikram, Alka Ishwarbhai, Poppy Iveson, Vidyashankara Iyer, Frederic Jackson, Susan Jackson, Shameem Jaumdally, Helen Jeffers, Natasha Jesudason, Carina Jones, Christopher Jones, Kathryn Jones, Elizabeth Jones, Marianna Rocha Jorge, Amar Joshi, Eduardo A.M.S. Júnior, Reshma Kailath, Faeeza Kana, Arnab Kar, Konstantinos Karampatsas, Mwila Kasanyinga, Linda Kay, Jade Keen, Johanna Kellett Wright, Elizabeth J. Kelly, Debbie Kelly, Dearbhla M. Kelly, Sarah Kelly, David Kerr, Liaquat Khan, Baktash Khozoee, Ankush Khurana, Sarah Kidd, Annabel Killen, Jasmin Kinch, Patrick Kinch, Lloyd D.W. King, Thomas B. King, Lucy Kingham, Paul Klenerman, Diana M. Kluczna, Francesca Knapper, Julian C. Knight, Daniel Knott, Stanislava Koleva, Pedro M. Lages, Matilda Lang, Gail Lang, Colin W. Larkworthy, Jessica P.J. Larwood, Rebecca Law, Alison M. Lawrie, Erica M. Lazarus, Amanda Leach, Emily A. Lees, Alice Lelliott, Nana-Marie Lemm, Alvaro Edson Ramos Lessa, Stephanie Leung, Yuanyuan Li, Amelia M. Lias, Konstantinos Liatsikos, Aline Linder, Samuel Lipworth, Shuchang Liu, Xinxue Liu, Adam Lloyd, Stephanie Lloyd, Lisa Loew, Raquel Lopez Ramon, Leandro Bonecker Lora, Kleber Giovanni Luz, Jonathan C. MacDonald, Gordon MacGregor, Meera Madhavan, David O. Mainwaring, Edson Makambwa, Rebecca Makinson, Mookho Malahleha, Ross Malamatsho, Garry Mallett, Nicola Manning, Kushal Mansatta, Takalani Maoko, Spyridoula Marinou, Emma Marlow, Gabriela N. Marques, Paula Marriott, Richard P. Marshall, Julia L. Marshall, Masebole Masenya, Mduduzi Masilela, Shauna K. Masters, Moncy Mathew, Hosea Matlebjane, Kedidimetse Matshidiso, Olga Mazur, Andrea Mazzella, Hugh McCaughan, Joanne McEwan, Joanna McGlashan, Lorna McInroy, Nicky McRobert, Steve McSwiggan, Clare Megson, Savviz Mehdipour, Wilma Meijs, Renata N.Õ. Mendonça, Alexander J. Mentzer, Ana Carolina F. Mesquita, Patricia Miralhes, Neginsadat Mirtorabi, Celia Mitton, Sibusiso Mnyakeni, Fiona Moghaddas, Kgaogelo Molapo, Mapule Moloi, Maria Moore, Marni Moran, Ella Morey, Róisín Morgans, Susan J. Morris, Sheila Morris, Hazel Morrison, Franca Morselli, Gertraud Morshead, Richard Morter, Lynelle Mottay, Andrew Moultrie, Nathifa Moyo, Mushiya Mpelembue, Sibekezelo Msomi, Yvonne Mugodi, Ekta Mukhopadhyay, Jilly Muller, Alasdair Munro, Sarah Murphy, Philomena Mweu, Christopher Myerscough, Gurudutt Naik, Kush Naker, Eleni Nastouli, Bongani Ndlovu, Elissavet Nikolaou, Cecilia Njenga, Helena C. Noal, Andrés Noé, Gabrielle Novaes, Fay L. Nugent, Géssika Lanzillo A. Nunes, Katie O'Brien, Daniel O'Connor, Suzette Oelofse, Blanche Oguti, Victoria Olchawski, Neil J. Oldfield, Marianne G. Oliveira, Catarina Oliveira, Isabelle Silva Queiroz Oliveira, Aylin Oommen-Jose, Angela Oosthuizen, Paula O'Reilly, Peter J. O'Reilly, Piper Osborne, David R.J. Owen, Lydia Owen, Daniel Owens, Nelly Owino, Mihaela Pacurar, Brenda V.B. Paiva, Edna M.F. Palhares, Susan Palmer, Helena M. R.T. Parracho, Karen Parsons, Dipak Patel, Bhumika Patel, Faeezah Patel, Maia Patrick-Smith, Ruth O. Payne, Yanchun Peng, Elizabeth J. Penn, Anna Pennington, Marco Polo Peralta Alvarez, Bruno Pereira Pereira Stuchi, Ana Luiza Perez, Tanaraj Perinpanathan, James Perring, Rubeshan Perumal, Sahir Yusuf Petkar, Tricia Philip, Jennifer Phillips, Mary Kgomotso Phohu, Lorinda Pickup, Sonja Pieterse, Jessica Morgana Pinheiro, Jo Piper, Dimitra Pipini, Mary Plank, Sinéad Plant, Samuel Pollard, Jennifer Pooley, Anil Pooran, Ian Poulton, Claire Powers, Fernando B. Presa, David A. Price, Vivien Price, Marcelo R. Primeira, Pamela C. Proud, Samuel Provstgaard-Morys, Sophie Pueschel, David Pulido, Sheena Quaid, Ria Rabara, Kajal Radia, Durga Rajapaska, Thurkka Rajeswaran, Leonardo Ramos, Alberto San Francisco Ramos, Fernando Ramos Lopez, Tommy Rampling, Jade Rand, Helen Ratcliffe, Tom Rawlinson, David Rea, Byron Rees, Mila Resuello-Dauti, Emilia Reyes Pabon, Sarah Rhead, Tawassal Riaz, Marivic Ricamara, Alexander Richards, Alex Richter, Neil Ritchie, Adam J. Ritchie, Alexander J. Robbins, Hannah Roberts, Ryan E. Robinson, Sophie Roche, Christine Rollier, Louisa Rose, Amy L. Ross Russell, Lindie Rossouw, Simon Royal, Indra Rudiansyah, Kim Ryalls, Charlotte Sabine, Stephen Saich, Jessica C. Sale, Ahmed M. Salman, Natalia Salvador, Stephannie Salvador, Milla Dias Sampaio, Annette D. Samson, Amada Sanchez-Gonzalez, Helen Sanders, Katherine Sanders, Erika Santos, Mayara F.S. Santos Guerra, Iman Satti, Jack E. Saunders, Caroline Saunders, Aakifah Bibi Arif Sayed, Ina Schim van der Loeff, Annina B. Schmid, Ella Schofield, Gavin R. Screaton, Samiullah Seddiqi, Rameswara R. Segireddy, Roberta Senger, Sonia Serrano, Imam Shaik, Hannah R. Sharpe, Katherine Sharrocks, Robert Shaw, Adam Shea, Emma Sheehan, Amy Shepherd, Farah Shiham, Sarah E. Silk, Laura Silva-Reyes, Lidiana B. T.D. Silveira, Mariana B.V. Silveira, Nisha Singh, Jaisi Sinha, Donal T. Skelly, Daniel C. Smith, Nick Smith, Holly E. Smith, David J. Smith, Catherine C. Smith, Airanuédida S. Soares, Carla Solórzano, Guilherme L. Sorio, Kim Sorley, Tiffany Sosa-Rodriguez, Cinthia M.C.D.L. Souza, Bruno S.D.F. Souza, Alessandra R. Souza, Thamyres Souza Lopez, Luciana Sowole, Alexandra J. Spencer, Louise Spoors, Lizzie Stafford, Imogen Stamford, Ricardo Stein, Lisa Stockdale, Lisa V. Stockwell, Louise H. Strickland, Arabella Stuart, Ann Sturdy, Natalina Sutton, Anna Szigeti, Abdessamad Tahiri-Alaoui, Rachel Tanner, Carol Taoushanis, Alexander W. Tarr, Richard Tarrant, Keja Taylor, Ursula Taylor, Iona Jennifer Taylor, Justin Taylor, Rebecca te Water Naude, Kate Templeton, Yrene Themistocleous, Andreas Themistocleous, Merin Thomas, Kelly Thomas, Tonia M. Thomas, Asha Thombrayil, Julia Thompson, Fawziyah Thompson, Ameeka Thompson, Amber Thompson, Kevin Thompson, Viv Thornton-Jones, Larissa H.S. Thotusi, Patrick J. Tighe, Lygia Accioly Tinoco, Gerlynn Ferreras Tiongson, Bonolo Tladinyane, Michele Tomasicchio, Adriana Tomic, Susan Tonks, James Towner, Nguyen Tran, Julia A. Tree, Gerry Trillana, Charlotte Trinham, Rose Trivett, Adam Truby, Betty Lebogang Tsheko, Philippa Tubb, Aadil Turabi, Richard Turner, Cheryl Turner, Nicola Turner, Bhavya Tyagi, Marta Ulaszewska, Benjamin R. Underwood, Samual van Eck, Rachel Varughese, Dennis Verbart, Marije K. Verheul, Iason Vichos, Taiane A. Vieira, Gemma Walker, Laura Walker, Matthew E. Wand, Theresa Wardell, George M. Warimwe, Sarah C. Warren, Bridget Watkins, Marion E.E. Watson, Ekaterina Watson, Stewart Webb, Angela Webster, Jessica Welch, Zoe Wellbelove, Jeanette H. Wells, Alison J. West, Beth White, Caroline White, Rachel White, Paul Williams, Rachel L. Williams, Silvia Willingham, Rebecca Winslow, Danielle Woods, Mark Woodyer, Andrew T. Worth, Danny Wright, Marzena Wroblewska, Andy Yao, Yee Ting Nicole Yim, Marina Bauer Zambrano, Rafael Leal Zimmer, Dalila Zizi, Peter Zuidewind

**Affiliations:** a Oxford Vaccine Group, Department of Paediatrics, University of Oxford, Oxford, UK; b Jenner Institute, Nuffield Department of Medicine, University of Oxford, Oxford, UK; c Institute of Global Health, University of Siena, Siena, Italy; d Department of Paediatrics, University of Oxford, Oxford, UK; e Clinical BioManufacturing Facility, University of Oxford, Oxford, UK; f South African Medical Research Council Vaccines and Infectious Diseases Analytics Research Unit, Faculty of Health Sciences, University of the Witwatersrand, Johannesburg, South Africa; g Department of Science and Innovation/National Research Foundation South African Research Chair Initiative in Vaccine Preventable Diseases Unit, University of the Witwatersrand, Johannesburg, South Africa; h Wits Reproductive Health and HIV Institute, Faculty of Health Sciences, University of the Witwatersrand, Johannesburg, South Africa; i Perinatal HIV Research Unit, Faculty of Health Sciences, University of the Witwatersrand, Johannesburg, South Africa; j Department of Pediatrics, Universidade Federal de São Paulo, São Paulo, Brazil; k AstraZeneca BioPharmaceuticals, Cambridge, UK; l Family Centre for Research with Ubuntu, Department of Paediatrics, University of Stellenbosch, Cape Town, South Africa; m Soweto Clinical Trials Centre, Soweto, South Africa; n Department of Clinical Sciences, Liverpool School of Tropical Medicine and Liverpool University Hospitals NHS Foundation Trust, Liverpool, UK; o Department of Infection, Immunity and Cardiovascular Disease, University of Sheffield, Sheffield, UK; p Department of Infection and Tropical Medicine, Sheffield Teaching Hospitals NHS Foundation Trust, Sheffield, UK; q Escola Bahiana de Medicina e Saúde Pública, Salvador, Braziland Hospital São Rafael, Salvador, Brazil; r Instituto D’Or, Salvador, Brazil; s Division of Pulmonology, Groote Schuur Hospital and the University of Cape Town, Cape Town, South Africa; t Faculty of Infectious and Tropical Diseases, Department of Immunology and Infection, London School of Hygiene & Tropical Medicine, London, UK; u Department of Infection and Tropical Medicine, Newcastle upon Tyne Hospitals NHS Foundation Trust, Newcastle upon Tyne, UK; v Translational and Clinical Research Institute, Immunity and Inflammation Theme, Newcastle University, Newcastle upon Tyne, UK; w NIHR Southampton Clinical Research Facility and Biomedical Research Centre, University Hospital Southampton NHS Foundation Trust, University of Southampton, Southampton, UK; x Faculty of Medicine and Institute for Life Sciences, University of Southampton, Southampton, UK; y School of Population Health Sciences, University of Bristol and University Hospitals Bristol and Weston NHS Foundation Trust, UK; z Department of Infection, Guy's and St Thomas’ NHS Foundation Trust, St Thomas’ Hospital, London, UK; aa MRC Clinical Trials Unit, University College London, London, UK; ab NIHR/Wellcome Trust Clinical Research Facility, University Hospitals Birmingham NHS Foundation Trust, Birmingham, UK; ac St George's Vaccine Institute, St George's, University of London, London, UK; ad Severn Pathology, North Bristol NHS Trust, Bristol, UK; ae NIHR UCLH Clinical Research Facility and NIHR UCLH Biomedical Research Centre, London, UK; af Department of Infection, Hull University Teaching Hospitals NHS Trust, Hull, UK; ag London Northwest University Healthcare, Harrow, UK; ah Setshaba Research Centre, Pretoria, South Africa; ai Universidade Federal do Rio Grande do Norte, Natal, Brazil; aj Hospital Quinta D’Or, Rede D’Or, Rio De Janeiro, Brazil; ak NIHR Imperial Clinical Research Facility and NIHR Imperial Biomedical Research Centre, London, UK; al College of Medical, Veterinary & Life Sciences, Glasgow Dental Hospital & School, University of Glasgow, Glasgow, UK; am Infectious Diseases Service, Hospital de Clinicas de Porto Alegre, Universidade Federal do Rio Grande do Sul, Porto Alegre, Brazil; an Clinical Research Unit, Department of Clinical Medicine, Universidade Federal de Santa Maria, Santa Maria, Brazil; ao Clinical Infection Research Group, Regional Infectious Diseases Unit, Western General Hospital, Edinburgh, UK; ap MRC-University of Glasgow Centre for Virus Research & Department of Infectious Diseases, Queen Elizabeth University Hospital, Glasgow, UK; aq Department of Medicine, University of Cambridge, UK; ar Cambridge University Hospitals NHS Foundation Trust, Cambridge, UK; as Heart Lung Research Institute, Dept of Medicine, University of Cambridge and NIHR Cambridge Clinical Research Facility, Cambridge University Hospital and Royal Papworth NHS Foundation Trusts, Cambridge, UK; at University of Nottingham and Nottingham University Hospitals NHS Trust, Nottingham, UK; au Public Health Wales, Cardiff, Wales; av Aneurin Bevan University Health Board, Newport, Wales

## Abstract

**Background:**

The ChAdOx1 nCoV-19 (AZD1222) vaccine has been approved for emergency use by the UK regulatory authority, Medicines and Healthcare products Regulatory Agency, with a regimen of two standard doses given with an interval of 4–12 weeks. The planned roll-out in the UK will involve vaccinating people in high-risk categories with their first dose immediately, and delivering the second dose 12 weeks later. Here, we provide both a further prespecified pooled analysis of trials of ChAdOx1 nCoV-19 and exploratory analyses of the impact on immunogenicity and efficacy of extending the interval between priming and booster doses. In addition, we show the immunogenicity and protection afforded by the first dose, before a booster dose has been offered.

**Methods:**

We present data from three single-blind randomised controlled trials—one phase 1/2 study in the UK (COV001), one phase 2/3 study in the UK (COV002), and a phase 3 study in Brazil (COV003)—and one double-blind phase 1/2 study in South Africa (COV005). As previously described, individuals 18 years and older were randomly assigned 1:1 to receive two standard doses of ChAdOx1 nCoV-19 (5 × 10^10^ viral particles) or a control vaccine or saline placebo. In the UK trial, a subset of participants received a lower dose (2·2 × 10^10^ viral particles) of the ChAdOx1 nCoV-19 for the first dose. The primary outcome was virologically confirmed symptomatic COVID-19 disease, defined as a nucleic acid amplification test (NAAT)-positive swab combined with at least one qualifying symptom (fever ≥37·8°C, cough, shortness of breath, or anosmia or ageusia) more than 14 days after the second dose. Secondary efficacy analyses included cases occuring at least 22 days after the first dose. Antibody responses measured by immunoassay and by pseudovirus neutralisation were exploratory outcomes. All cases of COVID-19 with a NAAT-positive swab were adjudicated for inclusion in the analysis by a masked independent endpoint review committee. The primary analysis included all participants who were SARS-CoV-2 N protein seronegative at baseline, had had at least 14 days of follow-up after the second dose, and had no evidence of previous SARS-CoV-2 infection from NAAT swabs. Safety was assessed in all participants who received at least one dose. The four trials are registered at ISRCTN89951424 (COV003) and ClinicalTrials.gov, NCT04324606 (COV001), NCT04400838 (COV002), and NCT04444674 (COV005).

**Findings:**

Between April 23 and Dec 6, 2020, 24 422 participants were recruited and vaccinated across the four studies, of whom 17 178 were included in the primary analysis (8597 receiving ChAdOx1 nCoV-19 and 8581 receiving control vaccine). The data cutoff for these analyses was Dec 7, 2020. 332 NAAT-positive infections met the primary endpoint of symptomatic infection more than 14 days after the second dose. Overall vaccine efficacy more than 14 days after the second dose was 66·7% (95% CI 57·4–74·0), with 84 (1·0%) cases in the 8597 participants in the ChAdOx1 nCoV-19 group and 248 (2·9%) in the 8581 participants in the control group. There were no hospital admissions for COVID-19 in the ChAdOx1 nCoV-19 group after the initial 21-day exclusion period, and 15 in the control group. 108 (0·9%) of 12 282 participants in the ChAdOx1 nCoV-19 group and 127 (1·1%) of 11 962 participants in the control group had serious adverse events. There were seven deaths considered unrelated to vaccination (two in the ChAdOx1 nCov-19 group and five in the control group), including one COVID-19-related death in one participant in the control group. Exploratory analyses showed that vaccine efficacy after a single standard dose of vaccine from day 22 to day 90 after vaccination was 76·0% (59·3–85·9). Our modelling analysis indicated that protection did not wane during this initial 3-month period. Similarly, antibody levels were maintained during this period with minimal waning by day 90 (geometric mean ratio [GMR] 0·66 [95% CI 0·59–0·74]). In the participants who received two standard doses, after the second dose, efficacy was higher in those with a longer prime-boost interval (vaccine efficacy 81·3% [95% CI 60·3–91·2] at ≥12 weeks) than in those with a short interval (vaccine efficacy 55·1% [33·0–69·9] at <6 weeks). These observations are supported by immunogenicity data that showed binding antibody responses more than two-fold higher after an interval of 12 or more weeks compared with an interval of less than 6 weeks in those who were aged 18–55 years (GMR 2·32 [2·01–2·68]).

**Interpretation:**

The results of this primary analysis of two doses of ChAdOx1 nCoV-19 were consistent with those seen in the interim analysis of the trials and confirm that the vaccine is efficacious, with results varying by dose interval in exploratory analyses. A 3-month dose interval might have advantages over a programme with a short dose interval for roll-out of a pandemic vaccine to protect the largest number of individuals in the population as early as possible when supplies are scarce, while also improving protection after receiving a second dose.

**Funding:**

UK Research and Innovation, National Institutes of Health Research (NIHR), The Coalition for Epidemic Preparedness Innovations, the Bill & Melinda Gates Foundation, the Lemann Foundation, Rede D’Or, the Brava and Telles Foundation, NIHR Oxford Biomedical Research Centre, Thames Valley and South Midland's NIHR Clinical Research Network, and AstraZeneca.

## Introduction

The widespread morbidity and mortality associated with the 2020 COVID-19 pandemic precipitated the most extensive and rapid global vaccine development programme in history,^1^ culminating in the development of several vaccines reaching phase 3 efficacy milestones and receiving emergency use authorisation by the end of that year.^2^, ^3^, ^4^ Widespread vaccination programmes have commenced in several countries as new vaccines are licensed for emergency use by regulators in each setting, with a focus primarily on high-risk groups such as the elderly, those with comorbidities, or front-line workers.

Vaccine supply is likely to be scarce, at least initially, and so policy makers must decide how best to deliver available doses to achieve greatest public health benefit, and different approaches have been taken in different settings. In the UK, second doses of both available vaccines (a viral vector and mRNA vaccine) are being delivered with an interval of up to 12 weeks,^5^, ^6^ and this regimen is also being considered by several other countries.^7^, ^8^ By contrast, WHO has recently recommended a maximum 6 week interval between the two doses of the same mRNA vaccine.^9^

The ChAdOx1 nCoV-19 vaccine (AZD1222) is a chimpanzee adenoviral vectored vaccine with full length SARS-CoV-2 spike insert, developed at the University of Oxford (Oxford, UK). The safety and immunogenicity of the vaccine were assessed in four randomised controlled trials in the UK, Brazil, and South Africa, and results in cohorts of healthy adults and in adults aged 70 years or older have been published.^4^, ^10^, ^11^, ^12^, ^13^ Efficacy of two doses of the vaccine in the interim analysis of 131 cases (data cutoff Nov 4, 2020), which pooled data from Brazil and the UK, was 70·4% (95·8% CI 54·8–80·6) overall.^4^ ChAdOx1 nCoV-19 was authorised for emergency use in the UK on Dec 30, 2020,^14^ on the basis of the interim analysis data,^4^ based on a regimen of two standard doses administered 4–12 weeks apart for adults aged 18 years and older, and has since been authorised for use in many other countries.

**Research in context**

**Evidence before this study**

The ChAdOx1 nCoV-19 (AZD1222) vaccine was approved for emergency use authorisation in the UK on the basis of interim efficacy results from 131 cases of primary symptomatic COVID-19, with efficacy based on two of the four trials of the vaccine. The planned roll-out of the vaccine in the UK involves the administration of two doses 12 weeks apart, a policy that has received substantial comment.

**Added value of this study**

This report provides updated primary efficacy results after a further month of data collection. The interim report included 131 cases of primary symptomatic COVID-19. The latest results with additional follow-up include 332 cases of primary symptomatic COVID-19. Efficacy estimates now include data from all four studies of the vaccine from three countries, whereas the interim analysis included only two studies in efficacy assessments because of the small number of cases in the smaller studies. In addition to the primary efficacy assessment, post-hoc exploratory analyses have been added, including a breakdown of efficacy by prime-boost interval, and the efficacy of a single dose of vaccine.

**Implications of all the available evidence**

The primary analysis supports the findings reported in the interim analysis that the vaccine is efficacious and safe. Exploratory analyses show that higher vaccine efficacy is obtained with a longer prime-boost interval, and that a single dose of vaccine is efficacious in the first 90 days, providing further evidence for current policy.

The University of Oxford-sponsored studies were initially planned as single-dose studies but were amended to incorporate a second dose after review of the phase 1 immunogenicity data, which showed a substantial increase in neutralising antibody with a second dose of vaccine.^12^ After initially providing consent to participate in a single-dose study, some participants chose not to receive the second dose, providing a self-selected cohort of single-dose recipients. Additionally, because of the time required to manufacture the second dose, there were delays in administration of the second dose for a large number of trial participants who received the two-dose schedule. These two situations provide an opportunity to explore the immunogenicity and efficacy of a single dose of vaccine, and the effect of an extended interval before delivery of the second dose. In addition, data from an additional month of follow-up are now available for inclusion in the analysis, providing greater precision in estimates because of the larger number of cases for analysis in comparison with the previous report.^4^

## Methods

### Study design and participants

Data from three single-blind randomised controlled trials, one phase 1/2 study in the UK (COV001), one phase 2/3 study in the UK (COV002), and a phase 3 study in Brazil (COV003), and one double-blind phase 1/2 study in South Africa (COV005) are included in this primary analysis because all four trials now meet the required criteria for inclusion of having at least five primary outcome cases. Full descriptions of the methods as well as safety, immunogenicity, and interim efficacy analyses of the four studies have been previously published in detail, including full study protocols.^4^, ^12^, ^13^

COV001 (UK) enrolled healthy adults aged 18–55 years. COV002 (UK) and COV003 (Brazil) enrolled adults aged 18 years and older, with a focus on recruitment of health-care workers and others at increased exposure to SARS-CoV-2 infection. COV005 (South Africa) enrolled adults aged 18–65 years.

In the UK, the COV001 and COV002 studies were approved by the South Central Berkshire Research Ethics Committee (COV001 reference 20/SC/0145, March 23, 2020; and COV002 reference 20/SC/0179; conditional approval April 8, full approval April 19, 2020). The COV003 study was approved by the Oxford Tropical Research Ethics Committee (OxTREC; reference 36–20, June 12, 2020) and by the Comissão Nacional de Ética em Pesquisa (June 3, 2020). The COV005 study was approved by OxTREC (reference 35-20, June 5, 2020), the University of Witwatersand Human Research Ethics committee (reference 200501, May 21, 2020), and the South African Health Products Regulatory Authority (reference 20200407, June 1, 2020).

### Randomisation and masking

Briefly, participants in efficacy cohorts from the four trials were randomly assigned 1:1 with full allocation concealment to receive either ChAdOx1 nCoV-19 vaccine or a control product (MenACWY in the UK, MenACWY prime and saline boost in Brazil, and saline only in South Africa). One group of participants in the COV002 study in the UK received a low dose as their first dose followed by a standard dose, as discussed previously.^4^ Other participants received two standard doses.

### Procedures

Procedures have been described in full previously.^4^, ^12^, ^13^ At baseline, eligibility and medical history was assessed and informed consent was taken from all participants. A baseline serum sample was taken to assess SARS-CoV-2 serostatus.

In all studies, participants were asked to contact the study site if they had symptoms of COVID-19 and were then invited to attend for clinical review and a swab. Additionally, in the UK, asymptomatic infections were measured by means of weekly self-administered nose and throat swabs using kits provided by the Department of Health and Social Care as previously described.^4^ Those who tested positive on a self-swab were not specifically contacted by the study site and they are classed as having unknown symptoms in the analysis unless specific information was obtained (eg, through participants calling their study site) that the participant was asymptomatic, or unless the participant had additionally reported symptoms and could be classified as symptomatic.

### Outcomes

The primary outcome was virologically confirmed symptomatic COVID-19 disease, defined as a nucleic acid amplification test (NAAT)-positive swab combined with at least one qualifying symptom (fever ≥37·8°C, cough, shortness of breath, anosmia, or ageusia). The primary analysis was of cases occurring more than 14 days after the second dose, with a prespecified secondary analysis of cases occurring more than 21 days after the first dose.

A secondary analysis of any NAAT-positive case included a combination of primary symptomatic cases, non-primary symptomatic cases (eg, those who had other symptoms not meeting the primary definition such as nausea or diarrhoea), asymptomatic cases, and those with unknown symptoms.

### Statistical analysis

The study was powered to include an α-adjusted interim analysis triggered when at least 53 cases had accrued in participants who had received two standard-dose vaccines. This analysis was statistically significant and subsequent efficacy results are considered supportive of that analysis, with no further adjustment of α.

For the primary analysis, which we present here updated with additional cases from an extra month of follow-up, participants enrolled in efficacy cohorts were included in the analysis according to the vaccine received. Events were included that occurred more than 14 days after the second dose, in participants who were seronegative to SARS-CoV-2 N protein at baseline and had at least 14 days of follow-up after the second dose and no evidence of SARS-CoV-2 infection from NAAT swabs before day 14. Vaccine efficacy was calculated as 1–the adjusted relative risk (ChAdOx1 nCoV-19 *vs* control groups) computed using a robust Poisson regression model. The model contained terms for study, treatment group, and age group at randomisation. The logarithm of the period at risk was used as an offset variable in the model to adjust for volunteers having different follow-up times during which the events occurred. Cumulative incidence of primary symptomatic COVID-19 is presented using the Kaplan-Meier method.

We present additional exploratory analyses of single-dose efficacy, which have been added at the request of regulators and policy makers. These are considered as supportive analyses to the previously published interim efficacy analysis and were not prespecified. The effect of the timing of the second dose is explored in more detail.

For the analysis of single-dose efficacy, randomised participants enrolled in efficacy cohorts were included in the analysis according to the vaccine they received as their first dose. Events were included if they occurred more than 21 days after the first dose. Participants were excluded if they had a NAAT-positive swab in the first 21 days after the first dose or had fewer than 22 days of follow-up. Participants who received a second dose were censored in the analysis at the time of their booster dose. Participants who did not receive a second dose are censored in the analysis at the data cutoff date.

For exploratory analysis, the persistence of anti-spike IgG responses after a single dose was measured in the UK by standardised ELISA. Decay of antibody over time was modelled for low-dose and standard-dose recipients using a linear model of log-transformed antibody values. A non-linear generalised additive model was also used to assess the shape of the decay curve to establish whether linear modelling was appropriate. Both models gave similar outputs.

Baseline serum samples were measured for nucleocapsid reactivity with the Roche Elecsys Anti-SARS-CoV-2 serology test (PPD Central Laboratories, Zaventem, Belgium and Highland Heights, KY, USA) and a multiplexed immunoassay (3-plex ECL based assay on the MSD platform, PPD Vaccines, Richmond, VA, USA) was used to measure the spike-specific response to ChAdOx1 nCoV-19 vaccination. Antibody neutralisation was measured with lentivirus-based pseudovirus particles expressing the SARS-CoV-2 spike protein as described.^12^

For exploratory analyses of the effect of varying the timing of the second dose of vaccine, we fit separate efficacy models, using unadjusted log-binomial models, for each 20-day window starting with a window of 26–46 days (midpoint for plot 36 days) and increasing by 1 day for each model. Participants who received their second dose within the window were included in that model. Vaccine efficacy for each window was plotted with 95% CIs. Unadjusted models were used to achieve convergence across every model consistently and to remove bias from the potentially different effect of variation in the distribution of adjustment variables in different models. Participants were not randomly assigned to their dosing interval and these exploratory analyses should be interpreted with caution because it is not possible to exclude the possibility that any apparent trend is due to measured or unmeasured confounding factors.

To explore the potential for waning of efficacy after the first dose before a booster dose was received, a similar approach was taken with separate efficacy models fitted to 21-day windows of the time from vaccination. Cases occurring outside the windows were censored.

Potential differences in population baseline characteristics between those who received a second dose of vaccine and those who did not are explored descriptively, with comparisons made between groups using χ^2^ tests, Wilcoxon rank-sum tests, or Cochran-Armitage tests as appropriate.

Safety was assessed in all participants who received at least one dose. Safety data were reviewed on an ongoing basis by the independent data monitoring safety board. All endpoints were adjudicated for inclusion in the analysis by an independent masked endpoint review committee.

Data analysis was done using R, version 3.6.1 or later. Robust Poisson models were fitted using “proc genmod” function in SAS, version 9.4. The four trials are registered at ISRCTN89951424 (COV003) and ClinicalTrials.gov, NCT04324606 (COV001), NCT04400838 (COV002), and NCT04444674 (COV005).

### Role of the funding source

AstraZeneca reviewed the data from the study and the final manuscript before submission, but the academic authors retained editorial control. All other funders of the study had no role in the study design, data collection, data analysis, data interpretation, or writing of the report.

## Results

Between April 23 and Dec 6, 2020, 24 422 participants were recruited and vaccinated across the four studies, of whom 17 178 were included in this primary efficacy analysis (8597 receiving ChAdOx1 nCoV-19 and 8581 receiving control vaccine). 8948 were from the UK trial, 6753 from the Brazil trial, and 1477 from the South Africa trial (appendix p 2). Here, we provide safety data on 100 958 person-months of follow-up after first dose and 49 945 person-months of follow-up after two doses. Baseline characteristics were similar for vaccine and control groups (appendix p 3). Duration of follow-up varied by prime-boost interval (appendix p 4). The day for data cutoff for cases to be included in this report was Dec 7, 2020.

There were 332 cases of primary symptomatic COVID-19 occurring more than 14 days after a booster dose, 84 (1·0%) in the 8597 participants in the ChAdOx1 nCoV-19 group and 248 (2·9%) in the 8581 participants in the control group, with overall efficacy of 66·7% (95% CI 57·4–74·0; table 1). In the participants who received two standard doses, 74 (1·0%) cases occurred in the 7201 participants in the ChAdOx1 nCoV-19 group and 197 (2·7%) in the 7179 in the control group, with vaccine efficacy of 63·1% (51·8–71·7). 61 cases were recorded in the participants who received a low dose plus standard dose, ten (0·7%) of 1396 participants in the ChAdOx1 nCoV-19 group and 51 (3·6%) of 1402 in the control group, with vaccine efficacy of 80·7% (62·1–90·2).

**Table 1:** Efficacy of ChAdOx1 nCoV-19 more than 14 days after a second dose

			**Total cases**	**ChAdOx1 nCoV-19**	**Control**	Vaccine efficacy (95% CI)*
**Prespecified analyses**						
Cases more than 14 days after second dose						
	Primary symptomatic COVID-19		332	84/8597 (1·0%)	248/8581 (2·9%)	66·7% (57·4 to 74·0)
		Two standard doses	271	74/7201 (1·0%)	197/7179 (2·7%)	63·1% (51·8 to 71·7)†
		Low dose plus standard dose	61	10/1396 (0·7%)	51/1402 (3·6%)	80·7% (62·1 to 90·2)
	Asymptomatic or unknown infection (COV002 UK only)		130	57/4071 (1·4%)	73/4136 (1·8%)	22·2% (−9·9 to 45·0)
		Two standard doses	83	41/2692 (1·5%)	42/2751 (1·5%)	2·0% (−50·7 to 36·2)
		Low dose plus standard dose	47	16/1379 (1·2%)	31/1385 (2·2%)	49·3% (7·4 to 72·2)
	Any NAAT positive		507	161/8597 (1·9%)	346/8581 (4·0%)	54·1% (44·7 to 61·9)
		Two standard doses	390	132/7201 (1·8%)	258/7179 (3·6%)	49·5% (37·7 to 59·0)
		Low dose plus standard dose	117	29/1396 (2·1%)	88/1402 (6·3%)	67·6% (50·8 to 78·7)
**Exploratory analyses by prime-boost interval**						
Primary symptomatic COVID-19 cases more than 14 days after second dose						
	Prime-boost interval (two standard doses)					
		<6 weeks	111	35/3890 (0·9%)	76/3856 (2·0%)	55·1% (33·0 to 69·9)
		6–8 weeks	64	20/1112 (1·8%)	44/1009 (4·4%)	59·9% (32·0 to 76·4)
		9–11 weeks	43	11/906 (1·2%)	32/958 (3·3%)	63·7% (28·0 to 81·7)
		≥12 weeks	53	8/1293 (0·6%)	45/1356 (3·3%)	81·3% (60·3 to 91·2)
	Prime-boost interval (two standard doses or low dose plus standard dose)					
		<6 weeks	111	35/3905 (0·9%)	76/3871 (2·0%)	55·1% (33·0 to 69·9)
		6–8 weeks	64	20/1124 (1·8%)	44/1023 (4·3%)	59·7% (31·7 to 76·3)
		9–11 weeks	66	14/1530 (0·9%)	52/1594 (3·3%)	72·2% (50·0 to 84·6)
		≥12 weeks	91	15/2038 (0·7%)	76/2093 (3·6%)	80·0% (65·2 to 88·5)
Asymptomatic COVID-19 cases more than 14 days after second dose (COV002 only)						
	Prime-boost interval (two standard doses)					
		<6 weeks	17	9/728 (1·2%)	8/733 (1·1%)	−11·8% (−189·5 to 56·8)
		6–8 weeks	21	14/528 (2·7%)	7/476 (1·5%)	−74·2% (−330·3 to 29·5)
		9–11 weeks	17	6/599 (1·0%)	11/666 (1·7%)	39·9% (−62·3 to 77·8)
		≥12 weeks	28	12/837 (1·4%)	16/876 (1·8%)	22·8% (−63·3 to 63·5)
	Prime-boost interval (two standard doses or low dose plus standard dose)					
		<6 weeks	17	9/728 (1·2%)	8/733 (1·1%)	−11·8% (−189·5 to 56·8)
		6–8 weeks	21	14/538 (2·6%)	7/488 (1·4%)	−75·7% (−334·2 to 28·9)
		9–11 weeks	43	17/1223 (1·4%)	26/1302 (2·0%)	31·6% (−26·0 to 62·8)
		≥12 weeks	49	17/1582 (1·1%)	32/1613 (2·0%)	47·2% (5·0 to 70·7)
Any NAAT-positive COVID-19 cases more than 14 days after second dose						
	Prime-boost interval (two standard doses)					
		<6 weeks	145	51/3890 (1·3%)	94/3856 (2·4%)	47·1% (25·6 to 62·4)
		6–8 weeks	90	39/1112 (3·5%)	51/1009 (5·1%)	32·6% (−2·2 to 55·5)
		9–11 weeks	68	18/906 (2·0%)	50/958 (5·2%)	61·9% (34·8 to 77·8)
		≥12 weeks	87	24/1293 (1·9%)	63/1356 (4·6%)	59·9% (35·8 to 75·0)
	Prime-boost interval (two standard doses or low dose plus standard dose)					
		<6 weeks	145	51/3905 (1·3%)	94/3871 (2·4%)	47·1% (25·6 to 62·4)
		6–8 weeks	90	39/1124 (3·5%)	51/1023 (5·0%)	32·2% (−2·7 to 55·3)
		9–11 weeks	122	33/1530 (2·2%)	89/1594 (5·6%)	61·8% (43·1 to 74·3)
		≥12 weeks	150	38/2038 (1·9%)	112/2093 (5·4%)	65·6% (50·3 to 76·2)

Data are number of cases/number of participants in the group (%), unless otherwise stated. Overall vaccine efficacy and 95% CIs were calculated using robust Poisson models, adjusting for study (COV001, COV002, COV003, or COV005) and age group (18–55 years, 56–69 years, or ≥70 years), with an offset for length of time at risk. Models for asymptomatic or unknown infections do not adjust for study. Vaccine efficacy and 95% CIs in prime-boost subgroups and for low dose plus standard dose subgroups have been calculated using unadjusted robust Poisson models. Any NAAT-positive includes primary symptomatic cases, non-primary symptomatic cases (not shown separately), asymptomatic or unknown infections in the UK, and asymptomatic infections in Brazil and South Africa (not shown separately).

* Calculated from an unadjusted robust Poisson model.

† p value for interaction term comparing low dose plus standard dose with two standard doses is p=0·050.

From the day of vaccination, two participants were admitted to hospital with COVID-19 in the ChAdOx1 nCoV-19 group^4^ (on day 0 and day 10) and 22 in the control group, three of whom were considered to have severe COVID-19. Vaccine efficacy against COVID-19 requiring hospital admission from 22 days after the first dose was 100% (0 cases in the ChAdOx1 nCoV-19 group *vs* 15 cases in the control group), with a lower bound of the one-sided 97·5% CI of 72·2% (appendix p 5).

There were 130 cases of asymptomatic infection occurring more than 14 days after the booster dose (COV002 UK cohort only), with efficacy of 22·2% (95% CI –9·9 to 45·0; table 1). In the participants who received two standard doses, there was no evidence of protection, with vaccine efficacy of 2·0% (–50·7 to 36·2; 41 cases in the ChAdOx1 nCoV-19 group *vs* 42 in the control group). In the cohort receiving a low dose plus standard dose, there were 47 cases and vaccine efficacy was 49·3% (7·4 to 72·2; 16 cases *vs* 31 cases). Efficacy against any NAAT-positive infection was 54·1% (44·7 to 61·9), indicating the potential for a reduction of transmission.

108 (0·9%) of 12 282 participants in the ChAdOx1 nCoV-19 group and 127 (1·1%) of 11 962 participants in the control group had serious adverse events (appendix p 14). The most common serious adverse events were infections and infestations in 23 (0·2%) participants in the ChAdOx1 nCoV-19 group and 41 (0·3%) in the control group. The adverse event profile was similar across vaccine groups. 211 (1·7%) of 12 282 participants who received ChAdOx1 nCoV-19 had a grade 3 adverse event versus 164 (1·4%) of 11 962 in the control group. Grade 4 adverse events were recorded in 48 (0·4%) participants who received ChAdOx1 nCoV-19 and 34 (0·3%) control participants (appendix p 19). There were seven deaths considered unrelated to vaccination (two in the ChAdOx1 nCov-19 group and five in the control group), including one COVID-19-related death in one participant in the control group.

**Figure 1:**
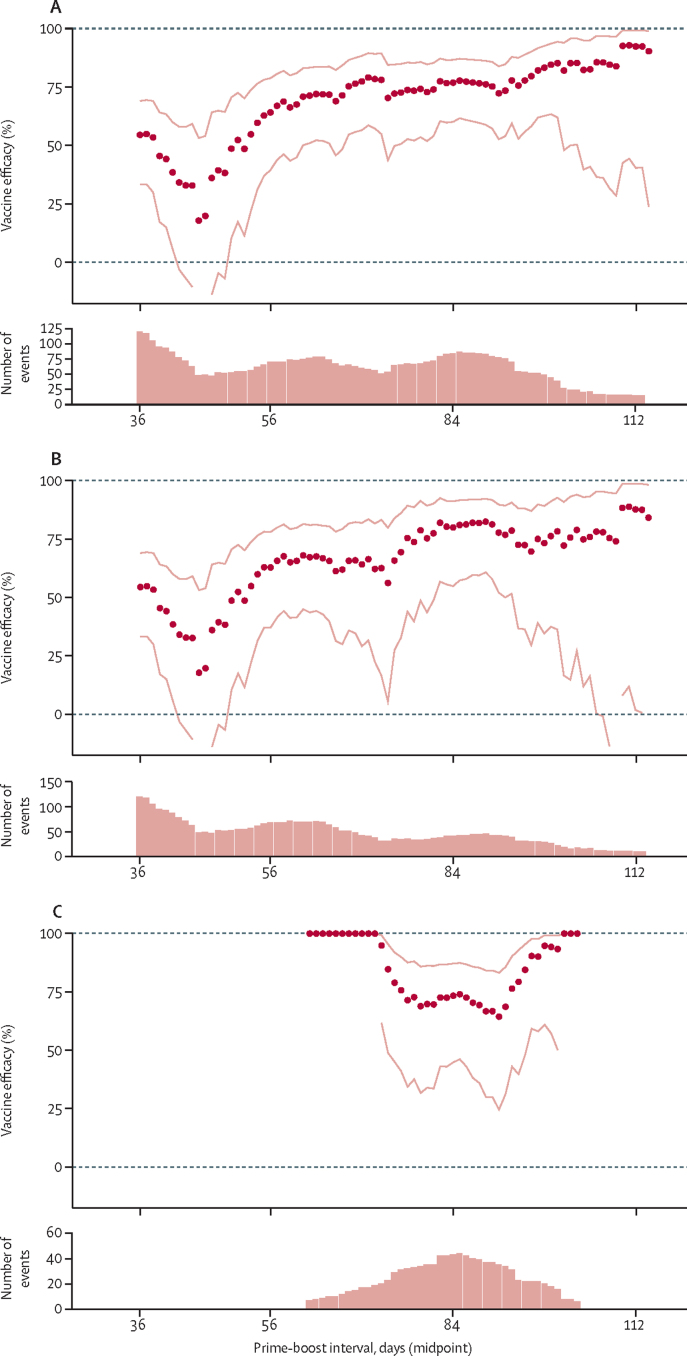
Exploratory analysis of vaccine efficacy against primary symptomatic COVID-19 more than 14 days after a booster dose, by prime-boost interval (A) All participants who received two doses. (B) Participants who received two standard doses. (C) Participants who received a low dose plus standard dose. Each datapoint shows one estimate of vaccine efficacy calculated in a subset of participants who received two doses of vaccine with a prime-boost interval falling within a 20-day interval. The x-axis shows the midpoint of the interval such that the first datapoint, plotted at 36 days, includes data from participants who received vaccines between 26 and 46 days apart. Estimates are from unadjusted log-binomial models. Dotted lines show 95% CIs for each point estimate of vaccine efficacy. Bar charts below each plot show the number of events included in each efficacy analysis.

An exploratory analysis modelling the change in vaccine efficacy against primary symptomatic COVID-19 (from 14 days after the second dose) showed that efficacy was high after a 2-month prime-boost interval and continued to increase with longer dose intervals (figure 1A, B). There was less variation in the prime-boost interval for the low dose plus standard dose cohort, with most data accruing in those who had about 3 months between first and second doses, and efficacy remained high during this period (figure 1C). Vaccine efficacy after two standard doses was 55·1% (95% CI 33·0–69·9) with an interval of less than 6 weeks and 81·3% (60·3–91·2) when more than 12 weeks apart (table 1).

Efficacy against asymptomatic infections in the UK showed a similar pattern, with efficacy estimates increasing as the interval between doses increased; however, the number of cases available for each analysis was small within each dose interval bracket and CIs were wide (table 1, appendix p 7).

**Figure 2:**
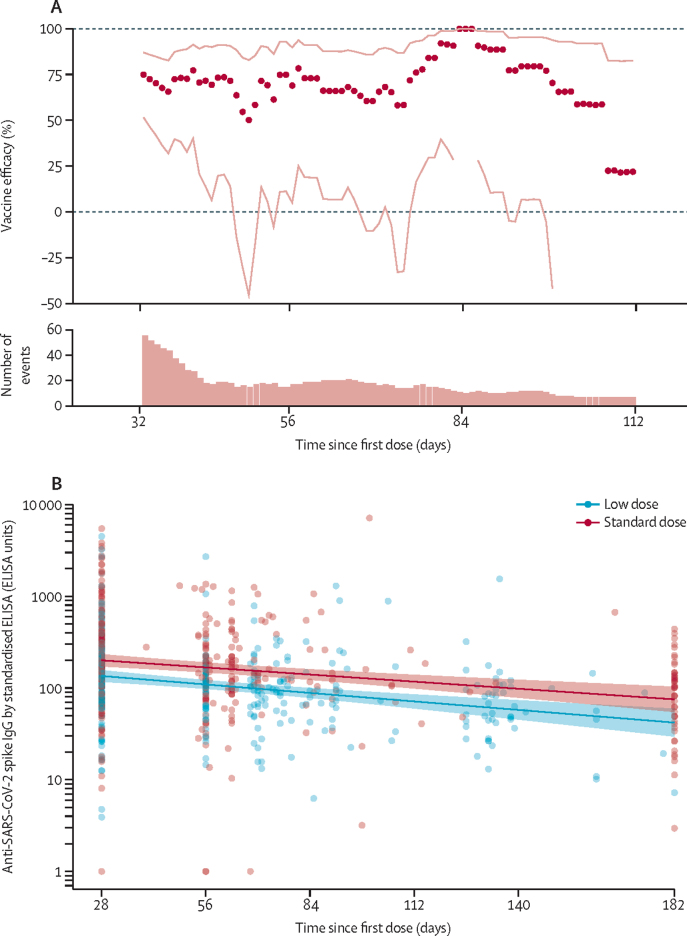
Exploratory analysis of vaccine efficacy over time from 22 days after a single standard dose ChAdOx1 nCoV-19 (A) and persistence of anti-SARS-CoV-2 spike IgG by standardised ELISA antibody after a single dose of either standard-dose or low-dose vaccine (B) (A) Each datapoint shows one estimate of vaccine efficacy calculated in a subset of participants who were followed up during a 21-day period after their first dose (n=18 548). Datapoints are plotted on the x-axis at the midpoint of the follow-up period. For each estimate of vaccine efficacy, cases were censored if they occurred before or after the 21-day period, such that the first datapoint, plotted at 33 days, shows vaccine efficacy in the 3 weeks from 22 days to 43 days after vaccination. Lines show 95% CIs for each point estimate of vaccine efficacy. The bar chart shows the number of participants with COVID-19 included in each model. Data are also in table 2. (B) Solid lines show estimates from a linear model with shaded areas showing SEs. Samples included are from participants given a single dose in study COV001 who had a blood sample taken at 6 months (n=44), and participants in COV002 who had a blood sample taken at the time of their booster dose (n=264). Timings for booster doses varied. All participants had blood taken at day 28. Because of laboratory capacity, samples from all participants in the trial have not all been processed on this assay.

Protection against primary symptomatic COVID-19 with a single standard dose vaccine was modelled against time since first dose. Results showed no evidence of waning of protection in the first 3 months after vaccination (figure 2A). A single standard dose of vaccine provided protection against primary symptomatic COVID-19 in the first 90 days with an efficacy of 76·0% (95% CI 59·3 to 85·9), but was not efficacious against asymptomatic infection over the same time period (vaccine efficacy –17·2% [–248·6 to 60·6]; table 2). Efficacy of a single standard dose against any NAAT-positive infection was 63·9% (46·0 to 75·9) from 22 days to 90 days, suggesting the potential for a substantial reduction in transmission, although these results are exploratory and require further investigation.

**Table 2:** Efficacy of ChAdOx1 nCoV-19 more than 21 days after a single dose

		**Total cases**	**ChAdOx1 nCoV-19**	**Control**	**Vaccine efficacy (95% CI)**
**Primary symptomatic COVID-19 cases more than 21 days after a single standard dose**					
Time since first standard dose					
	22 to 30 days	37	7/9257 (0·1%)	30/9237 (0·3%)	76·7% (47·0 to 89·8)
	31 to 60 days	28	6/7147 (0·1%)	22/7110 (0·3%)	72·8% (32·9 to 89·0)
	61 to 90 days	23	4/2885 (0·1%)	19/2974 (0·6%)	78·3% (36·4 to 92·6)
	91 to 120 days	10	4/1369 (0·3%)	6/1404 (0·4%)	31·6% (−141·8 to 80·7)
	22 to 90 days	88	17/9257 (0·2%)	71/9237 (0·8%)	76·0% (59·3 to 85·9)
**Asymptomatic COVID-19 infections more than 21 days after a single standard dose (UK COV002 only)**					
Time since first dose					
	22 to 30 days	6	3/3236 (0·1%)	3/3239 (0·1%)	−0·1% (−395·4 to 79·8)
	31 to 60 days	6	4/2703 (0·1%)	2/2687 (0·1%)	−100·1% (−992·2 to 63·3)
	61 to 90 days	1	0/1843 (0·0%)	1/1891 (0·1%)	..
	91 to 120 days	4	1/780 (0·1%)	3/765 (0·4%)	67·6% (−210·8 to 96·6)
	22 to 90 days	13	7/3236 (0·2%)	6/3239 (0·2%)	−17·2% (−248·6 to 60·6)
**Any NAAT-positive COVID-19 infections more than 21 days after a single standard dose**					
Time since first dose					
	22 to 30 days	51	14/9257 (0·2%)	37/9237 (0·4%)	62·3% (30·2 to 79·6)
	31 to 60 days	46	14/7147 (0·2%)	32/7110 (0·5%)	56·3% (18·2 to 76·7)
	61 to 90 days	24	4/2885 (0·1%)	20/2974 (0·7%)	79·4% (39·8 to 93·0)
	91 to 120 days	17	7/1369 (0·5%)	10/1404 (0·7%)	28·2% (−88·1 to 72·6)
	22 to 90 days	121	32/9257 (0·3%)	89/9237 (1·0%)	63·9% (46·0 to 75·9)

Data are number of cases/number of participants in the group (%), unless otherwise specified. Vaccine efficacy and 95% CIs were calculated via unadjusted robust Poisson models. Participants were censored in the analysis at the upper limit of the time window. Any NAAT-positive includes primary symptomatic cases, non-primary symptomatic cases (not shown separately), asymptomatic or unknown infections in the UK, and asymptomatic infections in Brazil and South Africa (not shown separately).

Participants included in the analysis of a single dose were further assessed to identify differences in baseline characteristics between those who received a booster dose (and are censored in the analysis at that timepoint) and those who did not receive a booster dose (and thus have longer follow-up). Statistically significant differences between these groups were found for age, sex, health or social care worker status, dose (low dose plus standard dose *vs* two standard doses), country, ethnicity, and follow-up time (all p<0·0001 except ethnicity which was p=0·0001; appendix p 6). Participants receiving a booster dose were older (median age 40 years [IQR 30–52] in those who received a booster dose *vs* 36 years [28–48] for those who did not), with a higher proportion of men (8471 [44·2%] of 19 150 *vs* 1072 [39·0%] of 2752) and non-white participants (4615 [24·1%] of 19 150 *vs* 571 [20·8%] of 2751), and a smaller proportion of health or social care workers (11 518 [60·1%] of 19 150 *vs* 1809 [65·7%] of 2752) compared with those who did not receive a booster dose. A smaller proportion of UK COV002 participants who received a booster dose received a low-dose prime vaccination (2894 [33·4%] of 8676 participants) than in those who did not receive a booster (480 [40·9%] of 1173). Follow-up time differed between the two groups, as expected because of the censoring of participants at the time of booster dose (median time 20 days [IQR 10·0–58·0] in those receiving a booster dose *vs* 90 days [23·0–157·0] in those who did not). Adjustment for baseline factors did affect vaccine efficacy estimates (appendix p 6).

Anti-SARS-CoV-2 spike IgG responses to a single dose of vaccine measured by standardised ELISA decayed log-linearly over a 6-month period. Geometric mean antibody decay estimated in a linear model showed a decrease from the peak at day 28, of 34% by day 90 (geometric mean ratio [GMR] 0·66 [95% CI 0·59–0·74]) and by 64% by day 180 (GMR 0·36 [0·27–0·47]; figure 2B).

**Figure 3:**
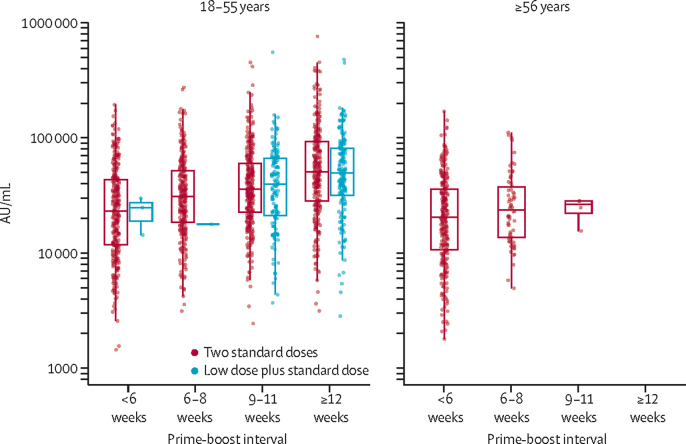
Anti-SARS-CoV-2 spike IgG responses by multiplex immunoassay at 28 days after the second dose in participants receiving two standard doses or low dose plus standard dose, by prime-boost interval (n=3337) Participants who were NAAT positive before the blood sample taken at day 28 were not included in the analyses. About 15% of samples from participants were analysed using this assay. The midlines of the boxes show medians and the outer bounds of the boxes show IQRs. Error bars show 1·5 × the IQR above or below the 75th or 25th percentile. Data are also in the appendix (p 9).

Participants aged 18–55 years who received a second standard vaccine more than 12 weeks after the first had antibody titres more than two-fold higher than those who received the second dose within 6 weeks of their initial vaccination (GMR 2·32 [95% CI 2·01–2·68]; figure 3; appendix pp 9–10). Similarly, neutralising antibody titres measured by pseudovirus were higher after a longer interval before the second dose (appendix p 11).

**Figure 4:**
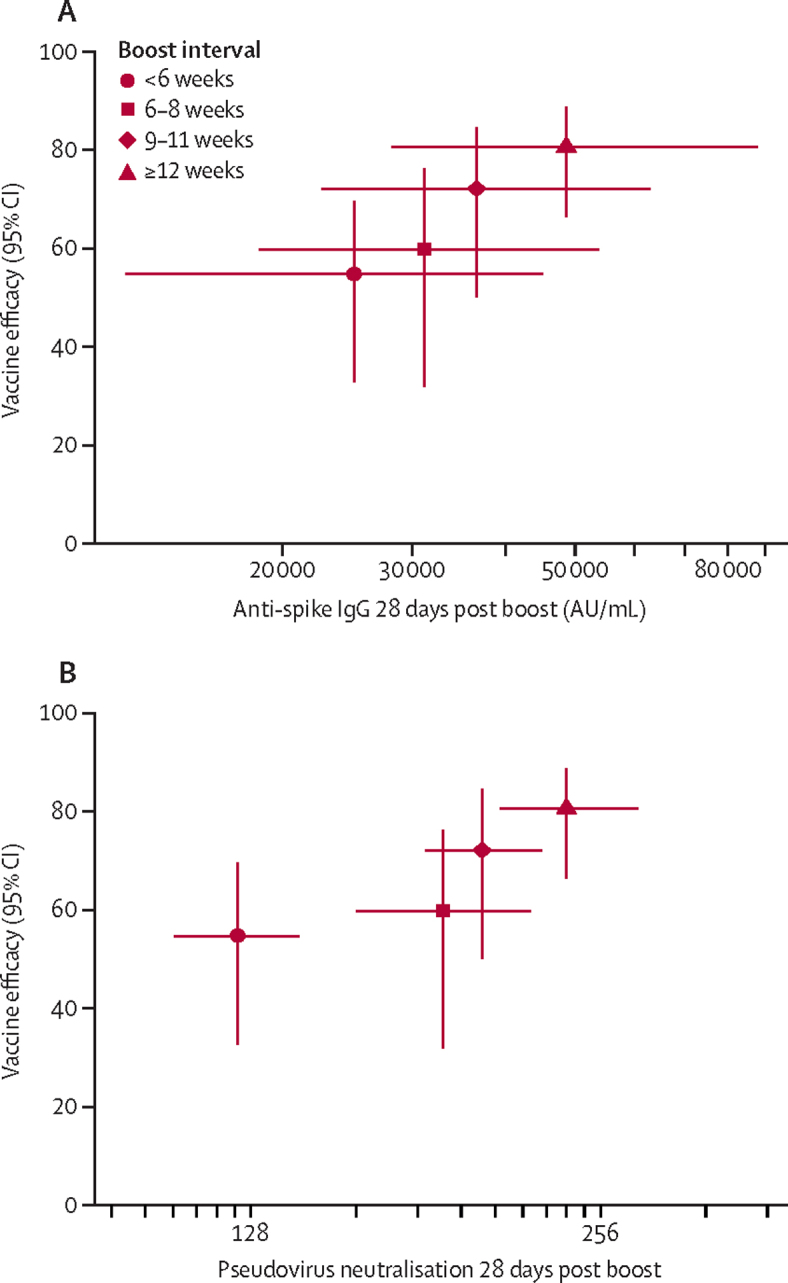
Relationship between binding and neutralising antibody 28 days after second dose, and vaccine efficacy against primary symptomatic COVID-19 Vaccine efficacy with 95% CI against primary symptomatic COVID-19 in participants who received two standard doses and those who received a low dose plus standard dose combined are shown plotted against the GMT (95% CI) of anti-SARS-CoV-2 spike IgG from an immunoassay (A), and the GMT (95% CI) pseudovirus neutralisation (B), for each prime boost interval. GMT=geometric meant titre.

Plotting anti-SARS-CoV-2 spike IgG against vaccine efficacy for each dose interval showed evidence of a potential relationship between binding antibody and vaccine protection, as well as between neutralisation antibody and vaccine efficacy, suggesting potential correlates of protection (figure 4).

Kaplan-Meier cumulative incidence of primary symptomatic COVID-19 showed clear differentiation between ChAdOx1 nCoV-19 and control groups, with non-overlapping confidence intervals, in line with efficacy estimates (appendix pp 12–13).

## Discussion

Here, we report a prespecified full primary analysis of the efficacy of the ChAdOx1 nCoV-19 vaccine more than 14 days after a second dose, with 332 symptomatic cases of COVID-19 in an analysis population of 17 178 study participants and an efficacy of 66·7% (95% CI 57·4–74·0), confirming the results of our published interim analysis^4^ (131 cases reported in the interim analysis). In this updated analysis, there were no additional hospital admissions or cases of severe COVID-19 in the ChAdOx1 nCoV-19-vaccinated group after the initial 21-day period after vaccination, compared with 15 in the control group. These new analyses provide important verification of the interim data that underpinned the emergency use authorisation of the vaccine in the UK by the Medicines and Healthcare products Regulatory Agency (MHRA) on Dec 30, 2020,^14^ and other international regulators since the end of 2020, including the European Medicines Agency and regulators in India, Nepal, Bangladesh, Argentina, Brazil, and Mexico.

The analysis presented here provides strong evidence for the efficacy of two standard doses of the vaccine, which is the regimen approved by the MHRA and other regulators. Following regulatory approval, a key question for policy makers to plan the optimal approach for roll-out is the optimal dose interval, which is assessed in this report through post-hoc exploratory analyses. Two criteria that contribute to decision making in this area are the effect of prime-boost interval on protection after the second dose and the degree to which the vaccinated individual is at risk of infection during the time period before the booster dose, if there were either reduced efficacy with a single dose or rapid waning of efficacy before the second vaccination.

Exploratory analyses are presented in this report that show protection with dosing intervals from less than 6 weeks to 12 weeks or more and that a longer interval provides better protection after a booster dose without compromising protection in a 3-month period before the second dose is administered.

In exploratory analyses, a single standard dose of ChAdOx1 nCoV-19 had an efficacy of 76·0% (95% CI 59·3 to 85·9) against symptomatic COVD-19 in the first 90 days after vaccination, with no significant waning of protection during this period. It is not clear how long protection might last with a single dose because follow-up is limited to the time periods described here, and, for this reason, a second dose of vaccine is recommended.

A second dose of ChAdOx1 nCoV-19 induces increased neutralising antibody levels^10^, ^12^ and is probably necessary for long-lasting protection. However, where there is low supply of vaccine, a policy of initially vaccinating a larger cohort with a single dose might provide better overall population protection than vaccinating half the number of individuals with two doses in the short term. With the evidence available at this time, it is anticipated that a second dose is still required to potentiate long-lived immunity. Recent modelling of delayed boosting suggests that even in the presence of substantial waning of first-dose efficacy, programmes that delay a second dose to vaccinate a larger proportion of the population result in greater immediate overall population protection.^15^

In our study, vaccine efficacy after the second dose was higher in those with a longer prime-boost interval, reaching 81·3% in those with a dosing interval of 12 weeks or more versus 55·1% in those with an interval of less than 6 weeks. Point estimates of efficacy were lower with shorter dosing intervals, although it should be noted that there is some uncertainty because the CIs overlap. Higher binding and neutralising antibody titres were observed in sera with the longer prime-boost interval, suggesting that, assuming there is a relationship between the humoral immune response and efficacy, these might be true findings and not artifacts of the data. Greater protective efficacy associated with stronger immune responses after a wider prime-boost interval have been seen with other vaccines such as those for influenza, Ebola virus disease, and malaria.^16^, ^17^, ^18^ The findings presented here for the ChAdOx1 nCoV-19 vaccine are consistent with policy recommendations in different countries to use dose intervals of 4–12 weeks for this vaccine.

The slightly lower vaccine efficacy against symptomatic COVID-19 of 66·7% after a booster dose appears counterintuitive compared with the 76% efficacy after a single dose, although these differences are non-significant. Cases included in single-dose estimates occurred earlier in the year than those included in post-second-dose analyses, and the intensity of the epidemics varied in the different countries, making single-dose and two-dose estimates difficult to directly compare with each other.

In our interim analysis, we identified a higher efficacy in a subgroup analysis of those who received the low dose plus standard dose regimen.^4^ This finding is supported by the results of this analysis, although these findings are exploratory. With further data available, we show that the enhanced immunogenicity and efficacy with this regimen might be partly driven by the longer dosing interval that was a feature of this subgroup, further supporting the observation of a relationship between dose interval and efficacy in those who received two standard doses. The two standard doses regimen is preferred operationally because it is more straightforward to deliver the same vaccine for both doses and because there are more immunogenicity and efficacy data to support its use.

A further important question is whether vaccines can reduce transmission, and therefore combined with physical distancing measures contribute to reductions in human-to-human transmission of the virus. Although transmission studies were not included in the analysis, swabs were obtained from volunteers every week in the UK study, regardless of symptoms, to allow assessment of the overall effect of the vaccine on risk of infection, and thus a surrogate for potential onward transmission. If there was no effect of a vaccine on asymptomatic infection (about a third of infections), it would be expected that an efficacious vaccine would simply convert severe cases to mild cases and mild cases to asymptomatic, with overall NAAT positivity unchanged. Overall NAAT positivity is appropriate to assess whether there is a reduction in the burden of infection. We showed that a single standard dose of the vaccine had efficacy against any NAAT-positive infection, including symptomatic and asymptomatic infections, of 63·9% between day 22 and 90 after first dose, and that, after the second dose, the two standard doses schedule had an efficacy of 49·5%. These data indicate that ChAdOx1 nCoV-19, used in the authorised schedules, might have a substantial effect on transmission by reducing the number of infected individuals in the population. Notably, asymptomatic infections were only measured in the UK. Vaccine efficacy against any NAAT-positive infection after a second dose appears lower than single-dose efficacy, probably because of the larger proportion of UK cases in the analysis and therefore the larger number of asymptomatic infections included, for which efficacy is lower.

No correlate of protection has yet been defined for COVID-19 vaccines; however, the data presented here on the relationship between antibody levels and efficacy suggest that humoral immunity might play a role. By contrast, high protective efficacy recorded early after a single dose of vaccine in this study, and also seen with other vaccines from different manufacturers,^3^ suggests other immunological mechanisms might be at play early after the first dose, because lower levels of neutralising antibody are detected after a single dose. Further study of correlates of protection is ongoing.

There are some limitations to the analyses presented in this report. The studies were not designed to establish whether vaccine efficacy differed by dose interval and the presence of data of varying intervals arose because of the logistics of running large-scale clinical trials in a pandemic setting. These are therefore post-hoc exploratory analyses only with potential for multiple sources of bias, and were not prespecified. However, the analyses are presented here to provide a rigorous peer-reviewed interrogation of updated data that reflect the approach that is currently being used to underpin the deployment of ChAdOx1 nCoV-19 in the response to the pandemic. The previous interim analysis was carefully considered by regulators and policy makers and is aligned with the findings presented here.

In our data, the length of follow-up after the second dose was short and follow-up tends to be longer in those who were boosted early and thus have shorter prime-boost intervals. Furthermore, the participants who contribute to the analysis of single-dose efficacy are a mixture of participants with events occurring before their booster dose and participants who did not receive a booster dose. These two cohorts differ in some key characteristics; participants who received a booster dose were slightly younger, a greater proportion were men, and a smaller proportion were white compared with those who did not receive a booster dose.

It is not clear what effect each of these individual sources of variation in the data have on vaccine efficacy estimates. However, the same trend seen with efficacy is also seen in the immunological data, suggesting an underlying biological mechanism.

Vaccination programmes aimed at vaccinating a large proportion of the population with a single dose, with a second dose given after a 3-month period, might be an effective strategy for reducing disease, and might have advantages over a programme with a short prime-boost interval for roll-out of a pandemic vaccine when supplies are scarce in the short term. Two doses of ChAdOx1 nCoV-19 was efficacious in preventing symptomatic COVID-19. These results confirm those seen in the interim analysis of the trials.

**This online publication has been corrected. The corrected version first appeared at thelancet.com on March 4, 2021**

## Data sharing

Anonymised participant data will be made available when the trials are complete, upon requests directed to the corresponding author. Proposals will be reviewed and approved by the sponsor, investigator, and collaborators on the basis of scientific merit. After approval of a proposal, data can be shared through a secure online platform after signing a data access agreement. All data will be made available for a minimum of 5 years from the end of the trial.

## References

[1] WHO (Feb 16, 2021). The COVID-19 candidate vaccine landscape. https://www.who.int/publications/m/item/draft-landscape-of-covid-19-candidate-vaccines.

[2] Baden LR, El Sahly HM, Essink B (2021). Efficacy and safety of the mRNA-1273 SARS-CoV-2 vaccine. N Engl J Med.

[3] Polack FP, Thomas SJ, Kitchin N (2020). Safety and efficacy of the BNT162b2 mRNA Covid-19 vaccine. N Engl J Med.

[4] Voysey M, Costa Clemens SA, Madhi SA (2021). Safety and efficacy of the ChAdOx1 nCoV-19 vaccine (AZD1222) against SARS-CoV-2: an interim analysis of four randomised controlled trials in Brazil, South Africa, and the UK. Lancet.

[5] UK Department of Health and Social Care (Dec 30, 2020). Statement from the UK Chief Medical Officers on the prioritisation of first doses of COVID-19 vaccines. https://www.gov.uk/government/news/statement-from-the-uk-chief-medical-officers-on-the-prioritisation-of-first-doses-of-covid-19-vaccines.

[6] UK Department of Health and Social Care (Dec 30, 2020). Oxford University/AstraZeneca vaccine authorised by UK medicines regulator. https://www.gov.uk/government/news/oxford-universityastrazeneca-vaccine-authorised-by-uk-medicines-regulator.

[7] Rinke A, Skydsgaard N (Jan 4, 2021). Germany mulls delaying second COVID-19 vaccine shot, Denmark approves delay. https://www.reuters.com/article/uk-health-coronavirus-vaccines-germany/germany-mulls-delaying-second-covid-19-vaccine-shot-denmark-approves-delay-idUKKBN2991Q3?edition-redirect=uk.

[8] Wu KJ, Robbins R (Jan 8, 2021). As rollout falters, scientists debate new vaccination tactics. https://www.nytimes.com/2021/01/03/health/coronavirus-vaccine-doses.html.

[9] Strategic Advisory Group of Experts (SAGE) on Immunization Working Group on COVID-19 vaccines (Dec 22, 2020). mRNA vaccines against COVID-19: Pfizer-BioNTech COVID-19 vaccine BNT162b2. https://apps.who.int/iris/bitstream/handle/10665/338096/WHO-2019-nCoV-vaccines-SAGE_evaluation-BNT162b2-2020.1-eng.pdf?sequence=1&isAllowed=y.

[10] Barrett JR, Belij-Rammerstorfer S, Dold C (2020). Phase 1/2 trial of SARS-CoV-2 vaccine ChAdOx1 nCoV-19 with a booster dose induces multifunctional antibody responses. Nat Med.

[11] Ewer KJ, Barrett JR, Belij-Rammerstorfer S (2020). T cell and antibody responses induced by a single dose of ChAdOx1 nCoV-19 (AZD1222) vaccine in a phase 1/2 clinical trial. Nat Med.

[12] Folegatti PM, Ewer KJ, Aley PK (2020). Safety and immunogenicity of the ChAdOx1 nCoV-19 vaccine against SARS-CoV-2: a preliminary report of a phase 1/2, single-blind, randomised controlled trial. Lancet.

[13] Ramasamy MN, Minassian AM, Ewer KJ (2021). Safety and immunogenicity of ChAdOx1 nCoV-19 vaccine administered in a prime-boost regimen in young and old adults (COV002): a single-blind, randomised, controlled, phase 2/3 trial. Lancet.

[14] Medicines and Healthcare products Regulatory Agency (Jan 28, 2021). Regulatory approval of COVID-19 Vaccine AstraZeneca. https://www.gov.uk/government/publications/regulatory-approval-of-covid-19-vaccine-astrazeneca.

[15] Jurgens G (2021). Modelling decay of population immunity with proposed second dose deferral strategy. medRxiv.

[16] Ewer K, Rampling T, Venkatraman N (2016). A monovalent chimpanzee adenovirus Ebola vaccine boosted with MVA. N Engl J Med.

[17] Fernandez-Arias C, Arias CF, Zhang M, Herrero MA, Acosta FJ, Tsuji M (2018). Modeling the effect of boost timing in murine irradiated sporozoite prime-boost vaccines. PLoS One.

[18] Ledgerwood JE, Zephir K, Hu Z (2013). Prime-boost interval matters: a randomized phase 1 study to identify the minimum interval necessary to observe the H5 DNA influenza vaccine priming effect. J Infect Dis.

