# Supplementary material for: Single-dose administration and the influence of the timing of the booster dose on immunogenicity and efficacy of ChAdOx1 nCoV-19 (AZD1222) vaccine: a pooled analysis of four randomised trials
Source: Lancet. 2021 Mar 6;397(10277):881–91. doi: 10.1016/S0140-6736(21)00432-3 (PMC7894131; doi:10.1016/S0140-6736(21)00432-3)
Supplement: Supplementary appendix [file mmc1.pdf]

THE LANCET

Supplementary appendix

This appendix formed part of the original submission and has been peer reviewed. We post it as supplied by the authors.

Supplement to: Voysey M, Clemens SAC, Madhi SA, et al. Single-dose administration and the influence of the timing of the booster dose on immunogenicity and efficacy of ChAdOx1 nCoV-19 (AZD1222) vaccine: a pooled analysis of four randomised trials. *Lancet* 2021; published online Feb 19. http://dx.doi.org/10.1016/S0140-6736(21)00432-3.

Supplementary File

Table of Contents

Figure S1 CONSORT diagram 2

Table S1 Baseline characteristics of primary efficacy analysis cohort 3

Table S2 Follow-up time (days) after 14 days post-booster dose in primary efficacy cohort, by country 4

Table S3 Hospitalisation for COVID-19 (Any dose population) 5

Table S4 Factors related to receipt of a booster dose 6

Figure S2 Vaccine efficacy against asymptomatic/unknown infection by interval between first and second dose after, A) SD/SD or LD/SD, B) SD/SD, C) LD/SD administration 7

Table S5 SARS-CoV-2 anti-spike IgG responses by multiplex immunoassay at 28 days after a second dose, by prime-boost interval 9

Figure S3 Neutralising antibody 28 days after a booster dose, measured in pseudovirus assay (Monogram IC50) 11

Table S6 Neutralising antibody 28 days after a booster dose, measured in pseudovirus assay (Monogram IC50) 11

Figure S4 Kaplan-Meier cumulative incidence of primary symptomatic PCR+ cases after booster dose, in the primary efficacy cohort 12

Figure S5 Kaplan-Meier cumulative incidence of primary symptomatic PCR+ cases after booster dose in SD/SD cohorts, by country* 13

Table S7 Serious Adverse Events by System Organ Class and Preferred Term 14

Table S8 Unsolicited Adverse Events by System Organ Class and Preferred Term (severity grade 3 or higher) 19

The Oxford Trial Group List 27

Acknowledgements 52

Figure S1 CONSORT diagram

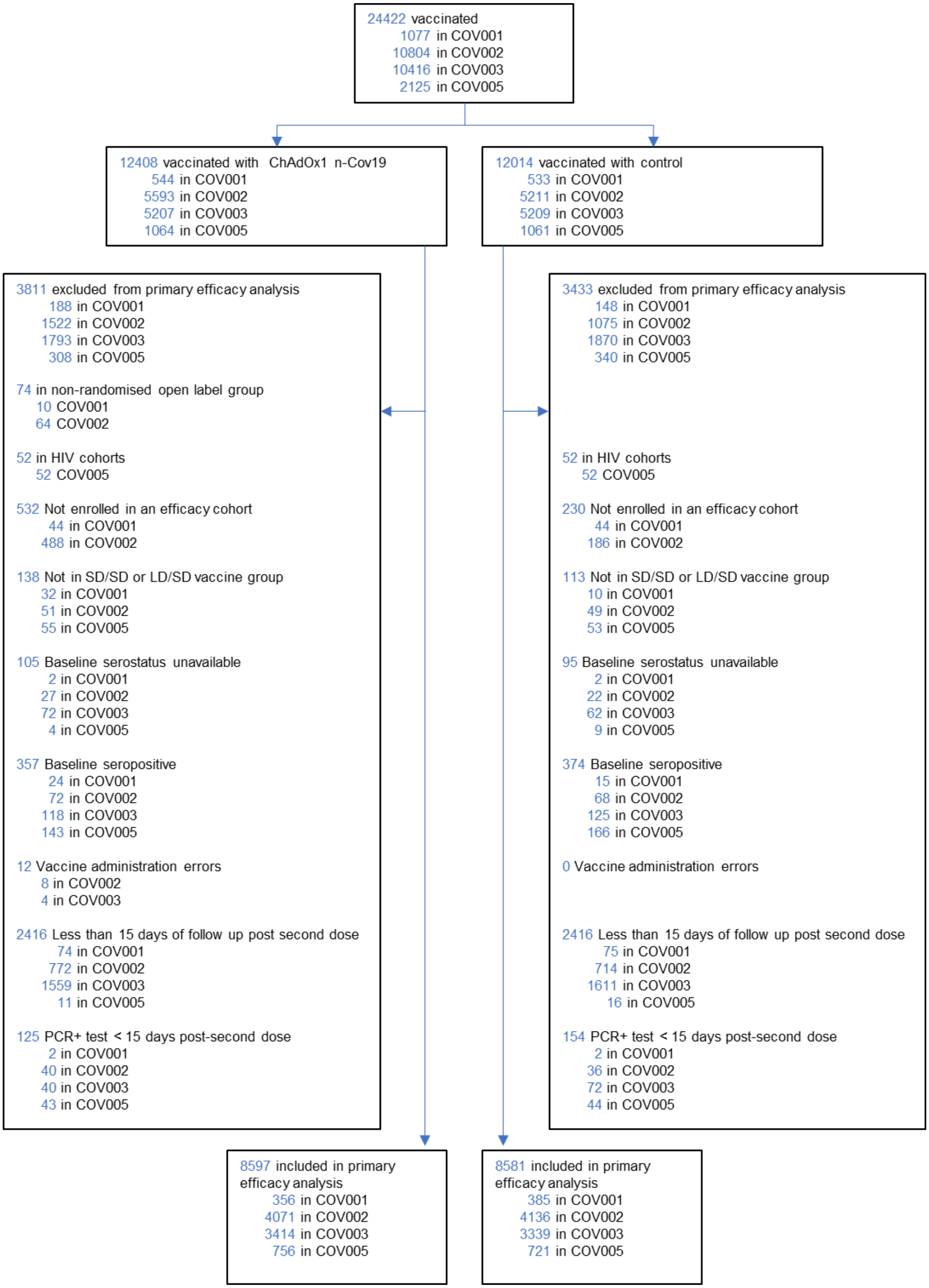

Table S1 Baseline characteristics of primary efficacy analysis two-dose cohort

Characteristics	COV001 (UK)		COV002 (UK)		COV003 (Brazil)		COV005 (South Africa)	
	**ChAdOx1 nCoV-19 (n=356)**	**Control (n=385)**	**ChAdOx1 nCoV-19 (n=4071)**	**Control (n=4136)**	**ChAdOx1 nCoV-19 (n=3414)**	**Control (n=3339)**	**ChAdOx1 nCoV-19 (n=756)**	**Control (n=721)**
Age								
18-55 years	356 (100.0%)	385 (100.0%)	3282 (80.6%)	3334 (80.6%)	2850 (83.5%)	2808 (84.1%)	719 (95.1%)	679 (94.2%)
56-69 years	0 (0.0%)	0 (0.0%)	377 (9.3%)	378 (9.1%)	492 (14.4%)	466 (14.0%)	37 (4.9%)	42 (5.8%)
≥70 years	0 (0.0%)	0 (0.0%)	412 (10.1%)	424 (10.3%)	72 (2.1%)	65 (1.9%)	0 (0.0%)£	0 (0.0%)£
missing	0 (0.0%)	0 (0.0%)	0 (0.0%)	0 (0.0%)	0 (0.0%)	0 (0.0%)	0 (0.0%)	0 (0.0%)
Sex (female) n%	174 (48.9%)	201 (52.2%)	2395 (58.8%)	2521 (61.0%)	1936 (56.7%)	1839 (55.1%)	311 (41.1%)	319 (44.2%)
BMI (kg/m^2) median [IQR]	24.1 [22.3-26.7]	24.4 [21.9-26.9]	25.4 [22.9-28.7]	25.4 [22.9-29.0]	25.8 [23.2-29.2]	25.9 [23.2-29.4]	23.4 [20.4-27.7]	23.5 [20.8-28.3]
Ethnicity								
White	323 (90.7%)	355 (92.2%)	3725 (91.5%)	3815 (92.2%)	2273 (66.6%)	2249 (67.4%)	114 (15.1%)	121 (16.8%)
Black	3 (0.8%)	1 (0.3%)	23 (0.6%)	17 (0.4%)	337 (9.9%)	336 (10.1%)	510 (67.5%)	466 (64.6%)
Asian†	17 (4.8%)	16 (4.2%)	220 (5.4%)	203 (4.9%)	83 (2.4%)	66 (2.0%)		
Mixed	6 (1.7%)	7 (1.8%)	70 (1.7%)	66 (1.6%)	704 (20.6%)	670 (20.1%)	113 (14.9%)€	117 (16.2%)€
Other	7 (2.0%)	6 (1.6%)	33 (0.8%)	35 (0.8%)	17 (0.5%)	18 (0.5%)	19 (2.5%)	17 (2.4%)
missing	0 (0.0%)	0 (0.0%)	0 (0.0%)	0 (0.0%)	0 (0.0%)	0 (0.0%)	0 (0.0%)	0 (0.0%)
Health and social care setting workers n%	67 (18.8%)	63 (16.4%)	2731 (67.1%)	2825 (68.3%)	2526 (74.0%)	2425 (72.6%)	64 (8.5%)	79 (11.0%)
Co-morbidities								
Cardiovascular disease	0 (0.0%)	0 (0.0%)	476 (11.7%)	468 (11.3%)	540 (15.8%)	511 (15.3%)	24 (3.2%)	20 (2.8%)
Respiratory disease	0 (0.0%)	0 (0.0%)	489 (12.0%)	525 (12.7%)	354 (10.4%)	321 (9.6%)	29 (3.8%)	20 (2.8%)
Diabetes	0 (0.0%)	0 (0.0%)	93 (2.3%)	86 (2.1%)	141 (4.1%)	113 (3.4%)	3 (0.4%)	4 (0.6%)

† Asian not recorded as a category in South Africa. £ Maximum age at enrolment for eligibility was 65 years in South Africa. € This category recorded as ‘Coloured’ in original data.

Table S2 Follow-up time (days) after 14 days post-booster dose in primary efficacy cohort, by country

**Pooled data**		**ChAdOx1 nCoV-19**	**Control**
Time between prime-boost SD/SD	<6 weeks	54.0 [18.0, 81.0]	53.0 [17.0, 80.0]
	6-8 weeks	83.0 [53.0, 97.0]	82.0 [52.0, 95.0]
	9-11 weeks	83.0 [67.0, 91.8]	84.0 [66.0, 92.0]
	≥12 weeks	61.0 [54.0, 69.0]	62.0 [53.8, 69.0]
Time between prime-boost SD/SD or LD/SD	<6 weeks	54.0 [18.0, 81.0]	53.0 [17.0, 80.0]
	6-8 weeks	83.0 [53.0, 97.0]	83.0 [52.0, 95.0]
	9-11 weeks	90.0 [81.0, 96.0]	90.0 [80.0, 96.0]
	≥12 weeks	67.0 [59.0, 82.0]	67.0 [56.0, 82.0]
**UK**		**ChAdOx1 nCoV-19**	**Control**
Time between prime-boost SD/SD	<6 weeks	35.0 [21.0, 63.0]	35.0 [21.0, 63.0]
	6-8 weeks	97.0 [89.0, 102.0]	95.0 [59.0, 101.0]
	9-11 weeks	89.0 [83.0, 95.0]	89.0 [82.0, 95.0]
	≥12 weeks	63.0 [56.0, 69.2]	63.0 [55.0, 70.0]
Time between prime-boost SD/SD or LD/SD	<6 weeks	35.0 [21.0, 63.0]	35.0 [21.0, 63.0]
	6-8 weeks	97.0 [90.0, 102.0]	95.0 [67.0, 101.0]
	9-11 weeks	94.0 [88.0, 98.0]	92.0 [88.0, 97.0]
	≥12 weeks	68.0 [59.0, 82.0]	68.0 [59.0, 82.0]
**Brazil**		**ChAdOx1 nCoV-19**	**Control**
Time between prime-boost SD/SD	<6 weeks	35.0 [13.0, 80.0]	35.0 [13.0, 70.0]
	6-8 weeks	68.0 [45.0, 83.0]	69.0 [47.0, 84.0]
	9-11 weeks	63.0 [53.0, 69.0]	61.0 [53.0, 69.0]
	≥12 weeks	46.0 [39.0, 53.0]	45.0 [33.5, 52.0]
**South Africa**		**ChAdOx1 nCoV-19**	**Control**
Time between prime-boost SD/SD	<6 weeks	81.0 [63.0, 91.0]	80.0 [63.0, 90.0]
	6-8 weeks	62.0 [52.0, 62.0]	61.0 [53.0, 63.0]
	9-11 weeks	30.5 [23.8, 33.5]	55.0 [46.0, 55.0]
	≥12 weeks	-	-
Time between prime-boost SD/SD or LD/SD	<6 weeks	81.0 [63.0, 91.0]	80.0 [63.0, 90.0]
	6-8 weeks	62.0 [52.0, 62.0]	61.0 [52.0, 63.0]
	9-11 weeks	30.5 [23.8, 33.5]	55.0 [46.0, 55.0]
	≥12 weeks	-	-

Follow-up time presented as median [IQR] in days from 15 days after booster dose until the first occurrence of either withdrawal, PCR+ event, or date of data lock.

Table S3 Hospitalisation for COVID-19 (Any dose efficacy population)

	N cases	ChAdOx1 nCoV-19 N=11794	Control N=11776
< 22 days after a single dose	9	2	7
>= 22 days after the first dose and < 15 days post booster dose	6	0	6
>= 15 days post booster dose	9	0	9

*>=22 days after first dose, 0 vs 15 cases, VE: 100%, (97.5% one-sided CI 72.2%, NE). Estimate determined from stratified Poisson regression with Exact Conditional Method. NE: Not evaluated.

Table S4 Factors related to receipt of a booster dose

	Participants who received booster N=19150	Participants who were not boosted N=2752	P value*
Age, median [IQR]	40.0 [30.1, 52.0]	36.3 [28.0, 48.0]	<0.001
18 – 55 years, n (%)	15841/19150 (82.7%)	2377/2752 (86.4%)	<0.001
56 – 69 years, n (%)	2218/19150 (11.6%)	247/2752 (9.0%)	
≥70 years, n (%)	1091/19150 (5.7%)	128/2752 (4.7%)	
Sex			
Female, n (%)	10679/19150 (55.8%)	1680/2752 (61.0%)	<0.001
Male, n (%)	8471/19150 (44.2%)	1072/2752 (39.0%)	
Health or social care worker, n (%)	11518/19150 (60.1%)	1809/2752 (65.7%)	<0.001
Dose group (COV002 only)			
SD, n (%)	5782/8676 (66.6%)	693/1173 (59.1%)	<0.001
LD, n (%)	2894/8676 (33.4%)	480/1173 (40.9%)	
Country (single SD cohort only)			
UK (SD), n (%)	6566/16222 (40.5%)	838/2272 (36.9%)	<0.001
Brazil (SD), n (%)	8194/16222 (50.5%)	1389/2272 (61.1%)	
South Africa (SD), n (%)	1462/16222 (9.0%)	45/2272 (2.0%)	
Ethnicity			
White, n (%)	14532/19150 (75.9%)	2180/2751 (79.2%)	<0.001
Non-white, n (%)	4615/19150 (24.1%)	571/2751 (20.8%)	
Follow-up time from 21 days after prime vaccination, days, median [IQR]	20.0 [10.0, 58.0]	90.0 [23.0, 157.0]	<0.001

*p-values from Chi-squared tests, Wilcoxon Rank Sum tests (continuous age and follow-up time) and Cochran-Armitage tests (ordinal age groups).

Figure S2 Vaccine efficacy against asymptomatic/unknown infection by interval between first and second dose after, A) SD/SD or LD/SD, B) SD/SD, C) LD/SD administration

A) SD/SD or LD/SD

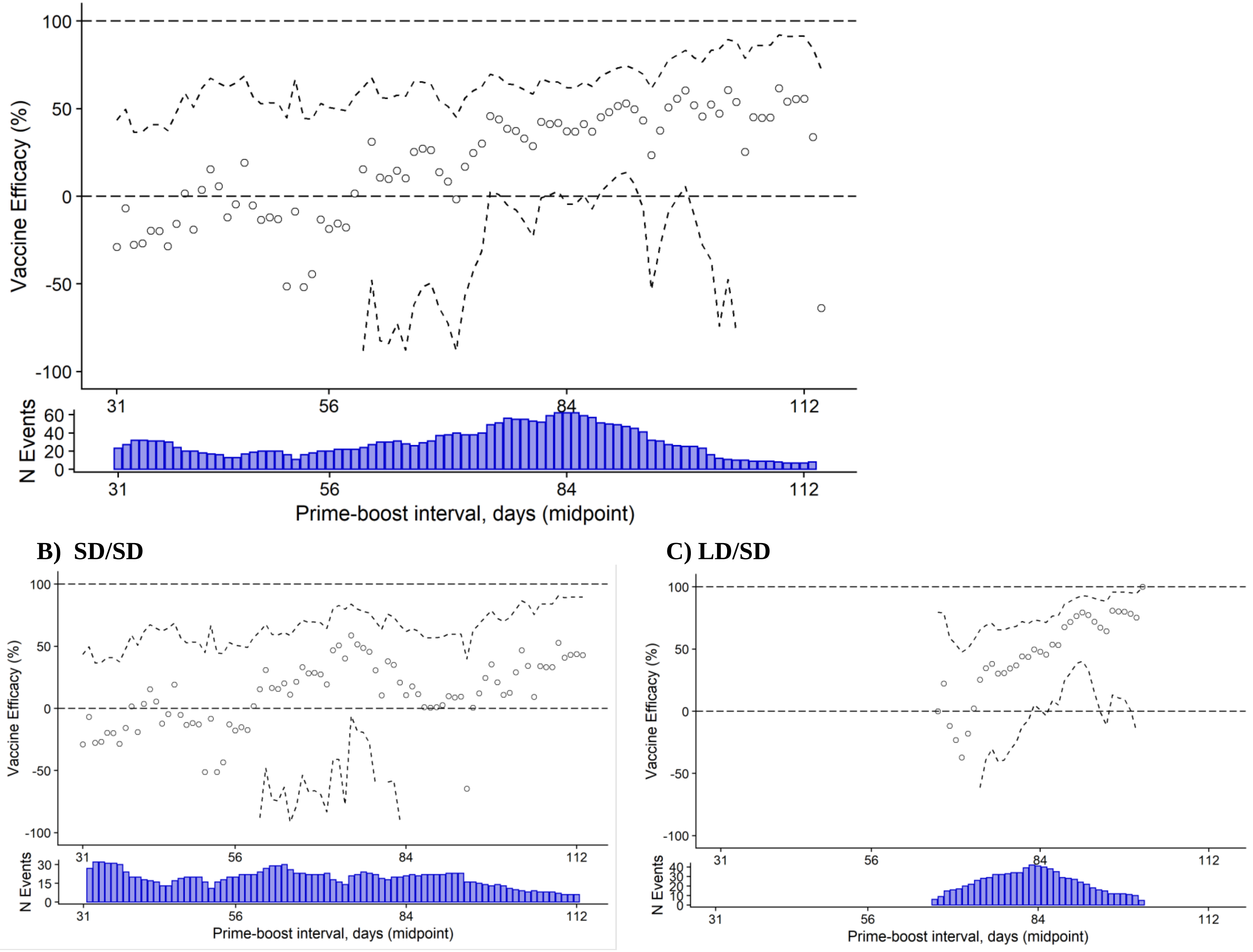

Each dot represents one estimate of vaccine efficacy in a subset of participants who received two doses of vaccine with a gap between first and second dose within a 20 day range. The x axis shows the midpoint of the 20 day range for dosing. Dotted lines show 95% confidence intervals for each dot point estimate of VE. Solid line shows a cubic spline smooth function for VE.

Table S5 SARS-CoV-2 anti-spike IgG responses by multiplex immunoassay at 28 days after a second dose, by prime-boost interval

Age group	Study	Prime-boost interval	ChAdOx1 nCoV-19				Control			
			N	Median [IQR]	GMT (95% CI)	GMR (95% CI)	N	Median [IQR]	GMT (95% CI)	GMR (95% CI)
18-55 years	Overall (SD/SD)	<6 weeks	332	23173 [11665, 43633]	21966 (19753, 24427)	ref	318	62 [16, 135]	74 (63, 86)	ref
		6-8 weeks	287	30743 [18448, 52217]	29294 (26178, 32781)	1.33 (1.16, 1.54)	196	56 [16, 169]	71 (58, 86)	0.96 (0.75, 1.24)
		9-11 weeks	333	36044 [22647, 60646]	36946 (34010, 40135)	1.68 (1.46, 1.93)	233	51 [16, 101]	62 (51, 75)	0.84 (0.66, 1.06)
		≥12 weeks	289	51291 [28597, 93521]	50918 (45842, 56556)	2.32 (2.01, 2.68)	173	53 [16, 98]	56 (47, 66)	0.76 (0.58, 0.98)
	Overall (SD/SD and LD/SD)	<6 weeks	335	23241 [11750, 43560]	21967 (19772, 24406)	ref	323	62 [16, 135]	74 (63, 86)	ref
		6-8 weeks	288	30719 [18263, 52216]	29244 (26142, 32714)	1.33 (1.15, 1.54)	196	56 [16, 169]	71 (58, 86)	0.97 (0.76, 1.23)
		9-11 weeks	460	36468 [22276, 62523]	36177 (33417, 39165)	1.65 (1.44, 1.88)	324	48 [16, 95]	57 (49, 67)	0.78 (0.63, 0.96)
		≥12 weeks	445	51005 [29002, 88849]	48961 (44880, 53413)	2.23 (1.95, 2.54)	271	46 [16, 87]	51 (44, 58)	0.69 (0.55, 0.85)
≥56 years	Overall (SD/SD)	<6 weeks	282	20328 [10534, 35685]	18859 (16900, 21046)	ref	273	34 [16, 78]	40 (35, 46)	ref
		6-8 weeks	66	23659 [13620, 37338]	23809 (20039, 28288)	1.26 (0.99, 1.61)	66	39 [16, 79]	43 (33, 56)	1.07 (0.80, 1.43)
		9-11 weeks	4	26374 [22367, 28102]	23432 (14885, 36887)	1.24 (0.51, 3.01)	3	16 [16, 16]	16 (16, 16)	0.41 (0.12, 1.41)
	Overall (SD/SD and LD/SD)	<6 weeks	282	20328 [10534, 35685]	18859 (16900, 21046)	ref	273	34 [16, 78]	40 (35, 46)	ref
		6-8 weeks	66	23659 [13620, 37338]	23809 (20039, 28288)	1.26 (0.99, 1.61)	66	39 [16, 79]	43 (33, 56)	1.07 (0.80, 1.43)
		9-11 weeks	4	26374 [22367, 28102]	23432 (14885, 36887)	1.24 (0.51, 3.01)	3	16 [16, 16]	16 (16, 16)	0.41 (0.12, 0.41)
		≥12 weeks	1	39443	39443	2.09 (0.36, 12.15)	0	-	-	-
**Country Level Estimates**										
18-55 years	COV001 (UK) SD/SD	≥12 weeks	92	71270 [43306, 129621]	76070 (62987, 91872)		52	47 [16, 120]	52 (37, 73)	
	COV002 (UK) SD/SD	<6 weeks	33	12828 [8605, 31356]	13463 (9248, 19599)		24	16 [16, 50]	38 (21, 67)	
		6-8 weeks	162	30849 [18421, 48794]	28832 (25094, 33128)		81	38 [16, 113]	52 (39, 70)	
		9-11 weeks	248	36649 [22504, 63378]	37694 (34394, 41310)		156	48 [16, 96]	54 (44, 66)	
		≥12 weeks	191	43938 [24792, 76664]	42401 (37674, 47722)		114	57 [16, 92]	57 (46, 69)	
	COV002 (UK) LD/SD	6-8 weeks	1	17871	17871		0	-	-	
		9-11 weeks	127	39670 [21068, 66338]	34236 (28304, 41412)		91	39 [16, 86]	47 (37, 60)	
		≥12 weeks	156	49584 [31122, 81163]	45533 (38988, 53177)		98	39 [16, 72]	43 (35, 52)	
	COV003 (Brazil) SD/SD	<6 weeks	211	21006 [11638, 37206]	20542 (18162, 23233)		203	70 [34, 154]	81 (67, 98)	
		6-8 weeks	116	27187 [16548, 55060]	28649 (23497, 34930)		113	76 [40, 206]	90 (69, 117)	
		9-11 weeks	85	31871 [22997, 53258]	34847 (28912, 42000)		77	60 [16, 102]	81 (54, 122)	
		≥12 weeks	6	56933 [27600, 92280]	36630 (8849, 151637)		7	95 [16, 100]	73 (14, 394)	
	COV005 (South Africa) SD/SD	<6 weeks	88	36010 [19167, 62937]	30994 (24921, 38547)		91	54 [16, 112]	71 (52, 99)	
		6-8 weeks	9	56458 [39228, 63942]	51943 (34155, 78995)		2	28 [22, 33]	25 (0, 5993)	

	COV005 (South Africa) LD/SD	<6 weeks	3	24956 [19684, 27528]	22121 (8548, 57250)		5	115 [16, 172]	65 (13, 319)	
≥56 years	COV002 (UK) SD/SD	<6 weeks	203	19806 [11647, 33898]	19182 (17056, 21572)		202	25 [16, 72]	38 (33, 45)	
		6-8 weeks	59	22735 [12978, 35102]	22696 (18950, 27183)		64	35 [16, 72]	41 (32, 52)	
		9-11 weeks	4	26374 [22367, 28102]	23432 (14885, 36887)		3	16 [16, 16]	16 (16, 16)	
	COV002 (UK) LD/SD	≥12 weeks	1	39443	39443		0	-	-	
	COV003 (Brazil) SD/SD	<6 weeks	75	21027 [8645, 38450]	17309 (13389, 22377)		67	37 [16, 82]	43 (33, 56)	
		6-8 weeks	6	31811 [22330, 38513]	32632 (15397, 69159)		2	269 [236, 302]	261 (11, 6286)	
	COV005 (South Africa) SD/SD	<6 weeks	4	49068 [38190, 79322]	39899 (4506, 353262)		4	345 [83, 618]	164 (10, 2691)	
		6-8 weeks	1	60514	60514		0	-	-	

Figure S3 Neutralising antibody 28 days after a booster dose, measured in pseudovirus assay (Monogram IC50)

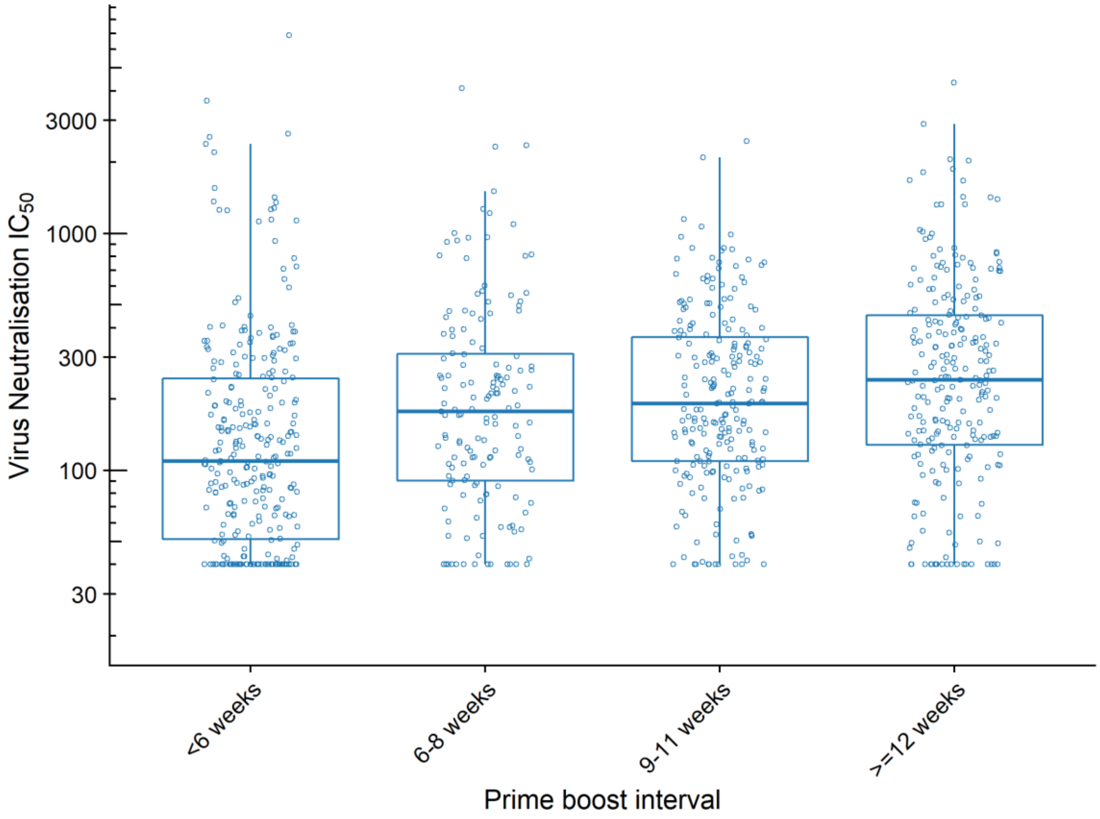

Participants who were PCR+ prior to the blood sample taken at day 28 post boost were removed from the analyses. Analysis includes SD/SD and LD/SD recipients.

Table S6 Neutralising antibody 28 days after a booster dose, measured in pseudovirus assay (Monogram IC50)

Prime-boost interval	N	Median [IQR]	GMT (95% CI)	GMR (95% CI)	P value
<6 weeks	279	109 [51, 246]	127.8 (113.1, 144.3)	ref	<0.0001
6-8 weeks	151	177 [90, 311]	182.7 (156.0, 214.1)	1.43 (1.18, 1.74)	
9-11 weeks	224	191 [109, 367]	198.1 (176.8, 221.9)	1.55 (1.31, 1.84)	
≥12 weeks	239	241 [128,452]	237.0 (208.5, 269.4)	1.85 (1.57, 2.20)	

Figure S4 Kaplan-Meier cumulative incidence of primary symptomatic PCR+ cases after booster dose, in the primary efficacy cohort

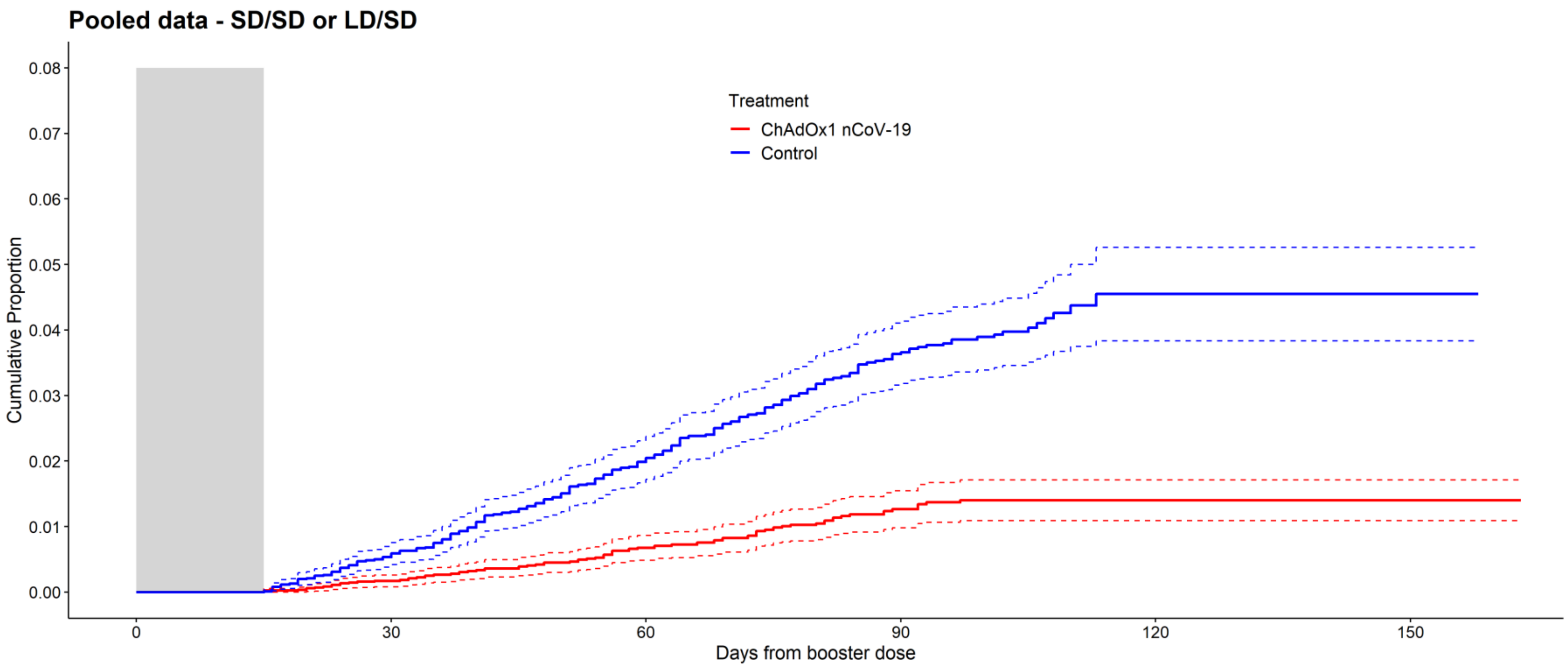

Number at risk (number censored)

	0	30	60	90	120	150
ChAdOx1 nCoV-19 (red)	8597 (0)	7683 (954)	6411 (2195)	3750 (4769)	115 (8398)	17 (8496)
Control (blue)	8581 (0)	7633 (956)	6359 (2142)	3632 (4719)	93 (8240)	1 (8332)

Figure S5 Kaplan-Meier cumulative incidence of primary symptomatic PCR+ cases after booster dose in SD/SD cohorts, by country*

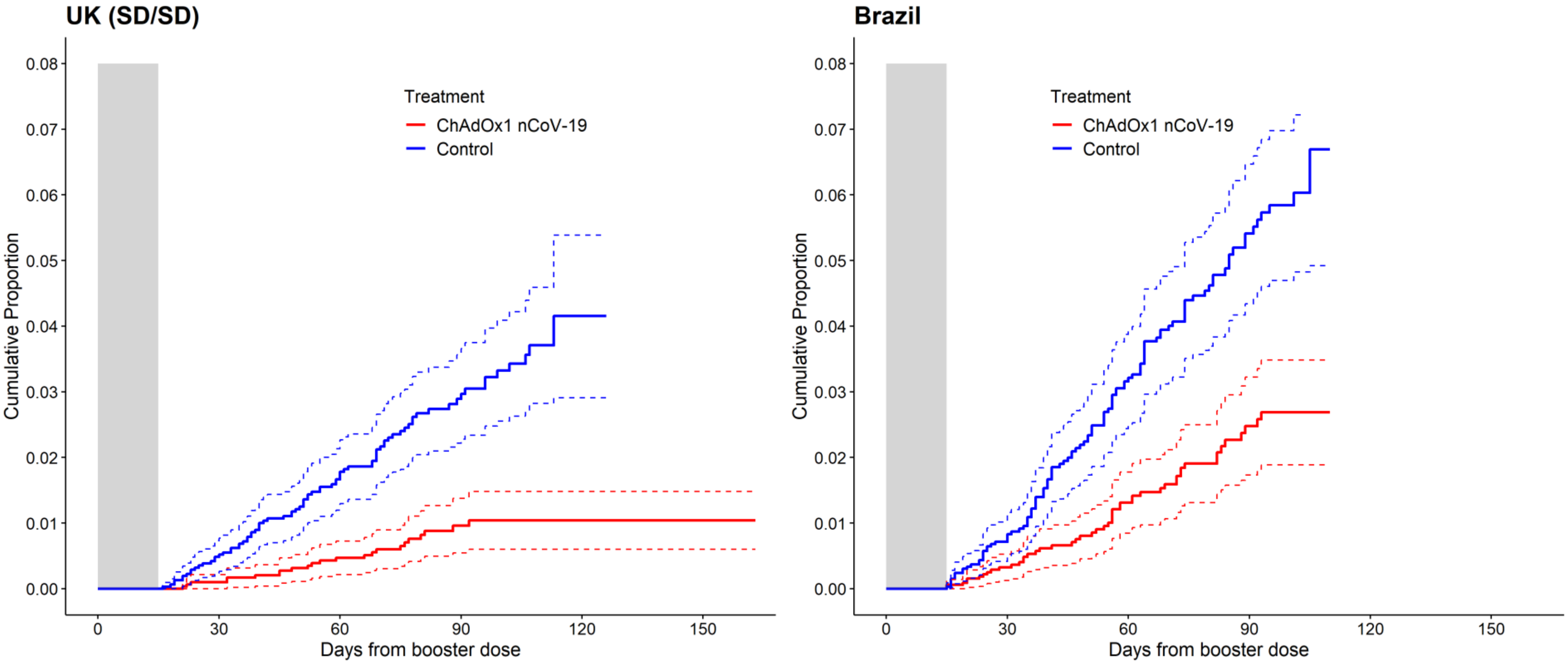

UK (SD/SD) — Number at risk (number censored)

	0	30	60	90	120	150
ChAdOx1 nCoV-19	3048 (0)	2932 (131)	2483 (576)	1252 (1774)	21 (3004)	17 (3008)
Control	3136 (0)	3009 (129)	2537 (586)	1252 (1812)	3 (3051)	0 (3054)

Brazil — Number at risk (number censored)

	0	30	60	90	120	150
ChAdOx1 nCoV-19	3414 (0)	2635 (804)	1895 (1520)	931 (2436)	0 (3365)	0 (3365)
Control	3339 (0)	2548 (804)	1846 (1445)	890 (2343)	0 (3227)	0 (3227)

*Kaplan-Meier curves for South Africa study not presented due to small number of cases contributing to the primary efficacy analysis.

Table S7 Serious Adverse Events by System Organ Class and Preferred Term

System Organ Class[a]<br>Preferred Term<br>(MedDRA version 23.1)	ChAdOx1 nCoV-19 (N = 12282)<br>Number (%) of Participants[b]	ChAdOx1 nCoV-19 (N = 12282)<br>Number of Events	Control (N = 11962)<br>Number (%) of Participants[b]	Control (N = 11962)<br>Number of Events
**Participants with any SAE**	**108 (0.9)**	**117**	**127 (1.1)**	**131**
**Infections and infestations**	**23 (0.2)**	**26**	**41 (0.3)**	**41**
Acute sinusitis	0	0	1 (<0.1)	1
Appendicitis	6 (<0.1)	6	7 (0.1)	7
Arthritis bacterial	1 (<0.1)	1	0	0
Biliary sepsis	1 (<0.1)	1	0	0
COVID-19	2 (<0.1)	2	17 (0.1)	17
COVID-19 pneumonia	0	0	4 (<0.1)	4
Campylobacter colitis	1 (<0.1)	1	0	0
Cellulitis	1 (<0.1)	1	0	0
Diverticulitis	3 (<0.1)	3	0	0
Gastroenteritis	0	0	1 (<0.1)	1
Haematoma infection	0	0	1 (<0.1)	1
Hepatitis infectious mononucleosis	1 (<0.1)	1	0	0
Labyrinthitis	0	0	1 (<0.1)	1
Myelitis	0	0	1 (<0.1)	1
Otitis externa	1 (<0.1)	1	0	0
Peritonitis	0	0	1 (<0.1)	1
Pilonidal cyst	0	0	2 (<0.1)	2
Pneumonia bacterial	1 (<0.1)	1	0	0
Pulmonary tuberculosis	1 (<0.1)	1	0	0
Pyelonephritis	2 (<0.1)	2	0	0
Pyelonephritis acute	1 (<0.1)	1	0	0
Respiratory tract infection fungal	1 (<0.1)	1	0	0
Sepsis	0	0	2 (<0.1)	2
Staphylococcal bacteraemia	1 (<0.1)	1	0	0
Subcutaneous abscess	0	0	1 (<0.1)	1
Tonsillitis	0	0	1 (<0.1)	1
Tuberculosis	1 (<0.1)	1	0	0
Tuberculosis of eye	0	0	1 (<0.1)	1
Urinary tract infection	1 (<0.1)	1	0	0
**Neoplasms benign, malignant and unspecified (incl cysts and polyps)**	**6 (<0.1)**	**6**	**11 (0.1)**	**11**
Benign soft tissue neoplasm	1 (<0.1)	1	0	0
Breast cancer	1 (<0.1)	1	0	0
Colorectal cancer metastatic	0	0	1 (<0.1)	1
Haematological malignancy	0	0	1 (<0.1)	1

Intracranial haemangioma	0	0	1 (<0.1)	1
Invasive ductal breast carcinoma	1 (<0.1)	1	0	0
Lip squamous cell carcinoma	0	0	1 (<0.1)	1
Liposarcoma	0	0	1 (<0.1)	1
Lymphoplasmacytoid lymphoma/immunocytoma	0	0	1 (<0.1)	1
Malignant melanoma	1 (<0.1)	1	1 (<0.1)	1
Melanoma recurrent	0	0	1 (<0.1)	1
Metastatic neoplasm	1 (<0.1)	1	0	0
Ovarian germ cell teratoma benign	0	0	1 (<0.1)	1
Papillary renal cell carcinoma	1 (<0.1)	1	0	0
Papillary thyroid cancer	0	0	1 (<0.1)	1
Uterine leiomyoma	0	0	1 (<0.1)	1
**Blood and lymphatic system disorders**	**0**	**0**	**1 (<0.1)**	**1**
Autoimmune haemolytic anaemia	0	0	1 (<0.1)	1
**Immune system disorders**	**0**	**0**	**1 (<0.1)**	**1**
Allergy to arthropod sting	0	0	1 (<0.1)	1
**Metabolism and nutrition disorders**	**0**	**0**	**1 (<0.1)**	**1**
Diabetic ketoacidosis	0	0	1 (<0.1)	1
**Psychiatric disorders**	**3 (<0.1)**	**3**	**1 (<0.1)**	**1**
Depression suicidal	1 (<0.1)	1	0	0
Substance abuse	1 (<0.1)	1	0	0
Substance-induced psychotic disorder	1 (<0.1)	1	1 (<0.1)	1
**Nervous system disorders**	**10 (0.1)**	**10**	**8 (0.1)**	**8**
Facial spasm	1 (<0.1)	1	0	0
Hemiplegic migraine	0	0	1 (<0.1)	1
Ischaemic stroke	1 (<0.1)	1	0	0
Migraine	1 (<0.1)	1	0	0
Migraine with aura	1 (<0.1)	1	0	0
Multiple sclerosis	1 (<0.1)	1	0	0
Myelitis transverse	1 (<0.1)	1	0	0
Presyncope	1 (<0.1)	1	0	0
Serotonin syndrome	1 (<0.1)	1	0	0
Spinal cord compression	1 (<0.1)	1	0	0
Subarachnoid haemorrhage	0	0	2 (<0.1)	2
Syncope	0	0	2 (<0.1)	2
Transient ischaemic attack	0	0	3 (<0.1)	3
VIth nerve paralysis	1 (<0.1)	1	0	0
**Eye disorders**	**2 (<0.1)**	**2**	**0**	**0**
Retinal detachment	1 (<0.1)	1	0	0
Retinal tear	1 (<0.1)	1	0	0

**Cardiac disorders**	**7 (0.1)**	**7**	**10 (0.1)**	**10**
Acute coronary syndrome	0	0	1 (<0.1)	1
Acute myocardial infarction	0	0	2 (<0.1)	2
Angina pectoris	2 (<0.1)	2	0	0
Angina unstable	1 (<0.1)	1	0	0
Atrial flutter	1 (<0.1)	1	0	0
Atrioventricular block complete	1 (<0.1)	1	0	0
Atrioventricular block second degree	0	0	1 (<0.1)	1
Cardiac failure congestive	0	0	1 (<0.1)	1
Myocardial infarction	1 (<0.1)	1	1 (<0.1)	1
Pericarditis	1 (<0.1)	1	2 (<0.1)	2
Supraventricular tachycardia	0	0	1 (<0.1)	1
Ventricular extrasystoles	0	0	1 (<0.1)	1
**Vascular disorders**	**0**	**0**	**2 (<0.1)**	**2**
Deep vein thrombosis	0	0	1 (<0.1)	1
Peripheral ischaemia	0	0	1 (<0.1)	1
**Respiratory, thoracic and mediastinal disorders**	**1 (<0.1)**	**1**	**1 (<0.1)**	**1**
Dyspnoea	1 (<0.1)	1	0	0
Pulmonary embolism	0	0	1 (<0.1)	1
**Gastrointestinal disorders**	**15 (0.1)**	**16**	**13 (0.1)**	**13**
Abdominal pain	2 (<0.1)	2	1 (<0.1)	1
Anal incontinence	0	0	1 (<0.1)	1
Constipation	0	0	1 (<0.1)	1
Diarrhoea	1 (<0.1)	1	0	0
Dyspepsia	1 (<0.1)	1	0	0
Enteritis	0	0	1 (<0.1)	1
Epiploic appendagitis	0	0	1 (<0.1)	1
Gastritis	1 (<0.1)	1	1 (<0.1)	1
Gastrointestinal haemorrhage	1 (<0.1)	1	0	0
Gastrooesophageal reflux disease	0	0	1 (<0.1)	1
Haematemesis	0	0	1 (<0.1)	1
Incarcerated inguinal hernia	1 (<0.1)	1	0	0
Intussusception	1 (<0.1)	1	0	0
Mesenteric vein thrombosis	0	0	1 (<0.1)	1
Oesophageal spasm	1 (<0.1)	1	0	0
Pancreatitis	3 (<0.1)	3	0	0
Pancreatitis acute	1 (<0.1)	1	1 (<0.1)	1
Small intestinal obstruction	0	0	2 (<0.1)	2
Volvulus	2 (<0.1)	2	0	0
Vomiting	1 (<0.1)	1	1 (<0.1)	1
**Hepatobiliary disorders**	**0**	**0**	**3 (<0.1)**	**3**
Cholelithiasis	0	0	2 (<0.1)	2

Hepatic function abnormal	0	0	1 (<0.1)	1
**Skin and subcutaneous tissue disorders**	**0**	**0**	**1 (<0.1)**	**1**
Angioedema	0	0	1 (<0.1)	1
**Musculoskeletal and connective tissue disorders**	**5 (<0.1)**	**5**	**3 (<0.1)**	**3**
Arthritis reactive	0	0	1 (<0.1)	1
Back pain	1 (<0.1)	1	0	0
Costochondritis	0	0	1 (<0.1)	1
Intervertebral disc protrusion	2 (<0.1)	2	0	0
Polymyalgia rheumatica	1 (<0.1)	1	0	0
Rhabdomyolysis	0	0	1 (<0.1)	1
Spinal pain	1 (<0.1)	1	0	0
**Renal and urinary disorders**	**5 (<0.1)**	**5**	**7 (0.1)**	**7**
Acute kidney injury	1 (<0.1)	1	0	0
Calculus urethral	0	0	1 (<0.1)	1
Calculus urinary	2 (<0.1)	2	2 (<0.1)	2
Nephrolithiasis	1 (<0.1)	1	2 (<0.1)	2
Renal colic	1 (<0.1)	1	0	0
Ureterolithiasis	0	0	2 (<0.1)	2
**Pregnancy, puerperium and perinatal conditions**	**4 (<0.1)**	**4**	**2 (<0.1)**	**2**
Abortion incomplete	0	0	1 (<0.1)	1
Abortion spontaneous	2 (<0.1)	2	1 (<0.1)	1
Hyperemesis gravidarum	1 (<0.1)	1	0	0
Pregnancy	1 (<0.1)	1	0	0
**Reproductive system and breast disorders**	**8 (0.1)**	**9**	**2 (<0.1)**	**2**
Adnexal torsion	2 (<0.1)	2	0	0
Endometriosis	2 (<0.1)	2	0	0
Genital pain	1 (<0.1)	1	0	0
Haemorrhagic ovarian cyst	2 (<0.1)	2	0	0
Pelvic pain	0	0	1 (<0.1)	1
Uterine haemorrhage	1 (<0.1)	1	1 (<0.1)	1
Vaginal haemorrhage	1 (<0.1)	1	0	0
**Congenital, familial and genetic disorders**	**0**	**0**	**1 (<0.1)**	**1**
Arnold-Chiari malformation	0	0	1 (<0.1)	1
**General disorders and administration site conditions**	**4 (<0.1)**	**4**	**2 (<0.1)**	**2**
Chest pain	1 (<0.1)	1	0	0
Influenza like illness	1 (<0.1)	1	0	0
Mass	0	0	1 (<0.1)	1

Non-cardiac chest pain	0	0	1 (<0.1)	1
Pyrexia	2 (<0.1)	2	0	0
**Investigations**	**3 (<0.1)**	**3**	**1 (<0.1)**	**1**
C-reactive protein increased	1 (<0.1)	1	1 (<0.1)	1
Liver function test abnormal	1 (<0.1)	1	0	0
Oxygen saturation decreased	1 (<0.1)	1	0	0
**Injury, poisoning and procedural complications**	**15 (0.1)**	**15**	**17 (0.1)**	**18**
Animal bite	0	0	1 (<0.1)	1
Cervical vertebral fracture	1 (<0.1)	1	0	0
Clavicle fracture	1 (<0.1)	1	0	0
Craniocerebral injury	1 (<0.1)	1	1 (<0.1)	1
Fibula fracture	1 (<0.1)	1	0	0
Gun shot wound	1 (<0.1)	1	0	0
Injury	0	0	1 (<0.1)	1
Intentional overdose	0	0	2 (<0.1)	2
Joint dislocation	1 (<0.1)	1	1 (<0.1)	1
Limb injury	1 (<0.1)	1	0	0
Lower limb fracture	0	0	1 (<0.1)	2
Meniscus injury	2 (<0.1)	2	0	0
Overdose	0	0	1 (<0.1)	1
Post procedural complication	0	0	1 (<0.1)	1
Procedural nausea	1 (<0.1)	1	0	0
Radius fracture	0	0	1 (<0.1)	1
Road traffic accident	0	0	2 (<0.1)	2
Scapula fracture	1 (<0.1)	1	0	0
Synovial rupture	1 (<0.1)	1	0	0
Tendon injury	1 (<0.1)	1	0	0
Tendon rupture	1 (<0.1)	1	0	0
Thermal burn	1 (<0.1)	1	0	0
Traumatic lung injury	0	0	1 (<0.1)	1
Upper limb fracture	0	0	2 (<0.1)	2
Wrist fracture	0	0	2 (<0.1)	2
**Social circumstances**	**0**	**0**	**1 (<0.1)**	**1**
Homicide	0	0	1 (<0.1)	1

Table S8 Unsolicited Adverse Events by System Organ Class and Preferred Term (severity grade 3 or higher)

System Organ Class<br>Preferred Term		Number (%) of Participants<br>ChAdOx1 nCoV-19<br>(N = 12282)	Control<br>(N = 11962)
**Infections and infestations**			
Acute sinusitis	Grade 4	0	1 (<0.1)
Appendicitis	Grade 3	1 (<0.1)	0
	Grade 4	3 (<0.1)	3 (<0.1)
Arthritis bacterial	Grade 3	1 (<0.1)	0
Biliary sepsis	Grade 3	1 (<0.1)	0
COVID-19	Grade 3	3 (<0.1)	4 (<0.1)
	Grade 4	2 (<0.1)	6 (0.1)
COVID-19 pneumonia	Grade 4	0	1 (<0.1)
Cellulitis	Grade 3	1 (<0.1)	1 (<0.1)
Diverticulitis	Grade 3	2 (<0.1)	0
Ear infection	Grade 3	0	1 (<0.1)
Escherichia infection	Grade 3	1 (<0.1)	0
Gastroenteritis	Grade 3	1 (<0.1)	0
Genital herpes	Grade 3	0	1 (<0.1)
Gingivitis	Grade 3	1 (<0.1)	0
Helicobacter infection	Grade 3	2 (<0.1)	0
Herpes zoster	Grade 3	0	3 (<0.1)
Labyrinthitis	Grade 4	1 (<0.1)	0
Large intestine infection	Grade 4	1 (<0.1)	0
Nasopharyngitis	Grade 3	3 (<0.1)	4 (<0.1)
Otitis externa	Grade 4	1 (<0.1)	0
Pharyngitis	Grade 3	0	1 (<0.1)
Pharyngitis bacterial	Grade 3	1 (<0.1)	0
Pneumonia bacterial	Grade 4	1 (<0.1)	0
Pyelonephritis	Grade 3	2 (<0.1)	1 (<0.1)
Rhinitis	Grade 3	0	1 (<0.1)
Sepsis	Grade 4	0	1 (<0.1)
Septic shock	Grade 4	0	1 (<0.1)
Suspected COVID-19	Grade 3	1 (<0.1)	0
Tonsillitis	Grade 3	0	3 (<0.1)
Tooth infection	Grade 3	1 (<0.1)	1 (<0.1)
Upper respiratory tract infection	Grade 3	3 (<0.1)	1 (<0.1)
Urinary tract infection	Grade 3	0	1 (<0.1)
Viral pharyngitis	Grade 3	0	1 (<0.1)
Viral rash	Grade 3	1 (<0.1)	0
Wound infection	Grade 3	1 (<0.1)	0
**Neoplasms benign, malignant and unspecified (incl cysts and polyps)**			
Basal cell carcinoma	Grade 3	0	1 (<0.1)
Benign soft tissue neoplasm	Grade 4	1 (<0.1)	0

Colorectal cancer metastatic	Grade 4	0	1 (<0.1)
Haematological malignancy	Grade 4	0	1 (<0.1)
Metastases to liver	Grade 4	0	1 (<0.1)
Metastases to lung	Grade 4	0	1 (<0.1)
Ovarian adenoma	Grade 3	1 (<0.1)	0
Papillary thyroid cancer	Grade 3	0	1 (<0.1)
**Blood and lymphatic system disorders**			
	Grade 4	0	1 (<0.1)
Anaemia	Grade 3	0	1 (<0.1)
Iron deficiency anaemia	Grade 3	0	2 (<0.1)
Lymphadenopathy	Grade 3	2 (<0.1)	1 (<0.1)
	Grade 4	0	1 (<0.1)
Neutropenia	Grade 3	0	2 (<0.1)
**Immune system disorders**			
Allergy to chemicals	Grade 3	0	1 (<0.1)
Metabolism and nutrition disorders	Grade 3	3 (<0.1)	1 (<0.1)
	Grade 4	0	1 (<0.1)
Decreased appetite	Grade 3	1 (<0.1)	0
Dehydration	Grade 3	1 (<0.1)	1 (<0.1)
Diabetic ketoacidosis	Grade 4	0	1 (<0.1)
Hypokalaemia	Grade 3	1 (<0.1)	0
**Psychiatric disorders**			
Anxiety	Grade 3	2 (<0.1)	0
Depression	Grade 3	1 (<0.1)	0
Depression suicidal	Grade 4	1 (<0.1)	0
Psychotic disorder	Grade 3	0	1 (<0.1)
**Nervous system disorders**			
Ageusia	Grade 3	6 (<0.1)	5 (<0.1)
Anosmia	Grade 3	6 (<0.1)	5 (<0.1)
Balance disorder	Grade 4	0	1 (<0.1)
Dizziness	Grade 3	4 (<0.1)	2 (<0.1)
	Grade 4	0	1 (<0.1)
Dysaesthesia	Grade 3	2 (<0.1)	0
Dysarthria	Grade 4	0	1 (<0.1)
Facial paralysis	Grade 3	2 (<0.1)	0
Facial spasm	Grade 3	1 (<0.1)	0
Headache	Grade 3	31 (0.3)	26 (0.2)
	Grade 4	0	3 (<0.1)
Hypersomnia	Grade 3	1 (<0.1)	0
Migraine	Grade 3	1 (<0.1)	6 (0.1)
	Grade 4	1 (<0.1)	0
Multiple sclerosis	Grade 3	1 (<0.1)	0
Myelitis transverse	Grade 3	1 (<0.1)	0
Nystagmus	Grade 3	1 (<0.1)	0
Paraesthesia	Grade 3	1 (<0.1)	0
Parosmia	Grade 3	0	1 (<0.1)

Post herpetic neuralgia	Grade 3	1 (<0.1)	0
Presyncope	Grade 3	1 (<0.1)	1 (<0.1)
Sensory loss	Grade 3	0	1 (<0.1)
Serotonin syndrome	Grade 4	1 (<0.1)	0
Syncope	Grade 3	0	1 (<0.1)
Taste disorder	Grade 3	0	1 (<0.1)
Tonic convulsion	Grade 3	1 (<0.1)	0
Visual field defect	Grade 3	1 (<0.1)	0
**Eye disorders**			
Blindness	Grade 3	1 (<0.1)	0
Blindness unilateral	Grade 4	1 (<0.1)	0
Conjunctivitis allergic	Grade 3	0	1 (<0.1)
Eye pain	Grade 3	1 (<0.1)	0
Ocular hyperaemia	Grade 3	0	2 (<0.1)
Periorbital oedema	Grade 3	0	1 (<0.1)
Photophobia	Grade 3	2 (<0.1)	0
Retinal detachment	Grade 3	1 (<0.1)	1 (<0.1)
Vision blurred	Grade 3	0	1 (<0.1)
Vitreous detachment	Grade 3	1 (<0.1)	0
Vitreous floaters	Grade 3	1 (<0.1)	0
**Ear and labyrinth disorders**			
Auricular chondritis	Grade 4	1 (<0.1)	0
Ear pain	Grade 3	0	1 (<0.1)
Tinnitus	Grade 3	0	1 (<0.1)
Vertigo	Grade 3	2 (<0.1)	1 (<0.1)
**Cardiac disorders**			
Angina pectoris	Grade 3	1 (<0.1)	1 (<0.1)
	Grade 4	1 (<0.1)	0
Angina unstable	Grade 4	1 (<0.1)	0
Atrioventricular block complete	Grade 3	1 (<0.1)	0
Bradyarrhythmia	Grade 4	0	1 (<0.1)
Cardiac failure congestive	Grade 4	0	1 (<0.1)
Coronary artery occlusion	Grade 3	0	1 (<0.1)
	Grade 4	1 (<0.1)	0
Myocardial infarction	Grade 3	0	1 (<0.1)
Palpitations	Grade 3	0	3 (<0.1)
Pericarditis	Grade 3	0	1 (<0.1)
Supraventricular tachycardia	Grade 3	0	1 (<0.1)
Tachycardia	Grade 3	2 (<0.1)	1 (<0.1)
Ventricular extrasystoles	Grade 3	0	1 (<0.1)
**Vascular disorders**			
Hypertension	Grade 3	2 (<0.1)	1 (<0.1)
Hypotension	Grade 3	0	1 (<0.1)
Orthostatic hypotension	Grade 3	1 (<0.1)	0
**Respiratory, thoracic and mediastinal disorders**			
Asthma	Grade 3	1 (<0.1)	1 (<0.1)

Cough	Grade 3	0	5 (<0.1)
Dyspnoea	Grade 3	3 (<0.1)	3 (<0.1)
Dyspnoea exertional	Grade 4	1 (<0.1)	0
Lung opacity	Grade 3	0	1 (<0.1)
Nasal turbinate hypertrophy	Grade 4	0	1 (<0.1)
Oropharyngeal pain	Grade 3	3 (<0.1)	4 (<0.1)
Rhinorrhoea	Grade 3	1 (<0.1)	2 (<0.1)
Sneezing	Grade 3	0	1 (<0.1)
	Grade 4	1 (<0.1)	0
**Gastrointestinal disorders**			
Abdominal discomfort	Grade 3	1 (<0.1)	0
Abdominal pain	Grade 3	3 (<0.1)	3 (<0.1)
	Grade 4	1 (<0.1)	0
Abdominal pain lower	Grade 3	2 (<0.1)	1 (<0.1)
Abdominal pain upper	Grade 3	1 (<0.1)	0
	Grade 4	0	1 (<0.1)
Anal incontinence	Grade 3	0	1 (<0.1)
Colitis	Grade 3	1 (<0.1)	0
Constipation	Grade 3	1 (<0.1)	1 (<0.1)
Crohn's disease	Grade 3	0	1 (<0.1)
Diarrhoea	Grade 3	2 (<0.1)	8 (0.1)
	Grade 4	2 (<0.1)	0
Discoloured vomit	Grade 3	1 (<0.1)	0
Dyspepsia	Grade 3	0	1 (<0.1)
Gastric polyps	Grade 4	1 (<0.1)	0
Gastritis	Grade 3	3 (<0.1)	0
Gastrointestinal haemorrhage	Grade 4	1 (<0.1)	0
Gastrointestinal inflammation	Grade 4	0	1 (<0.1)
Gastrooesophageal reflux disease	Grade 3	1 (<0.1)	0
Haematemesis	Grade 3	0	1 (<0.1)
Hiatus hernia	Grade 4	1 (<0.1)	0
Incarcerated inguinal hernia	Grade 4	1 (<0.1)	0
Lip oedema	Grade 3	0	1 (<0.1)
Lip swelling	Grade 3	1 (<0.1)	0
Melaena	Grade 4	1 (<0.1)	0
Nausea	Grade 3	11 (0.1)	3 (<0.1)
	Grade 4	1 (<0.1)	2 (<0.1)
Odynophagia	Grade 3	1 (<0.1)	0
Oesophageal spasm	Grade 4	1 (<0.1)	0
Oral discomfort	Grade 3	1 (<0.1)	0
Pancreatitis	Grade 4	1 (<0.1)	1 (<0.1)
Proctalgia	Grade 3	0	1 (<0.1)
Retching	Grade 3	1 (<0.1)	0
Toothache	Grade 3	0	2 (<0.1)
Volvulus	Grade 3	1 (<0.1)	0
	Grade 4	1 (<0.1)	0

Vomiting	Grade 3	6 (<0.1)	3 (<0.1)
	Grade 4	1 (<0.1)	0
**Hepatobiliary disorders**			
Cholecystitis	Grade 4	1 (<0.1)	0
Cholelithiasis	Grade 3	2 (<0.1)	0
	Grade 4	0	1 (<0.1)
**Skin and subcutaneous tissue disorders**			
Angioedema	Grade 3	0	1 (<0.1)
	Grade 4	0	1 (<0.1)
Erythema	Grade 3	1 (<0.1)	1 (<0.1)
Hyperhidrosis	Grade 3	1 (<0.1)	1 (<0.1)
Night sweats	Grade 3	0	1 (<0.1)
Pruritus	Grade 3	1 (<0.1)	1 (<0.1)
Rash	Grade 3	0	1 (<0.1)
Rash papular	Grade 3	1 (<0.1)	0
Skin burning sensation	Grade 3	0	1 (<0.1)
Skin ulcer	Grade 3	1 (<0.1)	0
Urticaria	Grade 3	1 (<0.1)	0
**Musculoskeletal and connective tissue disorders**			
Arthralgia	Grade 3	6 (<0.1)	6 (0.1)
	Grade 4	1 (<0.1)	0
Arthropathy	Grade 3	0	1 (<0.1)
Back pain	Grade 3	6 (<0.1)	6 (0.1)
	Grade 4	0	2 (<0.1)
Costochondritis	Grade 3	1 (<0.1)	0
Intervertebral disc protrusion	Grade 3	4 (<0.1)	1 (<0.1)
	Grade 4	1 (<0.1)	0
Joint range of motion decreased	Grade 3	0	1 (<0.1)
Muscle spasms	Grade 3	0	1 (<0.1)
Musculoskeletal chest pain	Grade 3	1 (<0.1)	0
Myalgia	Grade 3	27 (0.2)	12 (0.1)
	Grade 4	4 (<0.1)	1 (<0.1)
Neck pain	Grade 3	3 (<0.1)	0
Osteoarthritis	Grade 4	1 (<0.1)	0
Pain in extremity	Grade 3	4 (<0.1)	1 (<0.1)
Pain in jaw	Grade 3	0	1 (<0.1)
Periarthritis	Grade 3	0	1 (<0.1)
Rotator cuff syndrome	Grade 3	1 (<0.1)	0
Somatic dysfunction	Grade 3	1 (<0.1)	0
Spinal osteoarthritis	Grade 3	1 (<0.1)	0
Spinal pain	Grade 3	1 (<0.1)	0
	Grade 4	1 (<0.1)	0
Spinal synovial cyst	Grade 4	1 (<0.1)	0
Spondylolysis	Grade 3	1 (<0.1)	0
**Renal and urinary disorders**			
Calculus urethral	Grade 4	0	1 (<0.1)

Dysuria	Grade 3	2 (<0.1)	1 (<0.1)
Micturition disorder	Grade 3	1 (<0.1)	0
Nephrolithiasis	Grade 3	1 (<0.1)	2 (<0.1)
	Grade 4	0	1 (<0.1)
Renal colic	Grade 3	1 (<0.1)	1 (<0.1)
	Grade 4	0	2 (<0.1)
Ureterolithiasis	Grade 4	0	1 (<0.1)
Urge incontinence	Grade 3	0	1 (<0.1)
**Reproductive system and breast disorders**			
Adnexal torsion	Grade 4	1 (<0.1)	0
Breast mass	Grade 3	1 (<0.1)	0
Breast pain	Grade 3	1 (<0.1)	0
Cervical dysplasia	Grade 3	0	1 (<0.1)
Endometriosis	Grade 4	1 (<0.1)	0
Haemorrhagic ovarian cyst	Grade 4	1 (<0.1)	0
Menorrhagia	Grade 3	0	1 (<0.1)
Ovarian cyst	Grade 4	1 (<0.1)	0
Ovarian cyst ruptured	Grade 3	0	1 (<0.1)
	Grade 4	1 (<0.1)	0
Pelvic haemorrhage	Grade 3	1 (<0.1)	0
Testicular pain	Grade 3	1 (<0.1)	0
Uterine haemorrhage	Grade 4	1 (<0.1)	0
Vaginal haemorrhage	Grade 4	1 (<0.1)	0
Vulvovaginal dryness	Grade 3	0	1 (<0.1)
**Congenital, familial and genetic disorders**			
Accessory spleen	Grade 3	1 (<0.1)	0
Phimosis	Grade 3	0	1 (<0.1)
**General disorders and administration site conditions**			
Asthenia	Grade 3	5 (<0.1)	3 (<0.1)
	Grade 4	1 (<0.1)	0
Chest discomfort	Grade 3	0	1 (<0.1)
Chest pain	Grade 3	3 (<0.1)	2 (<0.1)
	Grade 4	2 (<0.1)	1 (<0.1)
Chills	Grade 3	12 (0.1)	1 (<0.1)
	Grade 4	1 (<0.1)	1 (<0.1)
Fatigue	Grade 3	18 (0.1)	4 (<0.1)
	Grade 4	0	2 (<0.1)
Feeling hot	Grade 3	1 (<0.1)	0
Illness	Grade 3	0	1 (<0.1)
	Grade 4	0	1 (<0.1)
Influenza like illness	Grade 3	1 (<0.1)	0
Malaise	Grade 3	7 (0.1)	2 (<0.1)
	Grade 4	1 (<0.1)	2 (<0.1)
Oedema peripheral	Grade 3	1 (<0.1)	0
Pain	Grade 3	5 (<0.1)	2 (<0.1)
Pyrexia	Grade 3	37 (0.3)	7 (0.1)

	Grade 4	2 (<0.1)	0
Shoulder injury related to vaccine administration	Grade 3	0	1 (<0.1)
Stenosis	Grade 4	0	1 (<0.1)
Swelling	Grade 3	1 (<0.1)	0
Swelling face	Grade 3	1 (<0.1)	1 (<0.1)
Systemic inflammatory response syndrome	Grade 4	1 (<0.1)	0
Ulcer	Grade 3	0	1 (<0.1)
Vaccination site bruising	Grade 3	1 (<0.1)	0
Vaccination site erythema	Grade 3	3 (<0.1)	8 (0.1)
	Grade 4	1 (<0.1)	0
Vaccination site hypoaesthesia	Grade 3	0	1 (<0.1)
Vaccination site induration	Grade 3	1 (<0.1)	0
	Grade 4	1 (<0.1)	0
Vaccination site oedema	Grade 3	0	4 (<0.1)
	Grade 4	1 (<0.1)	0
Vaccination site pain	Grade 3	15 (0.1)	12 (0.1)
	Grade 4	6 (<0.1)	2 (<0.1)
Vaccination site pruritus	Grade 3	0	1 (<0.1)
Vaccination site rash	Grade 3	0	1 (<0.1)
Vaccination site reaction	Grade 3	1 (<0.1)	0
Vaccination site swelling	Grade 3	0	2 (<0.1)
**Investigations**			
Amylase increased	Grade 3	1 (<0.1)	0
Blood potassium abnormal	Grade 3	2 (<0.1)	0
Blood potassium decreased	Grade 3	2 (<0.1)	0
Blood pressure increased	Grade 3	1 (<0.1)	0
Blood urine present	Grade 3	1 (<0.1)	0
Body temperature abnormal	Grade 3	0	1 (<0.1)
Body temperature increased	Grade 3	2 (<0.1)	1 (<0.1)
C-reactive protein abnormal	Grade 3	1 (<0.1)	0
C-reactive protein increased	Grade 3	2 (<0.1)	1 (<0.1)
	Grade 4	1 (<0.1)	0
Electrocardiogram P wave abnormal	Grade 3	1 (<0.1)	0
Electrocardiogram normal	Grade 3	1 (<0.1)	0
Lipase abnormal	Grade 3	1 (<0.1)	0
Liver function test abnormal	Grade 4	1 (<0.1)	0
Lymphocyte count decreased	Grade 3	1 (<0.1)	0
Oxygen saturation decreased	Grade 3	0	1 (<0.1)
	Grade 4	1 (<0.1)	0
**Injury, poisoning and procedural complications**			
Ankle fracture	Grade 3	0	1 (<0.1)
Back injury	Grade 3	1 (<0.1)	0
Contusion	Grade 3	1 (<0.1)	0
Craniocerebral injury	Grade 4	1 (<0.1)	1 (<0.1)
Eye injury	Grade 3	0	1 (<0.1)
Fall	Grade 3	0	4 (<0.1)

	Grade 4	2 (<0.1)	0
Foot fracture	Grade 3	0	3 (<0.1)
Hand fracture	Grade 3	1 (<0.1)	1 (<0.1)
Injury	Grade 3	1 (<0.1)	1 (<0.1)
	Grade 5	0	1 (<0.1)
Injury corneal	Grade 3	0	1 (<0.1)
Joint dislocation	Grade 3	3 (<0.1)	0
Joint injury	Grade 3	1 (<0.1)	0
Ligament sprain	Grade 4	0	1 (<0.1)
Limb injury	Grade 3	0	1 (<0.1)
Lower limb fracture	Grade 3	0	1 (<0.1)
Meniscus injury	Grade 4	1 (<0.1)	0
Muscle strain	Grade 3	1 (<0.1)	0
Post procedural hypotension	Grade 3	0	1 (<0.1)
Radius fracture	Grade 3	0	1 (<0.1)
	Grade 4	0	1 (<0.1)
Repetitive strain injury	Grade 3	1 (<0.1)	0
Road traffic accident	Grade 3	1 (<0.1)	1 (<0.1)
Skin laceration	Grade 4	1 (<0.1)	0
Tendon injury	Grade 4	1 (<0.1)	0
Tendon rupture	Grade 4	1 (<0.1)	0
Thermal burn	Grade 3	0	1 (<0.1)
Upper limb fracture	Grade 3	0	2 (<0.1)
Wrist fracture	Grade 3	1 (<0.1)	0
**Social circumstances**			
Homicide	Grade 5	0	1 (<0.1)

Oxford Trial Group

Marites Aban	NIHR Imperial Clinical Research Facility, London, UK
Kushala W M Abeysekera	University Hospitals Bristol & Weston NHS Foundation Trust
Jeremy Aboagye	Jenner Institute, Nuffield Department of Medicine, University of Oxford, UK
Matthew Adam	Clinical Infection Research Group, Regional Infectious Diseases Unit, NHS Lothian, UK
Kirsty Adams	NIHR UCLH Clinical Research Facility, London, UK
James P. Adamson	Public Health Wales NHS Trust, Cardiff, UK
Gbadebo Adewetan	London Northwest University Healthcare, Northwick Park Hospital, London, UK
Syed Adlou	Oxford Vaccine Group, Department of Paediatrics, University of Oxford, UK
Khatija Ahmed	Setshababa Research Centre, Tshwane, South Africa
Yasmeen Akhalwaya	Family Centre for Research with Ubuntu, Department of Paediatrics, University of Stellenbosch, Cape Town, South Africa
Saajida Akhalwaya	Wits Reproductive Health and HIV Institute, Faculty of Health Sciences, University of the Witwatersrand, Johannesburg, South Africa
Andrew Alcock	Oxford Biomedica PLC, Transport Way, Cowley, Oxford
Aabidah Ali	Jenner Institute, Nuffield Department of Medicine, University of Oxford, UK
Elizabeth R. Allen	Jenner Institute, Nuffield Department of Medicine, University of Oxford, UK
Lauren Allen	National Infection Service, Public Health England, UK
Felipe B. Alvernaz	Universidade Federal de São Paulo, Brazil
Fábio Santos Amorim	Instituto D’Or de Pesquisa e Ensino (IDOR), Salvador, Brazil and Hospital São Rafael and Hospital Couto Maia, Salvador, Brazil
Cláudia Sala Andrade	Hospital Universitário de Santa Maria, Santa Maria, Brazil
Foteini Andritsou	Department of Paediatrics, University of Oxford, UK
Rachel Anslow	Oxford Vaccine Group, Department of Paediatrics, University of Oxford, UK
Edward H. Arbe-Barnes	University of Oxford Medical School, Medical Sciences Division, University of Oxford, UK
Markus P. Ariaans	Department of Infection, Immunity and Cardiovascular Disease, University of Sheffield, UK
Beatriz Arns	Hospital de Clinicas de Porto Alegre, Universidade Federal do Rio Grande do Sul, Brazil
Laiana Arruda	Instituto D’Or de Pesquisa e Ensino (IDOR), Salvador, Brazil and Hospital São Rafael, Salvador, Brazil
Luiza Assad	Instituto D’Or de Pesquisa e Ensino (IDOR), Rio de Janeiro, Brazil and Hospital Quinta D'OR, Rede D’Or São Luiz, Rio de Janeiro, Brazil
Paula De Almeida Azi	Instituto D’Or de Pesquisa e Ensino (IDOR), Salvador, Brazil and Hospital São Rafael, Salvador, Brazil / Hospital Aliança/ Rede D’OR, Brazil
Lorena De Almeida Azi	Instituto D’Or de Pesquisa e Ensino (IDOR), Salvador, Brazil and Hospital São Rafael, Salvador, Brazil / Hospital das Clínicas (Unidade de atenção psicossocial do HUPES)

Gavin Babbage	NIHR Southampton Clinical Research Facility, Southampton, UK
Catherine Bailey	Aneurin Bevan University Health Board, Newport, Wales, UK
Kenneth F. Baker	Department of Infection and Tropical Medicine, Newcastle upon Tyne Hospitals NHS Foundation Trust and Translational and Clinical Research Institute, Immunity and Inflammation Theme, Newcastle University
Megan Baker	Jenner Institute, Nuffield Department of Medicine, University of Oxford, UK
Natalie Baker	National Infection Service, Public Health England, UK
Philip Baker	University of Oxford Medical School, Medical Sciences Division, University of Oxford, UK
Ioana Baleanu	Clinical BioManufacturing Facility, Jenner Institute, University of Oxford, UK
Danieli Bandeira	Postgraduate Programme in Nursing, Universidade Federal de Santa Maria, Santa Maria, Brazil
Anna Bara	NIHR Imperial Clinical Research Facility, London, UK
Marcella A. S. Barbosa	Centro de Estudos e Pesquisas em Moléstias Infecciosas, Rio Grande do Norte, Brazil
Deborah Barker	Oxford University Hospitals NHS Trust, Oxford, UK
Gavin D Barlow	Experimental Medicine & Biomedicine, Hull York Medical School, UK
Eleanor Barnes	Nuffield Department of Medicine, University of Oxford, UK
Andrew S. Barr	Department of Infection and Tropical Medicine, Newcastle upon Tyne Hospitals NHS Foundation Trust, UK
Jordan R. Barrett	Jenner Institute, Nuffield Department of Medicine, University of Oxford, UK
Jessica Barrett	London Northwest University Healthcare, Northwick Park Hospital, London, UK
Kelly Barrett	Nuffield Department of Women's and Reproductive Health, University of Oxford, UK
Louise Bates	Oxford Vaccine Group, Department of Paediatrics, University of Oxford, UK
Alexander Batten	Clinical BioManufacturing Facility, Jenner Institute, University of Oxford, UK
Kirsten Beadon	Oxford Vaccine Group, Department of Paediatrics, University of Oxford, UK
Emily Beales	Vaccine Institute, Institute of Infection & Immunity, St. Georges, University of London and St Georges University Hospitals NHS Trust, London, UK
Rebecca Beckley	Oxford Vaccine Group, Department of Paediatrics, University of Oxford, UK
Sandra Belij-Rammerstorfer	Jenner Institute, Nuffield Department of Medicine, University of Oxford, UK
Jonathan Bell	Oxford Vaccine Group, Department of Paediatrics, University of Oxford, UK
Duncan Bellamy	Jenner Institute, Nuffield Department of Medicine, University of Oxford, UK
Sue Belton	The University of Nottingham Health Service, Cripps Health Centre, University Park, Nottingham, UK

Adam Berg	Jenner Institute, Nuffield Department of Medicine, University of Oxford, UK
Laura Bermejo	Nuffield Department of Clinical Medicine, University of Oxford, UK
Eleanor Berrie	Clinical BioManufacturing Facility, Jenner Institute, University of Oxford, UK
Lisa Berry	NIHR Southampton Clinical Research Facility, Southampton, UK
Daniella Berzsenyi	Oxford Vaccine Group, Department of Paediatrics, University of Oxford, UK
Amy Beveridge	Oxford Vaccine Group, Department of Paediatrics, University of Oxford, UK
Kevin R Bewley	National Infection Service, Public Health England, UK
Inderjeet Bharaj	London Northwest University Healthcare, Northwick Park Hospital, London, UK
Sutika Bhikha	VIDA - Vaccines and Infectious Diseases Analytical Research Unit, Diepkloof, Soweto, South Africa
Asad E. Bhorat	Soweto Clinical Trials Centre, Johannesburg, South Africa
Zahedah E. Bhorat	Soweto Clinical Trials Centre, Johannesburg, South Africa
Else Margreet Bijker	Oxford Vaccine Group, Department of Paediatrics, University of Oxford, UK
Sarah Birch	Academic Directorate of Communicable Diseases and Specialised Medicine, Sheffield Teaching Hospitals NHS Foundation Trust
Gurpreet Birch	Cobra Biologics, Keele Science Park, UK
Kathryn Birchall	Clinical Research Facility, Sheffield Teaching Hospitals NHS Foundation Trust, UK
Adam Bird	Oxford Biomedica PLC, Transport Way, Cowley, Oxford
Olivia Bird	Vaccine Institute, Institute of Infection & Immunity, St. Georges, University of London and St Georges University Hospitals NHS Trust, London, UK
Karen Bisnauthsing	NIHR BRC at Guy's and St Thomas' NHS Foundation Trust, UK
Mustapha Bittaye	Jenner Institute, Nuffield Department of Medicine, University of Oxford, UK
Luke Blackwell	Oxford Vaccine Group, Department of Paediatrics, University of Oxford, UK
Rachel Blacow	Clinical Research Facility, Queen Elizabeth University Hospital, Glasgow, UK
Heather Bletchly	Oxford Vaccine Group, Department of Paediatrics, University of Oxford, UK
Caitlin L Blundell	Department of Biochemistry, University of Oxford, UK
Susannah R Blundell	Department of Biochemistry, University of Oxford, UK
Pritesh Bodalia	Pharmacy, University College London Hospitals NHS Trust, UK
Emma Bolam	Clinical BioManufacturing Facility, Jenner Institute, University of Oxford, UK
Elena Boland	Clinical BioManufacturing Facility, Jenner Institute, University of Oxford, UK
Daan Bormans	Halix B.V., Tinbergenweg 1, 2333 BB Leiden, Netherlands
Nicola Borthwick	Jenner Institute, Nuffield Department of Medicine, University of Oxford, UK
Francesca Bowring	Oxford University Hospitals NHS Foundation Trust, Oxford, UK

Amy Boyd	Jenner Institute, Nuffield Department of Medicine, University of Oxford, UK
Penny Bradley	Department of Pharmacy, Newcastle upon Tyne Hospitals NHS Foundation Trust, UK
Tanja Brenner	Clinical BioManufacturing Facility, Jenner Institute, University of Oxford, UK
Alice Bridges-Webb	Oxford Vaccine Group, Department of Paediatrics, University of Oxford, UK
Phillip Brown	National Infection Service, Public Health England, UK
Claire Brown	NIHR/Wellcome Trust Birmingham Clinical Research Facility, Birmingham, UK
Charlie Brown-O'Sullivan	Jenner Institute, Nuffield Department of Medicine, University of Oxford, UK
Scott Bruce	Cobra Biologics, Keele Science Park, UK
Emily Brunt	National Infection Service, Public Health England, UK
William Budd	NIHR Imperial Clinical Research Facility, London, UK
Yusuf A. Bulbulia	Soweto Clinical Trials Centre, Johannesburg, South Africa
Melanie Bull	Oxford Biomedica PLC, Transport Way, Cowley, Oxford
Jamie Burbage	Oxford Vaccine Group, Department of Paediatrics, University of Oxford, UK
Aileen Burn	Research Directorate, Newcastle upon Tyne Hospitals NHS Foundation Trust, UK
Karen R Buttigieg	National Infection Service, Public Health England, UK
Nicholas Byard	Jenner Institute, Nuffield Department of Medicine, University of Oxford, UK
Ingrid Cabrera Puig	Jenner Institute, Nuffield Department of Medicine, University of Oxford, UK
Anna Calvert	Vaccine Institute, Institute of Infection & Immunity, St. Georges, University of London and St Georges University Hospitals NHS Trust, London, UK
Susana Camara	Oxford Vaccine Group, Department of Paediatrics, University of Oxford, UK
Michelangelo Cao	Nuffield Department of Clinical Neurosciences, University of Oxford, UK
Federica Cappuccini	Jenner Institute, Nuffield Department of Medicine, University of Oxford, UK
Rita Cardona	Universidade Federal de São Paulo, Brazil
João R. Cardoso	Instituto D’Or de Pesquisa e Ensino (IDOR), Rio de Janeiro, Brazil and Hospital Quinta D'OR, Rede D’Or São Luiz, Rio de Janeiro, Brazil
Melanie Carr	Oxford Vaccine Group, Department of Paediatrics, University of Oxford, UK
Miles W Carroll	National Infection Service, Public Health England, UK
Andrew Carson-Stevens	Division of Population Medicine, School of Medicine, Cardiff University, UK
Yasmin de M. Carvalho	Centro de Estudos e Pesquisas em Moléstias Infecciosas, Rio Grande do Norte, Brazil
Helen R Casey	North Bristol NHS Trust, Bristol, UK
Paul Cashen	Pall Europe Ltd, Harbourgate Business Park, Portsmouth, UK

Thais R. Y Castro	Postgraduate Programme in Pharmaceutical Sciences, Universidade Federal de Santa Maria, Santa Maria, Brazil
Lucia Carratala Castro	Vaccine Institute, Institute of Infection & Immunity, St. Georges, University of London and St Georges University Hospitals NHS Trust, London, UK
Katrina Cathie	University Hospital Southampton NHS Foundation Trust, UK
Ana Cavey	Nuffield Department of Clinical Neurosciences, University of Oxford, UK
José Cerbino-Neto	Instituto D'Or de Pesquisa e Ensino (IDOR), Rio de Janeiro, Brazil
Luiz Fernando F. Cezar	Instituto D'Or de Pesquisa e Ensino (IDOR), Rio de Janeiro, Brazil
Jim Chadwick	National Infection Service, Public Health England, UK
David Chapman	Oxfordshire Clinical Commissioning Group, Oxford, UK
Sue Charlton	National Infection Service, Public Health England, UK
Katerina S. Cheliotis	Department of Clinical Sciences, Liverpool School of Tropical Medicine, Liverpool, UK
Irina Chelysheva	Oxford Vaccine Group, Department of Paediatrics, University of Oxford, UK
Oliver Chester	Oxford Vaccine Group, Department of Paediatrics, University of Oxford, UK
Sunder Chita	London Northwest University Healthcare, Northwick Park Hospital, London, UK
Emily Chiplin	National Infection Service, Public Health England, UK
Jee-Sun Cho	Jenner Institute, Nuffield Department of Medicine, University of Oxford, UK
Liliana Cifuentes	Kennedy Institute of Rheumatology, Nuffield Department of Orthopaedics, The University of Oxford, UK
Elizabeth Clark	Oxford Vaccine Group, Department of Paediatrics, University of Oxford, UK
Matthew Clark	Oxford Vaccine Group, Department of Paediatrics, University of Oxford, UK
Rachel Colin-Jones	Oxford Vaccine Group, Department of Paediatrics, University of Oxford, UK
Sarah L. K. Collins	Nuffield Department of Women's and Reproductive Health, University of Oxford, UK
Hayley Colton	Department of Infection and Tropical Medicine, Sheffield Teaching Hospitals NHS Foundation Trust
Christopher P. Conlon	Nuffield Department of Medicine, University of Oxford, UK
Sean Connarty	London Northwest University Healthcare, Northwick Park Hospital, London, UK
Naomi S. Coombes	National Infection Service, Public Health England, UK
Cushla Cooper	Nuffield Dept of Orthopaedics Rheumatology and Musculoskeletal Medicine, University of Oxford, UK
Rachel Cooper	Oxford Vaccine Group, Department of Paediatrics, University of Oxford, UK
Lynne Cornelissen	Family Centre for Research with Ubuntu, Department of Paediatrics, University of Stellenbosch, Cape Town, South Africa
Tumena Corrah	London Northwest University Healthcare, Northwick Park Hospital, London, UK

Catherine A. Cosgrove	Vaccine Institute, Institute of Infection & Immunity, St. Georges, University of London and St Georges University Hospitals NHS Trust, London, UK
Fernanda Barroso Costa	Instituto D'Or de Pesquisa e Ensino (IDOR), Rio de Janeiro, Brazil and Hospital Quinta D'OR, Rede D'Or São Luiz, Rio de Janeiro, Brazil
Tony Cox OBE	NIHR National Biosample Centre, Milton Keynes, UK
Wendy E. M. Crocker	Jenner Institute, Nuffield Department of Medicine, University of Oxford, UK
Sarah Crosbie	Oxford University Hospitals NHS Foundation Trust, Oxford, UK
Dan Cullen	Nuffield Department of Clinical Neurosciences, University of Oxford, UK
Debora R. M. F. Cunha	Hospital de Clinicas de Porto Alegre, Universidade Federal do Rio Grande do Sul, Brazil
Christina J Cunningham	Oxford Vaccine Group, Department of Paediatrics, University of Oxford, UK
Fiona C. Cuthbertson	Nuffield Department of Clinical Neurosciences, University of Oxford, UK
Daniel Marinho da Costa	Instituto D'Or de Pesquisa e Ensino (IDOR), Rio de Janeiro, Brazil
Suzete N. Farias Da Guarda	Universidade Federal da Bahia / Instituto D'Or de Pesquisa e Ensino (IDOR), Salvador, Brazil and Hospital São Rafael, Salvador, Brazil
Larissa P. da Silva	Hospital de Clinicas de Porto Alegre, Universidade Federal do Rio Grande do Sul, Brazil
Antonio Carlos Da Silva Moraes	Instituto D'Or de Pesquisa e Ensino (IDOR), Rio de Janeiro, Brazil
Brad E. Damratoski	Clinical BioManufacturing Facility, Jenner Institute, University of Oxford, UK
Zsofia Danos	Vaccine Institute, Institute of Infection & Immunity, St. Georges, University of London and St Georges University Hospitals NHS Trust, London, UK
Maria T. D. C. Dantas	Centro de Estudos e Pesquisas em Moléstias Infecciosas, Rio Grande do Norte, Brazil
Mehreen S Datoo	Jenner Institute, Nuffield Department of Medicine, University of Oxford, UK
Chandrabali Datta	Clinical BioManufacturing Facility, Jenner Institute, University of Oxford, UK
Malika Davids	Centre for Lung Infection and Immunity, Division of Pulmonology, Department of Medicine and UCT Lung Institute & South African MRC/UCT Centre for the Study of Antimicrobial Resistance, University of Cape Town, Cape Town, South Africa
Sarah L Davies	Cobra Biologics, Keele Science Park, UK
Kelly Davies	Department of Clinical Sciences, Liverpool School of Tropical Medicine, Liverpool, UK
Hannah Davies	Jenner Institute, Nuffield Department of Medicine, University of Oxford, UK
Sophie Davies	Jenner Institute, Nuffield Department of Medicine, University of Oxford, UK
Judith Davies	Oxford Vaccine Group, Department of Paediatrics, University of Oxford, UK
Elizabeth J. Davis	Oxford University Hospitals NHS Foundation Trust, Oxford, UK

John Davis	Research Directorate, Newcastle upon Tyne Hospitals NHS Foundation Trust, UK
José A. M. de Carvalho	Department of Clinical and Toxicological Analysis- Universidade Federal de Santa Maria, Santa Maria, Brazil
Jeanne De Jager	Family Centre for Research with Ubuntu, Department of Paediatrics, University of Stellenbosch, Cape Town, South Africa
Sergio Carlos de A. de Jesus Jr.	Instituto D'Or de Pesquisa e Ensino (IDOR), Rio de Janeiro, Brazil
Lis Moreno De Oliveira Kalid	Instituto D'Or de Pesquisa e Ensino (IDOR), Salvador, Brazil and Rede D'OR São Luiz, Brazil
David Dearlove	Department of Physiology Anatomy and Genetics, University of Oxford, UK
Tesfaye Demissie	Oxford Vaccine Group, Department of Paediatrics, University of Oxford, UK
Amisha Desai	Pharmacy Department, University Hospitals Birmingham NHS Foundation Trust
Stefania Di Marco	Advent SRL, Italy
Claudio Di Maso	Oxford Vaccine Group, Department of Paediatrics, University of Oxford, UK
Tanya Dinesh	Oxford Vaccine Group, Department of Paediatrics, University of Oxford, UK
Claire Docksey	Cobra Biologics, Keele Science Park, UK
Tao Dong	Chinese Academy of Medical Sciences Oxford Institute, Nuffield Department of Medicine, Oxford University, UK
Francesca R. Donnellan	Jenner Institute, Nuffield Department of Medicine, University of Oxford, UK
Tannyth Gomes Dos Santos	Centro de Estudos e Pesquisas em Moléstias Infecciosas, Rio Grande do Norte, Brazil
Thainá G. dos Santos	Hospital de Clinicas de Porto Alegre, Universidade Federal do Rio Grande do Sul, Brazil
Érika Eberlline Pacheco dos Santos	Postgraduate Programme in Nursing, Universidade Federal de Santa Maria, Santa Maria, Brazil
Naomi Douglas	Oxford Vaccine Group, Department of Paediatrics, University of Oxford, UK
Charlotte Downing	University of Oxford Medical School, Medical Sciences Division, University of Oxford, UK
Jonathan Drake	University of Oxford Medical School, Medical Sciences Division, University of Oxford, UK
Rachael Drake-Brockman	Oxford Vaccine Group, Department of Paediatrics, University of Oxford, UK
Ruth Elizabeth Drury	Oxford Vaccine Group, Department of Paediatrics, University of Oxford, UK
Joan Du Plessis	Family Centre for Research with Ubuntu, Department of Paediatrics, University of Stellenbosch, Cape Town, South Africa
Susanna J. Dunachie	Centre for Tropical Medicine & Global Health, Nuffield Department of Medicine, Oxford, UK

Andrew D. S. Duncan	Clinical Infection Research Group, Regional Infectious Diseases Unit, NHS Lothian, UK
Nicholas J. W. Easom	Hull University Teaching Hospitals NHS Trust, Hull, UK
Mandy Edwards	Aneurin Bevan University Health Board, Newport, Wales, UK
Nick J. Edwards	Jenner Institute, Nuffield Department of Medicine, University of Oxford, UK
Frances Edwards	University Hospitals Bristol & Weston NHS Foundation Trust
Omar M. El Muhanna	Clinical BioManufacturing Facility, Jenner Institute, University of Oxford, UK
Sean C. Elias	Jenner Institute, Nuffield Department of Medicine, University of Oxford, UK
Branwen Ellison-Handley	Clinical Research Facility, Sheffield Teaching Hospitals NHS Foundation Trust, UK
Michael J. Elmore	National Infection Service, Public Health England, UK
Marcus Rex English	University of Oxford Medical School, Medical Sciences Division, University of Oxford, UK
Aliasgar Esmail	Centre for Lung Infection and Immunity, Division of Pulmonology, Department of Medicine and University of Cape Town Lung Institute, South Africa
Yakub Moosa Essack	Soweto Clinical Trials Centre, Johannesburg, South Africa
Mutjaba Ghulam Farooq	Oxford Vaccine Group, Department of Paediatrics, University of Oxford, UK
Sofiya Fedosyuk	Jenner Institute, Nuffield Department of Medicine, University of Oxford, UK
Sally Felle	Oxford Vaccine Group, Department of Paediatrics, University of Oxford, UK
Susie Ferguson	Clinical Infection Research Group, Regional Infectious Diseases Unit, NHS Lothian, UK
Carla Ferreira Da Silva	Oxford Vaccine Group, Department of Paediatrics, University of Oxford, UK
Samantha Field	Nuffield Department of Population Health, University of Oxford, UK
Richard Fisher	Clinical BioManufacturing Facility, Jenner Institute, University of Oxford, UK
James Fletcher	NIHR Imperial Clinical Research Facility, London, UK
Hazel Fofie	Vaccine Institute, Institute of Infection & Immunity, St. Georges, University of London and St Georges University Hospitals NHS Trust, London, UK
Henry Fok	NIHR BRC at Guy's and St Thomas' NHS Foundation Trust and King's College London British Heart Foundation Centre, School of Cardiovascular Medicine and Sciences, UK
Ross Fothergill	National Infection Service, Public Health England, UK
Karen J Ford	Oxford Vaccine Group, Department of Paediatrics, University of Oxford, UK
Jamie Fowler	Jenner Institute, Nuffield Department of Medicine, University of Oxford, UK
Pedro H. A. Fraiman	Centro de Estudos e Pesquisas em Moléstias Infecciosas, Rio Grande do Norte, Brazil

Emma Francis	Oxford Vaccine Group, Department of Paediatrics, University of Oxford, UK
Marília M. Franco	Instituto D'Or de Pesquisa e Ensino (IDOR), Salvador, Brazil and Rede D'OR São Luiz, Brazil
John Frater	Nuffield Department of Medicine, University of Oxford, UK
Marilúcia S. M. Freire	Centro de Estudos e Pesquisas em Moléstias Infecciosas, Rio Grande do Norte, Brazil
Samantha H Fry	Family Centre for Research with Ubuntu, Department of Paediatrics, University of Stellenbosch, Cape Town, South Africa
Sabrina Fudge	University Hospitals Bristol & Weston NHS Foundation Trust
Renato Furlan Filho	Universidade Federal de São Paulo, Brazil
Julie Furze	Jenner Institute, Nuffield Department of Medicine, University of Oxford, UK
Michelle Fuskova	Jenner Institute, Nuffield Department of Medicine, University of Oxford, UK
Pablo Galian-Rubio	Clinical BioManufacturing Facility, Jenner Institute, University of Oxford, UK
Harriet Garlant	National Infection Service, Public Health England, UK
Madita Gavrila	Oxford University Hospitals NHS Foundation Trust, Oxford, UK
Karyna A. Gibbons	Oxford University Hospitals NHS Foundation Trust, Oxford, UK
Ciaran Gilbride	Jenner Institute, Nuffield Department of Medicine, University of Oxford, UK
Hardeep Gill	Oxford University Hospitals NHS Foundation Trust, Oxford, UK
Kerry Godwin	National Infection Service, Public Health England, UK
Karishma Gokani	NIHR/Wellcome Trust Birmingham Clinical Research Facility, Birmingham, UK
Maria Luisa Freire Gonçalves	Instituto D'Or de Pesquisa e Ensino (IDOR), Salvador, Brazil and Hospital São Rafael, Salvador, Brazil and CEDAP - Centro Estadual Especializado em Diagnóstico, Assistência e Pesquisa, Brazil
Isabela G. S. Gonzalez	Universidade Federal de São Paulo, Brazil
Jack Goodall	London Northwest University Healthcare, Northwick Park Hospital, London, UK
Jayne Goodwin	Health and Care Research Wales, Cardiff, UK
Amina Goondiwala	Soweto Clinical Trials Centre, Johannesburg, South Africa
Katherine Gordon-Quayle	Nuffield Department of Surgical Sciences, University of Oxford, UK
Giacomo Gorini	Jenner Institute, Nuffield Department of Medicine, University of Oxford, UK
Alvaro Goyanna	Instituto D'Or de Pesquisa e Ensino (IDOR), Rio de Janeiro, Brazil and Hospital Quinta D'OR, Rede D'Or São Luiz, Rio de Janeiro, Brazil
Janet Grab	Wits Reproductive Health and HIV Institute, Faculty of Health Sciences, University of the Witwatersrand, Johannesburg, South Africa
Lara Gracie	Oxford Vaccine Group, Department of Paediatrics, University of Oxford, UK
Justin Green	AstraZeneca BioPharmaceuticals PLC
Nicola Greenwood	Jenner Institute, Nuffield Department of Medicine, University of Oxford, UK
Johann Greffrath	VIDA - Vaccines and Infectious Diseases Analytical Research Unit, Diepkloof, Soweto, South Africa

Marisa M. Groenewald	Family Centre for Research with Ubuntu, Department of Paediatrics, University of Stellenbosch, Cape Town, South Africa
Anishka Gunawardene	London Northwest University Healthcare, Northwick Park Hospital, London, UK
Gaurav Gupta	Jenner Institute, Nuffield Department of Medicine, University of Oxford, UK
Mark Hackett	University Hospitals Bristol & Weston NHS Foundation Trust
Bassam Hallis	National Infection Service, Public Health England, UK
Mainga Hamaluba	KEMRI-Wellcome Trust Research Programme and Centre for Tropical Medicine & Global Health, Nuffield Department of Medicine, Oxford, UK
Elizabeth Hamilton	Nuffield Department of Population Health, University of Oxford, UK
Joseph Hamlyn	Oxford Vaccine Group, Department of Paediatrics, University of Oxford, UK
Daniel Hammersley	The University of Nottingham Health Service, Cripps Health Centre, University Park, Nottingham, UK
Aidan T. Hanrath	Department of Infection and Tropical Medicine, Newcastle upon Tyne Hospitals NHS Foundation Trust and Translational and Clinical Research Institute, Immunity and Inflammation Theme, Newcastle University
Brama Hanumunthadu	Oxford Vaccine Group, Department of Paediatrics, University of Oxford, UK
Stephanie A. Harris	Jenner Institute, Nuffield Department of Medicine, University of Oxford, UK
Clair Harris	NIHR BRC at Guy's and St Thomas' NHS Foundation Trust, UK
Thomas D. Harrison	Department of Infection and Tropical Medicine, Sheffield Teaching Hospitals NHS Foundation Trust, UK
Daisy Harrison	Oxford Vaccine Group, Department of Paediatrics, University of Oxford, UK
Tara A. Harris-Wright	Oxford University Hospitals NHS Foundation Trust, Oxford, UK
Thomas C. Hart	Oxford Vaccine Group, Department of Paediatrics, University of Oxford, UK
Birgit Hartnell	Vaccines Manufacturing and Innovation Centre, Oxford Science Park, Oxford, UK
John Haughney	Clinical Research Facility, Queen Elizabeth University Hospital, Glasgow, UK
Sophia Hawkins	Oxford Vaccine Group, Department of Paediatrics, University of Oxford, UK
Laís Y. M. Hayano	Universidade Federal de São Paulo, Brazil
Ian Head	University Hospitals Bristol & Weston NHS Foundation Trust
Paul T. Heath	Vaccine Institute, Institute of Infection & Immunity, St. Georges, University of London and St Georges University Hospitals NHS Trust, London, UK
John Aaron Henry	University of Oxford Medical School, Medical Sciences Division, University of Oxford, UK
Macarena Hermosin Herrera	Oxford University Hospitals NHS Foundation Trust, Oxford, UK
David B Hettle	Infection Sciences, North Bristol NHS Trust, Bristol, UK
Cristhiane Higa	Universidade Federal de São Paulo, Brazil

Jennifer Hill	Oxford Vaccine Group, Department of Paediatrics, University of Oxford, UK
Gina Hodges	Clinical BioManufacturing Facility, Jenner Institute, University of Oxford, UK
Susanne H. Hodgson	Jenner Institute, Nuffield Department of Medicine, University of Oxford, UK
Elizea Horne	Wits Reproductive Health and HIV Institute, Faculty of Health Sciences, University of the Witwatersrand, Johannesburg, South Africa
Mimi M. Hou	Jenner Institute, Nuffield Department of Medicine, University of Oxford, UK
Catherine F. Houlihan	Virology Department, University College London Hospitals NHS Trust, UK and Department of Infection and Immunity, University College London, UK
Elizabeth Howe	Oxford Vaccine Group, Department of Paediatrics, University of Oxford, UK
Nicola Howell	Oxford Vaccine Group, Department of Paediatrics, University of Oxford, UK
Jonathan Humphreys	Vaccines Manufacturing and Innovation Centre, Oxford Science Park, Oxford, UK
Holly E. Humphries	National Infection Service, Public Health England, UK
Katrina Hurley	University Hospitals Bristol & Weston NHS Foundation Trust
Claire Huson	AstraZeneca BioPharmaceuticals PLC
Catherine Hyams	Academic Respiratory Unit, University of Bristol, Southmead Hospital, Bristol, UK
Angela Hyder-Wright	Department of Clinical Sciences, Liverpool School of Tropical Medicine and Liverpool University Hospitals NHS Foundation Trust, UK
Sabina Ikram	Vaccine Institute, Institute of Infection & Immunity, St. Georges, University of London and St Georges University Hospitals NHS Trust, London, UK
Alka Ishwarbhai	Jenner Institute, Nuffield Department of Medicine, University of Oxford, UK
Poppy Iveson	University of Oxford Medical School, Medical Sciences Division, University of Oxford, UK
Vidyashankara Iyer	AstraZeneca BioPharmaceuticals PLC
Frederic Jackson	Clinical BioManufacturing Facility, Jenner Institute, University of Oxford, UK
Susan Jackson	Jenner Institute, Nuffield Department of Medicine, University of Oxford, UK
Shameem Jaumdally	Centre for Lung Infection and Immunity, Division of Pulmonology, Department of Medicine and UCT Lung Institute & South African MRC/UCT Centre for the Study of Antimicrobial Resistance, University of Cape Town, Cape Town, South Africa
Helen Jeffers	Nuffield Department of Medicine, University of Oxford, UK
Natasha Jesudason	MRC-University of Glasgow Centre for Virus Research & Department of Infectious Diseases, Queen Elizabeth University Hospital, UK
Christopher Jones	Infection Sciences, North Bristol NHS Trust, Bristol, UK
Kathryn Jones	Jenner Institute, Nuffield Department of Medicine, University of Oxford, UK

Elizabeth Jones	Oxford Vaccine Group, Department of Paediatrics, University of Oxford, UK
Marianna Rocha Jorge	Instituto D'Or de Pesquisa e Ensino (IDOR), Salvador, Brazil and Rede D'OR São Luiz, Brazil
Amar Joshi	Pall Europe Ltd, Harbourgate Business Park, Portsmouth, UK
Eduardo A. M. S. Júnior	Centro de Estudos e Pesquisas em Moléstias Infecciosas, Rio Grande do Norte, Brazil
Reshma Kailath	Jenner Institute, Nuffield Department of Medicine, University of Oxford, UK
Faeeza Kana	Soweto Clinical Trials Centre, Johannesburg, South Africa
Arnab Kar	London Northwest University Healthcare, Northwick Park Hospital, London, UK
Konstantinos Karampatsas	Vaccine Institute, Institute of Infection & Immunity, St. Georges, University of London and St Georges University Hospitals NHS Trust, London, UK
Mwila Kasanyinga	Oxford Vaccine Group, Department of Paediatrics, University of Oxford, UK
Linda J Kay	Department of Infection, Immunity and Cardiovascular Disease, University of Sheffield
Jade Keen	Oxford Vaccine Group, Department of Paediatrics, University of Oxford, UK
Johanna Kellett Wright	Infection Sciences, North Bristol NHS Trust, Bristol, UK
Elizabeth J. Kelly	AstraZeneca BioPharmaceuticals PLC
Debbie Kelly	NIHR Clinical Research Network: Thames Valley & South Midlands, UK and Oxford University Hospitals NHS Trust, Oxford, UK
Dearbhla M Kelly	Nuffield Department of Clinical Neurosciences, University of Oxford, UK
Sarah Kelly	Oxford Vaccine Group, Department of Paediatrics, University of Oxford, UK
David Kerr	Oxford Vaccine Group, Department of Paediatrics, University of Oxford, UK
Liaquat Khan	Oxford Vaccine Group, Department of Paediatrics, University of Oxford, UK
Baktash Khozoee	Jenner Institute, Nuffield Department of Medicine, University of Oxford, UK
Ankush Khurana	London Northwest University Healthcare, Northwick Park Hospital, London, UK
Sarah Kidd	University Hospitals Bristol & Weston NHS Foundation Trust
Annabel Killen	University of Oxford Medical School, Medical Sciences Division, University of Oxford, UK
Jasmin Kinch	Oxford Vaccine Group, Department of Paediatrics, University of Oxford, UK
Patrick Kinch	Oxford Vaccine Group, Department of Paediatrics, University of Oxford, UK
Lloyd D. W. King	Jenner Institute, Nuffield Department of Medicine, University of Oxford, UK
Thomas B King	University of Oxford Medical School, Medical Sciences Division, University of Oxford, UK

Lucy Kingham	Jenner Institute, Nuffield Department of Medicine, University of Oxford, UK
Paul Klenerman	Peter Medawar Building for Pathogen Research, NDM Experimental Medicine, University of Oxford, UK
Diana M. Kluczna	Hull University Teaching Hospitals NHS Trust, Hull, UK
Francesca Knapper	University Hospitals Bristol & Weston NHS Foundation Trust
Chanice Knight	National Infection Service, Public Health England, UK
Julian C. Knight	Wellcome Trust Centre for Human Genetics, University of Oxford, UK
Daniel Knott	National Infection Service, Public Health England, UK
Stanislava Koleva	Oxford Vaccine Group, Department of Paediatrics, University of Oxford, UK
Pedro M. Lages	Universidade Federal de São Paulo, Brazil
Matilda Lang	London Northwest University Healthcare, Northwick Park Hospital, London, UK
Gail Lang	Nuffield Dept of Orthopaedics Rheumatology and Musculoskeletal Medicine, University of Oxford, UK
Colin W Larkworthy	Jenner Institute, Nuffield Department of Medicine, University of Oxford, UK
Jessica P J Larwood	University of Oxford Medical School, Medical Sciences Division, University of Oxford, UK
Rebecca Law	Oxford University Hospitals NHS Foundation Trust, Oxford, UK
Alison M Lawrie	Jenner Institute, Nuffield Department of Medicine, University of Oxford, UK
Erica M. Lazarus	Perinatal HIV Research Unit (PHRU), Faculty of Health Sciences, University of the Witwatersrand, Johannesburg, South Africa
Amanda Leach	AstraZeneca BioPharmaceuticals PLC
Emily A. Lees	Oxford Vaccine Group, Department of Paediatrics, University of Oxford, UK
Alice Lelliott	Oxford Vaccine Group, Department of Paediatrics, University of Oxford, UK
Nana-Marie Lemm	NIHR Imperial Clinical Research Facility, London, UK
Álvaro Edson Ramos Lessa	Instituto D'Or de Pesquisa e Ensino (IDOR), Salvador, Brazil and Hospital São Rafael, Salvador, Brazil
Stephanie Leung	National Infection Service, Public Health England, UK
Yuanyuan Li	Jenner Institute, Nuffield Department of Medicine, University of Oxford, UK
Amelia M. Lias	Jenner Institute, Nuffield Department of Medicine, University of Oxford, UK
Konstantinos Liatsikos	Department of Clinical Sciences, Liverpool School of Tropical Medicine and Liverpool University Hospitals NHS Foundation Trust, UK
Aline Linder	Oxford Vaccine Group, Department of Paediatrics, University of Oxford, UK
Samuel Lipworth	Jenner Institute, Nuffield Department of Medicine, University of Oxford, UK
Shuchang Liu	Clinical BioManufacturing Facility, Jenner Institute, University of Oxford, UK

Xinxue Liu	Oxford Vaccine Group, Department of Paediatrics, University of Oxford, UK
Adam Lloyd	Clinical Infection Research Group, Regional Infectious Diseases Unit, NHS Lothian, UK
Stephanie Lloyd	Oxford University Hospitals NHS Foundation Trust, Oxford, UK
Lisa Loew	Clinical BioManufacturing Facility, Jenner Institute, University of Oxford, UK
Raquel Lopez Ramon	Jenner Institute, Nuffield Department of Medicine, University of Oxford, UK
Leandro Bonecker Lora	Instituto D'Or de Pesquisa e Ensino (IDOR), Rio de Janeiro, Brazil and Hospital Quinta D'OR, Rede D'OR, São Luiz, Rio de Janeiro, Brazil
Kleber Giovanni Luz	Universidade Federal do Rio Grande do Norte - UFRN, Brazil
Jonathan C. MacDonald	Department of Gastroenterology, Queen Elizabeth University Hospital, Glasgow, UK
Gordon MacGregor	Department of Respiratory Medicine, Queen Elizabeth University Hospital, Glasgow, UK
Meera Madhavan	Jenner Institute, Nuffield Department of Medicine, University of Oxford, UK
David O. Mainwaring	Pall Europe Ltd, Harbourgate Business Park, Portsmouth, UK
Edson Makambwa	Centre for Lung Infection and Immunity, Division of Pulmonology, Department of Medicine and UCT Lung Institute & South African MRC/UCT Centre for the Study of Antimicrobial Resistance, University of Cape Town, Cape Town, South Africa
Rebecca Makinson	Jenner Institute, Nuffield Department of Medicine, University of Oxford, UK
Mookho Malahleha	Setshababa Research Centre , Tshwane, South Africa
Ross Malamatsho	Setshababa Research Centre , Tshwane, South Africa
Garry Mallett	University of Oxford Medical School, Medical Sciences Division, University of Oxford, UK
Nicola Manning	University Hospitals Bristol & Weston NHS Foundation Trust
Kushal Mansatta	University of Oxford Medical School, Medical Sciences Division, University of Oxford, UK
Takalani Maoko	Perinatal HIV Research Unit, Faculty of Health Sciences, University of the Witwatersrand, Johannesburg, South Africa
Spyridoula Marinou	Oxford Vaccine Group, Department of Paediatrics, University of Oxford, UK
Emma Marlow	Jenner Institute, Nuffield Department of Medicine, University of Oxford, UK
Gabriela N. Marques	Instituto D'Or de Pesquisa e Ensino (IDOR), Salvador, Brazil and Rede D'OR São Luiz, Brazil
Paula Marriott	Kennedy Institute of Rheumatology, Nuffield Department of Orthopaedics, The University of Oxford, UK
Richard P. Marshall	AstraZeneca BioPharmaceuticals PLC
Julia L. Marshall	Jenner Institute, Nuffield Department of Medicine, University of Oxford, UK
Masebole Masenya	Wits Reproductive Health and HIV Institute, Faculty of Health Sciences, University of the Witwatersrand, Johannesburg, South Africa
Mduduzi Masilela	Setshababa Research Centre , Tshwane, South Africa

Shauna K. Masters	Nuffield Dept of Orthopaedics Rheumatology and Musculoskeletal Medicine, University of Oxford, UK
Moncy Mathew	Pharmacy Clinical Trials (Adult), Guy’s and St Thomas NHS Foundation Trust, UK
Hosea Matlebjane	Setshababa Research Centre , Tshwane, South Africa
Kedidimetse Matshidiso	Perinatal HIV Research Unit, Faculty of Health Sciences, University of the Witwatersrand, Johannesburg, South Africa
Olga Mazur	Oxford Vaccine Group, Department of Paediatrics, University of Oxford, UK
Andrea Mazzella	NIHR BRC at Guy's and St Thomas' NHS Foundation Trust, UK
Hugh McCaughan	Clinical Infection Research Group, Regional Infectious Diseases Unit, NHS Lothian, UK
Joanne McEwan	Oxford Vaccine Group, Department of Paediatrics, University of Oxford, UK
Joanna McGlashan	National Infection Service, Public Health England, UK
Lorna McInroy	National Infection Service, Public Health England, UK
Nicky McRobert	Oxford University Hospitals NHS Foundation Trust, Oxford, UK
Steve McSwiggan	Clinical Infection Research Group, Regional Infectious Diseases Unit, NHS Lothian, UK
Clare Megson	Oxford University Hospitals NHS Foundation Trust, Oxford, UK
Savviz Mehdipour	NIHR Imperial Clinical Research Facility, London, UK
Wilma Meijs	Halix B.V., Tinbergenweg 1, 2333 BB Leiden, Netherlands
Renata N. Á. Mendonça	Instituto D’Or de Pesquisa e Ensino (IDOR), Salvador, Brazil and Rede D’OR São Luiz, Brazil
Alexander J Mentzer	Wellcome Centre for Human Genetics, Nuffield Department of Medicine, University of Oxford, UK
Patricia Miralhes	Clinical Microbiology and Virology Department, University College London Hospitals NHS Trust, UK
Neginsadat Mirtorabi	University of Oxford Medical School, Medical Sciences Division, University of Oxford, UK
Celia Mitton	Oxford Vaccine Group, Department of Paediatrics, University of Oxford, UK
Sibusiso Mnyakeni	Perinatal HIV Research Unit, Faculty of Health Sciences, University of the Witwatersrand, Johannesburg, South Africa
Fiona Moghaddas	Department of Clinical Immunology, North Bristol NHS Trust, Bristol, UK
Kgaogelo Molapo	Setshababa Research Centre , Tshwane, South Africa
Mapule Moloi	Wits Reproductive Health and HIV Institute, Faculty of Health Sciences, University of the Witwatersrand, Johannesburg, South Africa
Maria Moore	Oxford Vaccine Group, Department of Paediatrics, University of Oxford, UK
Marni Moran	Oxford University Hospitals NHS Foundation Trust, Oxford, UK
Ella Morey	Oxford Vaccine Group, Department of Paediatrics, University of Oxford, UK
Róisín Morgans	Clinical BioManufacturing Facility, Jenner Institute, University of Oxford, UK
Susan J. Morris	Clinical BioManufacturing Facility, Jenner Institute, University of Oxford, UK

Sheila Morris	Clinical Infection Research Group, Regional Infectious Diseases Unit, NHS Lothian, UK
Hazel Morrison	Jenner Institute, Nuffield Department of Medicine, University of Oxford, UK
Franca Morselli	NIHR BRC at Guy's and St Thomas' NHS Foundation Trust, UK
Gertraud Morshead	Oxford Vaccine Group, Department of Paediatrics, University of Oxford, UK
Richard Morter	Jenner Institute, Nuffield Department of Medicine, University of Oxford, UK
Lynelle S. Mottay	Centre for Lung Infection and Immunity, Division of Pulmonology, Department of Medicine and UCT Lung Institute & South African MRC/UCT Centre for the Study of Antimicrobial Resistance, University of Cape Town, Cape Town, South Africa
Andrew Moultrie	VIDA - Vaccines and Infectious Diseases Analytical Research Unit, Diepkloof, Soweto, South Africa
Nathifa A. Moyo	Jenner Institute, Nuffield Department of Medicine, University of Oxford, UK
Mushiya Mpelembue	London Northwest University Healthcare, Northwick Park Hospital, London, UK
Sibekezelo Msomi	Perinatal HIV Research Unit, Faculty of Health Sciences, University of the Witwatersrand, Johannesburg, South Africa
Yvonne N. Mugodi	Perinatal HIV Research Unit, Faculty of Health Sciences, University of the Witwatersrand, Johannesburg, South Africa
Ekta Mukhopadhyay	Jenner Institute, Nuffield Department of Medicine, University of Oxford, UK
Jilly Muller	Oxford Vaccine Group, Department of Paediatrics, University of Oxford, UK
Alasdair P.S. Munro	NIHR Southampton Clinical Research Facility, Southampton, UK
Sarah Murphy	Oxford Vaccine Group, Department of Paediatrics, University of Oxford, UK
Philomena Mweu	Oxford Vaccine Group, Department of Paediatrics, University of Oxford, UK
Christopher Myerscough	Department of Clinical Sciences, Liverpool School of Tropical Medicine, Liverpool, UK
Gurudutt Naik	Aneurin Bevan University Health Board, Newport, Wales, UK
Kush Naker	NIHR/Wellcome Trust Birmingham Clinical Research Facility, Birmingham, UK
Eleni Nastouli	Department of Infection, Immunity and Inflammation UCL Great Ormond Street Institute of Child Health, London, UK
Bongani Ndlovu	Soweto Clinical Trials Centre, Johannesburg, South Africa
Elissavet Nikolaou	Department of Clinical Sciences, Liverpool School of Tropical Medicine, Liverpool, UK
Cecilia Njenga	NIHR Imperial Clinical Research Facility, London, UK
Helena C. Noal	Postgraduate Programme in Nursing, Universidade Federal de Santa Maria, Santa Maria, Brazil
Andrés Noé	Jenner Institute, Nuffield Department of Medicine, University of Oxford, UK
Gabrielle Novaes	Instituto D'Or de Pesquisa e Ensino (IDOR), Rio de Janeiro, Brazil and Hospital Quinta D'OR, Rede D'Or São Luiz, Rio de Janeiro, Brazil

Fay L Nugent	Jenner Institute, Nuffield Department of Medicine, University of Oxford, UK
Géssika Lanzillo A. Nunes	Centro de Estudos e Pesquisas em Moléstias Infecciosas, Rio Grande do Norte, Brazil
Katie O'Brien	Oxford Vaccine Group, Department of Paediatrics, University of Oxford, UK
Daniel O'Connor	Oxford Vaccine Group, Department of Paediatrics, University of Oxford, UK
Suzette Oelofse	Centre for Lung Infection and Immunity, Division of Pulmonology, Department of Medicine and UCT Lung Institute, University of Cape Town, Cape Town, South Africa
Blanché Oguti	Oxford Vaccine Group, Department of Paediatrics, University of Oxford, UK
Victoria Olchawski	Clinical BioManufacturing Facility, Jenner Institute, University of Oxford, UK
Neil J Oldfield	School of Life Sciences, University of Nottingham, Nottingham, UK
Marianne G. Oliveira	Centro de Estudos e Pesquisas em Moléstias Infecciosas, Rio Grande do Norte, Brazil
Catarina Oliveira	Clinical BioManufacturing Facility, Jenner Institute, University of Oxford, UK
Isabelle Silva Queiroz Oliveira	Instituto D'Or de Pesquisa e Ensino (IDOR), Salvador, Brazil and Hospital São Rafael, Salvador, Brazil and UNIME, Brazil
Aylin Oommen-Jose	VIDA - Vaccines and Infectious Diseases Analytical Research Unit, Diepkloof, Soweto, South Africa
Angela Oosthuizen	Wits Reproductive Health and HIV Institute, Faculty of Health Sciences, University of the Witwatersrand, Johannesburg, South Africa
Paula O'Reilly	Hull University Teaching Hospitals NHS Trust, Hull, UK
Peter J. O'Reilly	Oxford Vaccine Group, Department of Paediatrics, University of Oxford, UK
Piper Osborne	Oxford Vaccine Group, Department of Paediatrics, University of Oxford, UK
David R. J. Owen	NIHR Imperial Clinical Research Facility, London, UK
Lydia Owen	Oxford University Hospitals NHS Foundation Trust, Oxford, UK
Daniel R. Owens	NIHR Southampton Clinical Research Facility, Southampton, UK
Nelly Owino	Oxford Vaccine Group, Department of Paediatrics, University of Oxford, UK
Mihaela Pacurar	NIHR Southampton Clinical Research Facility, Southampton, UK
Brenda V. B. Paiva	Instituto D'Or de Pesquisa e Ensino (IDOR), Rio de Janeiro, Brazil
Edna M. F. Palhares	Centro de Estudos e Pesquisas em Moléstias Infecciosas, Rio Grande do Norte, Brazil
Susan Palmer	Aneurin Bevan University Health Board, Newport, Wales, UK
Helena M. R. T. Parracho	Clinical BioManufacturing Facility, Jenner Institute, University of Oxford, UK
Karen Parsons	Oxford University Hospitals NHS Foundation Trust, Oxford, UK
Dipak Patel	Clinical Research and Innovation Office, Sheffield Teaching Hospitals NHS Foundation Trust, UK
Bhumika Patel	Oxford Respiratory Trials Unit, Nuffield Department of Medicine, University of Oxford, UK

Faeezah Patel	Wits Reproductive Health and HIV Institute, Faculty of Health Sciences, University of the Witwatersrand, Johannesburg, South Africa
Maia Patrick-Smith	University of Oxford Medical School, Medical Sciences Division, University of Oxford, UK
Ruth O. Payne	Department of Infection and Tropical Medicine, Sheffield Teaching Hospitals NHS Foundation Trust and the Department of Infection, Immunity and Cardiovascular Disease, University of Sheffield, UK
Yanchun Peng	MRC Weatherall Institute of Molecular Medicine, NDM Experimental Medicine, University of Oxford, UK
Elizabeth J. Penn	National Infection Service, Public Health England, UK
Anna Pennington	Aneurin Bevan University Health Board, Newport, Wales, UK
Marco Polo Peralta Alvarez	Jenner Institute, Nuffield Department of Medicine, University of Oxford, UK
Bruno Pereira Stuchi	Instituto D'Or de Pesquisa e Ensino (IDOR), Rio de Janeiro, Brazil
Ana Luiza Perez	Hospital de Clinicas de Porto Alegre, Universidade Federal do Rio Grande do Sul, Brazil
Tanaraj Perinpanathan	Hull University Teaching Hospitals NHS Trust, Hull, UK
James Perring	University of Oxford Medical School, Medical Sciences Division, University of Oxford, UK
Rubeshan Perumal	Centre for Lung Infection and Immunity, Division of Pulmonology, Department of Medicine and UCT Lung Institute & South African MRC/UCT Centre for the Study of Antimicrobial Resistance, University of Cape Town, Cape Town, South Africa
Sahir Yusuf Petkar	Soweto Clinical Trials Centre, Johannesburg, South Africa
Tricia Philip	Perinatal HIV Research Unit, Faculty of Health Sciences, University of the Witwatersrand, Johannesburg, South Africa
Jennifer Phillips	University Hospitals Bristol & Weston NHS Foundation Trust
Mary Kgomotso Phohu	Setshababa Research Centre , Tshwane, South Africa
Lorinda Pickup	NIHR Cambridge Clinical Research Facility, Cambridge, UK
Sonja Pieterse	Family Centre for Research with Ubuntu, Department of Paediatrics, University of Stellenbosch, Cape Town, South Africa
Jessica Morgana Pinheiro	Hospital de Clinicas de Porto Alegre, Universidade Federal do Rio Grande do Sul, Brazil
Jo Piper	NIHR Cambridge Clinical Research Facility, Cambridge, UK
Dimitra Pipini	Jenner Institute, Nuffield Department of Medicine, University of Oxford, UK
Mary Plank	AstraZeneca BioPharmaceuticals PLC
Sinéad Plant	Clinical Infection Research Group, Regional Infectious Diseases Unit, NHS Lothian, UK
Samuel Pollard	Oxford Vaccine Group, Department of Paediatrics, University of Oxford, UK
Jennifer Pooley	North Bristol NHS Trust, Bristol, UK
Anil Pooran	Centre for Lung Infection and Immunity, Division of Pulmonology, Department of Medicine and UCT Lung Institute & South African MRC/UCT Centre for the Study of Antimicrobial Resistance, University of Cape Town, Cape Town, South Africa
Ian Poulton	Jenner Institute, Nuffield Department of Medicine, University of Oxford, UK

Claire Powers	Jenner Institute, Nuffield Department of Medicine, University of Oxford, UK
Fernando B. Presa	Centro de Estudos e Pesquisas em Moléstias Infecciosas, Rio Grande do Norte, Brazil
David A. Price	Department of Infection and Tropical Medicine, Newcastle upon Tyne Hospitals NHS Foundation Trust, UK
Vivien Price	NIHR/Wellcome Trust Birmingham Clinical Research Facility, Birmingham, UK
Marcelo R. Primeira	Postgraduate Programme in Nursing, Universidade Federal de Santa Maria, Santa Maria, Brazil
Pamela C. Proud	National Infection Service, Public Health England, UK
Samuel Provstgaard-Morys	Oxford Vaccine Group, Department of Paediatrics, University of Oxford, UK
Sophie Pueschel	Oxford University Hospitals NHS Foundation Trust, Oxford, UK
David Pulido	Jenner Institute, Nuffield Department of Medicine, University of Oxford, UK
Sheena Quaid	London Northwest University Healthcare, Northwick Park Hospital, London, UK
Ria Rabara	Oxford University Hospitals NHS Foundation Trust, Oxford, UK
Kajal Radia	University of Oxford Medical School, Medical Sciences Division, University of Oxford, UK
Durga Rajapaksa	National Infection Service, Public Health England, UK
Thurkka Rajeswaran	NIHR BRC at Guy's and St Thomas' NHS Foundation Trust, UK
Leonardo M. Ramos	Instituto D'Or de Pesquisa e Ensino (IDOR), Rio de Janeiro, Brazil
Alberto San Francisco Ramos	Vaccine Institute, Institute of Infection & Immunity, St. Georges, University of London and St Georges University Hospitals NHS Trust, London, UK
Fernando Ramos Lopez	Jenner Institute, Nuffield Department of Medicine, University of Oxford, UK
Tommy Rampling	Clinical Microbiology and Virology Department, University College London Hospitals NHS Trust, UK
Jade Rand	NIHR Southampton Clinical Research Facility, Southampton, UK
Helen Ratcliffe	Oxford Vaccine Group, Department of Paediatrics, University of Oxford, UK
Thomas Rawlinson	Jenner Institute, Nuffield Department of Medicine, University of Oxford, UK
David Rea	Clinical Research Network, West of England, UK
Byron Rees	Pall Europe Ltd, Harbourgate Business Park, Portsmouth, UK
Mila Resuello-Dauti	NIHR UCLH Clinical Research Facility, London, UK
Emilia Reyes Pabon	Clinical BioManufacturing Facility, Jenner Institute, University of Oxford, UK
Sarah Rhead	Oxford Vaccine Group, Department of Paediatrics, University of Oxford, UK
Tawassal Riaz	Infection Sciences, North Bristol NHS Trust, Bristol, UK
Marivic Ricamara	NIHR UCLH Clinical Research Facility, London, UK
Alexander J Richards	Hull University Teaching Hospitals NHS Trust, Hull, UK
Alex Richter	NIHR/Wellcome Trust Birmingham Clinical Research Facility & Institute of Immunology and Immunotherapy, University of Birmingham, UK

Neil D. Ritchie	Department of Infectious Diseases, Queen Elizabeth University Hospital, Glasgow, UK
Alexander J. Robbins	NIHR Imperial Clinical Research Facility, London, UK
Hannah Roberts	Oxford Vaccine Group, Department of Paediatrics, University of Oxford, UK
Ryan E Robinson	Department of Clinical Sciences, Liverpool School of Tropical Medicine and Liverpool University Hospitals NHS Foundation Trust, UK
Sophie Roche	University of Oxford Medical School, Medical Sciences Division, University of Oxford, UK
Christine S. Rollier	Oxford Vaccine Group, Department of Paediatrics, University of Oxford, UK
Louisa Rose	Jenner Institute, Nuffield Department of Medicine, University of Oxford, UK
Amy L. Ross Russell	NIHR Southampton Clinical Research Facility, Southampton, UK
Lindie Rossouw	Family Centre for Research with Ubuntu, Department of Paediatrics, University of Stellenbosch, Cape Town, South Africa
Simon Royal	School of Medicine, Division of Primary Care, University of Nottingham, Nottingham, UK
Indra Rudiansyah	Jenner Institute, Nuffield Department of Medicine, University of Oxford, UK
Kim Ryalls	Pharmacy Department, Sheffield Teaching Hospitals NHS Foundation Trust
Charlotte E. Sabine	NIHR/Wellcome Trust Birmingham Clinical Research Facility, Birmingham, UK
Stephen Saich	NIHR Southampton Clinical Research Facility, Southampton, UK
Jessica C Sale	NIHR/Wellcome Trust Birmingham Clinical Research Facility, Birmingham, UK
Ahmed M. Salman	Jenner Institute, Nuffield Department of Medicine, University of Oxford, UK
Natalia Salvador	Instituto D'Or de Pesquisa e Ensino (IDOR), Rio de Janeiro, Brazil
Stephannie Salvador	Jenner Institute, Nuffield Department of Medicine, University of Oxford, UK
Milla Dias Sampaio	Instituto D’Or de Pesquisa e Ensino (IDOR), Salvador, Brazil and Rede D’OR São Luiz, Brazil
Annette D Samson	Hull University Teaching Hospitals NHS Trust, Hull, UK
Amada Sanchez-Gonzalez	Department of Infection and Tropical Medicine, Newcastle upon Tyne Hospitals NHS Foundation Trust, UK
Helen Sanders	Jenner Institute, Nuffield Department of Medicine, University of Oxford, UK
Katherine Sanders	Oxford Vaccine Group, Department of Paediatrics, University of Oxford, UK
Erika E. P. D. Santos	Postgraduate Programme in Nursing, Universidade Federal de Santa Maria, Santa Maria, Brazil
Mayara F. S. Guerra	Instituto D'Or de Pesquisa e Ensino (IDOR), Rio de Janeiro, Brazil
Iman Satti	Jenner Institute, Nuffield Department of Medicine, University of Oxford, UK
Jack E. Saunders	Jenner Institute, Nuffield Department of Medicine, University of Oxford, UK

Caroline Saunders	NIHR Cambridge Clinical Research Facility, Cambridge, UK
Aakifah Bibi Arif Sayed	Perinatal HIV Research Unit, Faculty of Health Sciences, University of the Witwatersrand, Johannesburg, South Africa
Ina Schim van der Loeff	Department of Infection and Tropical Medicine, Newcastle upon Tyne Hospitals NHS Foundation Trust and Translational and Clinical Research Institute, Immunity and Inflammation Theme, Newcastle University
Annina B Schmid	Nuffield Department of Clinical Neurosciences, University of Oxford, UK
Ella Schofield	University of Oxford Medical School, Medical Sciences Division, University of Oxford, UK
Gavin R. Screaton	Medical Sciences, University of Oxford, UK
Samiullah Seddiqi	Oxford Vaccine Group, Department of Paediatrics, University of Oxford, UK
Rameswara R. Segireddy	Jenner Institute, Nuffield Department of Medicine, University of Oxford, UK
Roberta Senger	Hospital Universitário de Santa Maria, Santa Maria, Brazil
Sonia Serrano	NIHR BRC at Guy's and St Thomas' NHS Foundation Trust, UK
Imam Shaik	National Infection Service, Public Health England, UK
Hannah R. Sharpe	Jenner Institute, Nuffield Department of Medicine, University of Oxford, UK
Katherine Sharrocks	Department of Medicine, University of Cambridge, UK
Robert Shaw	Oxford Vaccine Group, Department of Paediatrics, University of Oxford, UK
Adam Shea	Jenner Institute, Nuffield Department of Medicine, University of Oxford, UK
Emma Sheehan	Jenner Institute, Nuffield Department of Medicine, University of Oxford, UK
Amy Shepherd	Clinical Infection Research Group, Regional Infectious Diseases Unit, NHS Lothian, UK
Farah Shiham	Department of Clinical Sciences, Liverpool School of Tropical Medicine and Liverpool University Hospitals NHS Foundation Trust, UK
Sarah E. Silk	Jenner Institute, Nuffield Department of Medicine, University of Oxford, UK
Ana Carolina Mesquita	Instituto D'Or de Pesquisa e Ensino (IDOR), Rio de Janeiro, Brazil and Hospital Quinta D'OR, Rede D'Or São Luiz, Rio de Janeiro, Brazil
Laura Silva-Reyes	Oxford Vaccine Group, Department of Paediatrics, University of Oxford, UK
Lidiana B. T. D. Silveira	Postgraduate Programme in Nursing, Universidade Federal de Santa Maria, Santa Maria, Brazil
Mariana B. V. Silveira	Universidade Federal de São Paulo, Brazil
Nisha Singh	Oxford Vaccine Group, Department of Paediatrics, University of Oxford, UK
Jaisi Sinha	Public Health Wales NHS Trust, Cardiff, UK
Donal T Skelly	Nuffield Department of Clinical Neurosciences, University of Oxford, UK
Daniel C. Smith	Cobra Biologics, Keele Science Park, UK
Nicholas Smith	Cobra Biologics, Keele Science Park, UK

Holly E. Smith	Jenner Institute, Nuffield Department of Medicine, University of Oxford, UK
David J Smith	Oxford Vaccine Group, Department of Paediatrics, University of Oxford, UK
Catherine C Smith	Oxford Vaccine Group, Department of Paediatrics, University of Oxford, UK
Airanuédida S. Soares	Centro de Estudos e Pesquisas em Moléstias Infecciosas, Rio Grande do Norte, Brazil
Carla Solórzano	Department of Clinical Sciences, Liverpool School of Tropical Medicine, UK
Guilherme L. Sorio	Hospital de Clinicas de Porto Alegre, Universidade Federal do Rio Grande do Sul, Brazil
Kim Sorley	NIHR Imperial Clinical Research Facility, London, UK
Tiffany Sosa-Rodriguez	Halix B.V., Tinbergenweg 1, 2333 BB Leiden, Netherlands
Cinthia M. C. D. L. Souza	Centro de Estudos e Pesquisas em Moléstias Infecciosas, Rio Grande do Norte, Brazil
Bruno S. D. F. Souza	Gonçalo Moniz Institute (Fiocruz); D'Or Institute for Research and Education (IDOR), Salvador, Brazil
Alessandra R. Souza	Universidade Federal de São Paulo, Brazil
Thamyres Souza Lopes	Instituto D'Or de Pesquisa e Ensino (IDOR), Rio de Janeiro, Brazil
Luciana Sowole	NIHR BRC at Guy's and St Thomas' NHS Foundation Trust, UK
Alexandra J Spencer	Jenner Institute, Nuffield Department of Medicine, University of Oxford, UK
Louise Spoors	Nuffield Dept of Orthopaedics Rheumatology and Musculoskeletal Medicine, University of Oxford, UK
Lizzie Stafford	Nuffield Department of Medicine, University of Oxford, UK
Imogen Stamford	Oxford University Hospitals NHS Foundation Trust, Oxford, UK
Ricardo Stein	Hospital de Clinicas de Porto Alegre, Universidade Federal do Rio Grande do Sul, Brazil
Lisa Stockdale	Oxford Vaccine Group, Department of Paediatrics, University of Oxford, UK
Lisa V. Stockwell	Oxford Vaccine Group, Department of Paediatrics, University of Oxford, UK
Louise H. Strickland	Nuffield Dept of Orthopaedics Rheumatology and Musculoskeletal Medicine, University of Oxford, UK
Arabella S. V. Stuart	Oxford Vaccine Group, Department of Paediatrics, University of Oxford, UK
Ann Sturdy	London Northwest University Healthcare, Northwick Park Hospital, London, UK
Natalina Sutton	Vaccine Institute, Institute of Infection & Immunity, St. Georges, University of London and St Georges University Hospitals NHS Trust, London, UK
Anna Szigeti	Oxford Vaccine Group, Department of Paediatrics, University of Oxford, UK
Abdessamad Tahiri-Alaoui	Clinical BioManufacturing Facility, Jenner Institute, University of Oxford, UK
Rachel Tanner	Jenner Institute, Nuffield Department of Medicine, University of Oxford, UK

Carol Taoushanis	VIDA - Vaccines and Infectious Diseases Analytical Research Unit, Diepkloof, Soweto, South Africa
Alexander W Tarr	School of Life Sciences, University of Nottingham, Nottingham, UK & NIHR Nottingham Biomedical Research Centre, Nottingham University Hospitals NHS Trust, Nottingham, UK
Richard Tarrant	Clinical BioManufacturing Facility, Jenner Institute, University of Oxford, UK
Keja Taylor	Clinical BioManufacturing Facility, Jenner Institute, University of Oxford, UK
Ursula Taylor	Department of Paediatrics, University of Oxford, UK
Iona Jennifer Taylor	Jenner Institute, Nuffield Department of Medicine, University of Oxford, UK
Justin Taylor	Oxford Vaccine Group, Department of Paediatrics, University of Oxford, UK
Rebecca te Water Naude	University of Oxford Medical School, Medical Sciences Division, University of Oxford, UK
Kate Templeton	NHS Lothian, Edinburgh, UK
Yrene Themistocleous	Jenner Institute, Nuffield Department of Medicine, University of Oxford, UK
Andreas Themistocleous	Nuffield Department of Clinical Neurosciences, University of Oxford, UK
Merin Thomas	Jenner Institute, Nuffield Department of Medicine, University of Oxford, UK
Kelly M Thomas	National Infection Service, Public Health England, UK
Tonia M Thomas	Oxford Vaccine Group, Department of Paediatrics, University of Oxford, UK
Asha Thombrayil	VIDA - Vaccines and Infectious Diseases Analytical Research Unit, Diepkloof, Soweto, South Africa
Julia Thompson	AstraZeneca BioPharmaceuticals PLC
Fawziyah Thompson	Centre for Lung Infection and Immunity, Division of Pulmonology, Department of Medicine and UCT Lung Institute & South African MRC/UCT Centre for the Study of Antimicrobial Resistance, University of Cape Town, Cape Town, South Africa
Ameeka Thompson	North Bristol NHS Trust, Bristol, UK
Amber J Thompson	Oxford Vaccine Group, Department of Paediatrics, University of Oxford, UK
Kevin P Thompson	Pall Europe Ltd, Harbourgate Business Park, Portsmouth, UK
Viv Thornton-Jones	Oxford University Hospitals NHS Foundation Trust, Oxford, UK
Larissa H. S. Thotusi	Universidade Federal de São Paulo, Brazil
Patrick J Tighe	School of Life Sciences, University of Nottingham, Nottingham, UK
Lygia Accioly Tinoco	Instituto D'Or de Pesquisa e Ensino (IDOR), Salvador, Brazil and Rede D'OR São Luiz, Brazil
Gerlynn Ferreras Tiongson	London Northwest University Healthcare, Northwick Park Hospital, London, UK
Bonolo Tladinyane	Setshababa Research Centre , Tshwane, South Africa
Michele Tomasicchio	Centre for Lung Infection and Immunity, Division of Pulmonology, Department of Medicine and UCT Lung Institute & South African

	MRC/UCT Centre for the Study of Antimicrobial Resistance, University of Cape Town, Cape Town, South Africa
Adriana Tomic	Oxford Vaccine Group, Department of Paediatrics, University of Oxford, UK
Susan Tonks	Oxford Vaccine Group, Department of Paediatrics, University of Oxford, UK
James Towner	University of Oxford Medical School, Medical Sciences Division, University of Oxford, UK
Nguyen Tran	Jenner Institute, Nuffield Department of Medicine, University of Oxford, UK
Julia A. Tree	National Infection Service, Public Health England, UK
Gerardo Trillana	NIHR BRC at Guy's and St Thomas' NHS Foundation Trust, UK
Charlotte Trinham	NIHR/Wellcome Trust Birmingham Clinical Research Facility, Birmingham, UK
Rose Trivett	Oxford Vaccine Group, Department of Paediatrics, University of Oxford, UK
Adam Truby	Jenner Institute, Nuffield Department of Medicine, University of Oxford, UK
Betty Lebogang Tsheko	Soweto Clinical Trials Centre, Johannesburg, South Africa
Philippa Tubb	Department of Clinical Sciences, Liverpool School of Tropical Medicine, Liverpool, UK
Aadil El-Turabi	Jenner Institute, Nuffield Department of Medicine, University of Oxford, UK
Richard Turner	AstraZeneca BioPharmaceuticals PLC
Cheryl Turner	Jenner Institute, Nuffield Department of Medicine, University of Oxford, UK
Nicola Turner	NIHR UCLH Clinical Research Facility, London, UK
Bhavya Tyagi	London Northwest University Healthcare, Northwick Park Hospital, London, UK
Marta Ulaszewska	Jenner Institute, Nuffield Department of Medicine, University of Oxford, UK
Benjamin R. Underwood	Windsor Research Unit, Cambridge and Peterborough NHS Foundation Trust, UK
Samual van Eck	Wits Reproductive Health and HIV Institute, Faculty of Health Sciences, University of the Witwatersrand, Johannesburg, South Africa
Rachel Varughese	Oxford Vaccine Group, Department of Paediatrics, University of Oxford, UK
Dennis Verbart	Halix B.V., Tinbergenweg 1, 2333 BB Leiden, Netherlands
Marije K Verheul	Oxford Vaccine Group, Department of Paediatrics, University of Oxford, UK
Iason Vichos	Oxford Vaccine Group, Department of Paediatrics, University of Oxford, UK
Taiane A Vieira	Hospital de Clinicas de Porto Alegre, Universidade Federal do Rio Grande do Sul, Brazil
Gemma Walker	Hull University Teaching Hospitals NHS Trust, Hull, UK
Laura Walker	Oxford Vaccine Group, Department of Paediatrics, University of Oxford, UK
Matthew E Wand	National Infection Service, Public Health England, UK

Theresa Wardell	Oxford Biomedica PLC, Transport Way, Cowley, Oxford
George M. Warimwe	KEMRI-Wellcome Trust Research Programme, Kenya and Centre for Tropical Medicine & Global Health, Nuffield Department of Medicine, University of Oxford, UK
Sarah C. Warren	NIHR Southampton Clinical Research Facility, Southampton, UK
Bridget Watkins	Nuffield Dept of Orthopaedics Rheumatology and Musculoskeletal Medicine, University of Oxford, UK
Marion E. E. Watson	Jenner Institute, Nuffield Department of Medicine, University of Oxford, UK
Ekaterina Watson	London Northwest University Healthcare, Northwick Park Hospital, London, UK
Stewart Webb	Department of Neurology, Queen Elizabeth University Hospital, Glasgow, UK
Andrea Webster	Research Directorate, Newcastle upon Tyne Hospitals NHS Foundation Trust, UK
Jessica Welch	Oxford University Hospitals NHS Foundation Trust, Oxford, UK
Zoe Wellbelove	Hull University Teaching Hospitals NHS Trust, Hull, UK
Jeanette H. Wells	Aneurin Bevan University Health Board, Newport, Wales, UK
Alison J. West	Oxford University Hospitals NHS Foundation Trust, Oxford, UK
Beth White	Department of Infectious Diseases, Queen Elizabeth University Hospital, Glasgow, UK
Caroline White	Oxford Vaccine Group, Department of Paediatrics, University of Oxford, UK
Rachel White	Oxford Vaccine Group, Department of Paediatrics, University of Oxford, UK
Paul Williams	Clinical BioManufacturing Facility, Jenner Institute, University of Oxford, UK
Rachel L Williams	Research & Innovation, North Bristol NHS Trust, Bristol, UK
Silvia Madrid Willingham	Hull University Teaching Hospitals NHS Trust, Hull, UK
Rebecca L. Winslow	NIHR/Wellcome Trust Birmingham Clinical Research Facility, Birmingham, UK
Danielle Woods	Jenner Institute, Nuffield Department of Medicine, University of Oxford, UK
Mark Woodyer	Oxford Biomedica PLC, Transport Way, Cowley, Oxford, UK
Andrew T. Worth	Jenner Institute, Nuffield Department of Medicine, University of Oxford, UK
Daniel Wright	Jenner Institute, Nuffield Department of Medicine, University of Oxford, UK
Marzena Wroblewska	Jenner Institute, Nuffield Department of Medicine, University of Oxford, UK
Xin Li Yao	Oxford Vaccine Group, Department of Paediatrics, University of Oxford, UK
Yee Ting Nicole Yim	NIHR UCLH Clinical Research Facility, London, UK
Marina Bauer Zambrano	Hospital de Clinicas de Porto Alegre, Universidade Federal do Rio Grande do Sul, Brazil
Rafael Leal Zimmer	Hospital de Clinicas de Porto Alegre, Universidade Federal do Rio Grande do Sul, Brazil

Dalila Zizi	Clinical BioManufacturing Facility, Jenner Institute, University of Oxford, UK
Peter Zuidewind	Family Centre for Research with Ubuntu, Department of Paediatrics, University of Stellenbosch, Cape Town, South Africa

Acknowledgements

**Advent, South Africa**
Michael Breese
**BioIndustry Association**
Annette England
Ian McCubbin
**Cell & Gene Therapy Catapult**
Stephen Ward
**CobraBio**
Mike Austin
Richard Condliffe
Peter Coleman
Steve Garland
Philip Ridley-Smith
**Clinical Trials Research Governance Office, University of Oxford**
Ronja Bahadori
Elaine Chick
Heather House
Claire Riddle
**Data and Safety Monitoring Board (DSMB)**
George Bouliotis
Steve Black
Elizabeth Bukusi
Cornelia Dekker
Robert Heyderman
Gregory Hussey
Paul Kaye
Bernhards Ogutu
Walter Orenstein
Sonia Ramos
Manish Sadarangani
**Deloitte UK**
Alex Hope
**Department of Health and Social Care, UK Government**
Harry Mayhew
Martin Shanahan

**Department of Paediatrics, University of Oxford**
Joanna Bagniewska
Elizabeth Derow
Georg A. Holländer
Samantha Vanderslott
**Endpoint Evaluation Committee**
Jeremy Carr
Stephen Chambers
Kim Davis
Simon Drysdale
Malick Gibani
Elizabeth Hammershaimb
Michael Harrington
Celina Jin
Seilesh Kadambari
Rama Kandasamy
Toby Maher
Jamilah Meghji
Claire Munro[1]
David Pace
Rekha R. Rapaka
Robindra Basu Roy
Daniel Silman
Gemma Sinclair
Jing Wang
**Halix**
Thijs Booiman
James Harris
Alex Huybens
**The Cambridge NIHR CRF COVID Vaccine Group**
**The GSTT NIHR CRF COVID Vaccine Group**
**The Imperial CRF COVID Vaccine Group**
**Jenner Institute, University of Oxford**
Iona Tarbet
**Nuffield Department of Medicine, University of Oxford**
Joshua Burgoyne
Richard Cornall
Richard Liwicki

Denis Murphy
Elizabeth Salter
Katherine Skinner
Philip Taylor
Oto Velicka
**Oxford Biomedica**
Andy Lewin
James Miskin
Pippa Radcliffe
Jason Slingsby
**Oxford Research Services (Contracts)**
Carly Banner
Sally Pelling-Deeves
Gary Priest
**Oxford University Hospitals NHS Trust**
Monique Andersson
Bruno Holthof
**Pall Europe**
Clive Glover
Peter Levison
**Public Affairs Directorate and Divisional Communication Team**
Alison Brindle
Alexander Buxton
James Colman
Chris McIntyre
Steve Pritchard
**Sartorius**
Zander Hack
**VMIC**

Simon Hoffman
Simon McEwen
Rachael Robbins
